# Changes in Bone Turnover and Bone Loss in HIV-Infected Patients Changing Treatment to Tenofovir-Emtricitabine or Abacavir-Lamivudine

**DOI:** 10.1371/journal.pone.0038377

**Published:** 2012-06-15

**Authors:** Hila Haskelberg, Jennifer F. Hoy, Janaki Amin, Peter R. Ebeling, Sean Emery, Andrew Carr, STEAL Study Group

**Affiliations:** 1 The Kirby Institute, University of New South Wales, Sydney, Australia; 2 Infectious Diseases Unit, The Alfred Hospital, Monash University, Melbourne, Australia; 3 North West Academic Centre, University of Melbourne, Melbourne, Australia; 4 St. Vincent’s Hospital, Sydney, Australia; University of Cape Town, South Africa

## Abstract

**Background:**

Those receiving tenofovir/emtricitabine (TDF-FTC) had greater bone loss compared with abacavir/lamivudine (ABC-3TC) in a randomized simplification trial (STEAL study). Previous studies associated increased bone turnover and bone loss with initiation of antiretroviral treatment, however it is unclear whether change in bone mineral density (BMD) was a result of specific drugs, from immune reconstitution or from suppression of HIV replication. This analysis determined predictors of BMD change in the hip and spine by dual-energy x-ray absorptiometry in virologically suppressed participants through week 96.

**Methodology/Principal Findings:**

Bone turnover markers (BTMs) tested were: formation [bone alkaline phosphatase, procollagen type 1 N-terminal propeptide (P1NP)]; resorption (C-terminal cross-linking telopeptide of type 1 collagen [CTx]); and bone cytokine-signalling (osteoprotegerin, RANK ligand). Independent predictors of BMD change were determined using forward, stepwise, linear regression. BTM changes and fracture risk (FRAX®) at week 96 were compared by t-test. Baseline characteristics (n = 301) were: 98% male, mean age 45 years, current protease-inhibitor (PI) 23%, tenofovir/abacavir-naïve 52%. Independent baseline predictors of greater hip and spine bone loss were TDF-FTC randomisation (p≤0.013), lower fat mass (p-trend≤0.009), lower P1NP (p = 0.015), and higher hip T score/spine BMD (p-trend≤0.006). Baseline PI use was associated with greater spine bone loss (p = 0.004). TDF-FTC increased P1NP and CTx through Wk96 (p<0.01). Early changes in BTM did not predict bone loss at week 96. No significant between-group difference was found in fracture risk.

**Conclusions/Significance:**

Tenofovir/emtricitabine treatment, lower bone formation and lower fat mass predicted subsequent bone loss. There was no association between TDF-FTC and fracture risk.

## Introduction

Highly active antiretroviral therapy (HAART) significantly improves survival and quality of life for individuals with human immunodeficiency virus (HIV) infection. However, clinical management challenges now include several disorders associated with aging, including higher prevalence of low bone mineral density (BMD) [1] and higher rates of fractures [2] than in adults without HIV. Prospective studies, mostly small and/or non-randomized, have generally found that ART initiation reduced BMD by 1% to 5% over 1 to 2 years (reviewed in [3]) although this initial short-term bone loss may not be ongoing [4]. These rates of bone loss appear greater than those expected in healthy men, being more similar to those in post-menopausal women [5]. Therapy with tenofovir (TDF) has been associated with greater reductions in BMD than with stavudine or abacavir (ABC) [6], [7], [8]. Bone metabolism can be assessed by measuring serum bone turnover markers (BTMs), comprising proteins synthesized during bone formation, bone matrix proteins, and bone collagen degradation products released during bone resorption. High levels of BTMs have been found to predict fractures independently of BMD in post-menopausal women [9] and elderly men [10]. Furthermore, as BTMs can provide a more dynamic estimate of bone metabolism in shorter timescales than BMD, these markers have been suggested as additional tools for more rapid assessment of bone disease [11]. Interleukin-1 (IL-1), IL-6, and tumour necrosis factor-alpha (TNF-α) can all increase bone resorption [12], and their circulating levels predict changes in BMD in HIV-uninfected adults [13]. Despite undetectable HIV viral load on ART, HIV-infected individuals have significantly higher levels of IL-6 compared with the general population [14].

BTMs have been found to be elevated in ART-treated compared with untreated patients in some cross-sectional studies [15], [16], [17]. In a trial of ART-naive patients, those randomized to TDF-emtricitabine (FTC) had greater bone loss and greater BTM increases over 24 weeks than those randomised to ABC-lamivudine (3TC) [7]. Other studies have shown that early increases in bone resorption were associated with BMD decreases and were followed by increased bone formation in ART-naïve adults, but it is not possible from these data to determine whether the BMD and BTM changes were a result of specific drugs, from immune reconstitution or from suppression of HIV replication [18], [19].

In the STEAL study, virologically suppressed patients randomized to simplify dual nucleoside analogue reverse transcriptase inhibitor (NRTI) therapy to coformulated TDF-FTC had greater bone loss over 96 weeks than those randomised to co-formulated ABC-3TC [20]. This analysis aimed to explore STEAL bone outcomes in more detail and to determine predictors of BMD change. We hypothesized that some patients might be at greater risk of TDF-related BMD loss over 96 weeks and that this greater loss might be predicted by either baseline or on-study BTM levels.

## Methods

### Study Design

STEAL was an open-label, prospective, randomized, non-inferiority study that compared simplification of current NRTIs to fixed-dose combination TDF-FTC or ABC-3TC over 96 weeks in 357 adults with plasma HIV viral load <50 copies/ml [20]. The protocol for this trial and supporting CONSORT checklist are available as supporting information; see Protocol S1, Protocol S2 and Checklist S1.

### Ethics

The study was approved by each site’s Human Research and Ethics Committee (30 sites) and registered at Clinicaltrials.gov (NCT00192634). Each participant signed a written informed consent before enrolment.

### Bone Mineral Density and Laboratory Markers

Dual-energy X-ray absorptiometry (DXA) of the lumbar spine and right hip were performed for each participant at the same imaging facility on the same bone densitometer, at baseline, week 48, and week 96, using a standardized protocol. BMD scans were not centrally analysed. DXA instruments varied between sites (GE-Lunar in 72% of sites); randomization was stratified by site, and therefore by model of DXA scanner.

Plasma and serum samples were collected at baseline and at weeks 12, 24, 48, 72 and 96 (following a 10-hour overnight fast, except at week 12) and stored at –70°C. Markers of bone resorption (C-terminal cross-linking telopeptide of type 1 collagen, [βCTX]; bone formation (procollagen type 1 N-terminal propeptide [P1NP]; bone-specific alkaline phosphatase,[BALP]) and regulators of bone turnover (osteoprotegerin [OPG] and receptor activator of nuclear factor kappa ligand [RANKL]) were evaluated. βCTX and P1NP were assayed by an electrochemiluminescence immunoassay (E170 immunoassay analyzer; Roche, Mannheim, Germany; reference ranges βCTX 170–600 ng/L, P1NP 16.3–78.2 ug/L). BALP, OPG and RANKL were assayed by Immunoenzymetric Assay (Manual with Plate Reader; Immunodiagnostic Systems, Boldon, United Kingdom; reference ranges BALP 8–21.3 ug/L, OPG <30 pmol/L, RANKL <100 pmol/L). The following were assessed at baseline only: Interleukin-6 by ELISA R&D Systems Human IL-6 (reference range 0.447–9.96 pg/ml); oestradiol by electrochemiluminescence (E170 immunoassay analyzer; Roche, reference range 50–200 pmol/L); free testosterone using total testosterone and sex hormone-binding globulin (Vermeulen formula [20]); and 25-hydroxy vitamin D by competitive chemiluminescence (Liasion; DiaSorin, Inc., Stillwater, MN, USA).

BTMs were batch-tested after study completion in one laboratory. Coefficients of variation were within accepted standard limits. The 10-year risks of osteoporotic and hip fractures were estimated using the FRAX® UK algorithm of the World Health Organization (WHO) [21]. The proportion of participants above the threshold recommended for intervention with antiresorptive therapy was determined according to the US National Osteoporosis Foundation (NOF) guidelines [22]. For the analysis of clinically relevant low BMD, low BMD was defined as T-score <−1 in accordance with WHO diagnostic thresholds [23].

### Statistical Analysis

Statistical analysis was conducted on the per protocol (PP) population, comprising all participants with BMD data and on randomized therapy at each time point. A PP approach was used to explore biological mechanisms of BMD change in response to study drug exposure. A pre-defined secondary analysis of this substudy was performed on the subpopulation that was not receiving ABC or TDF at study entry (“TDF/ABC-naïve”). Randomization was stratified by baseline NRTI use (TDF without ABC, ABC without TDF, or other), current protease inhibitor use, and study site. Absolute change in BMD at the lumbar spine and hip was the primary outcome of interest. Percent change was summarised as a secondary outcome.

The associations between baseline covariates (including demographic, HIV-related factors, ART, body composition, BTMs, bone remodelling regulators, sex hormones, vitamin D and IL-6; Table 1) and changes in BTMs from baseline to week 12, and absolute changes in hip and lumbar spine BMD to week 96 were analysed using linear regression. Three patients, all on TDF-FTC, started anti-resorptive therapy after week 48 and were included in the analysis. However, data on use of alcohol, glucocorticoids, vitamin D and calcium supplementation that can affect BMD were not collected. Multivariable models were built using forward, stepwise methods. Predictors that achieved a p-value <0.08 in univariate analysis (more conservative than 0.1) were assessed for inclusion in the multivariable model. Randomized groups were compared for changes in BTMs and FRAX® results by t-test at 48 weeks (BTMs only) and 96 weeks. Interaction between baseline exposure to TDF or ABC and randomized arm in predicting BTMs was tested using linear regression for the interaction term. Contingency-table and chi-square tests were used for analysis of proportions warranting antiresorptive-therapy (US NOF guidelines) and for incidence of low BMD. Sensitivity analysis was conducted for FRAX® results for participants who were at ≥40 years of age at baseline and for proportions warranting antiresorptive therapy (US NOF guidelines) for participants who are at ≥50 years of age at baseline. Pearson’s correlation was used to assess associations between changes in BTMs across the entire study population.

**Table 1:** Baseline characteristics.

Baseline Characteristic	ABC-3TC (n = 147)	TDF-FTC (n = 154)
Age (years)	45.8±8.7	44.7±8.3
Male (%)	99	97
Ethnicity - white (%)	84	86
HIV duration (years)	9.9±5.8	10.5±6.1
CD4+ count (cells/mm^3^)	623±300	604±262
IL6 (pg/ml)	2.2±2.0	1.9±1.4
**NRTI exposure**		
Prior ABC (n, %)	28 (19)	29 (19)
Prior TDF (n, %)	42 (29)	45 (29)
Naive to ABC and TDF (n,%)	77 (52)	80 (52)
NRTI duration (years)	5.7±3.4	5.7±3.5
PI duration (years)	2.0±2.7	1.9±2.7
NNRTI duration (years)	3.5±2.8	3.6±2.8
Current protease inhibitor (%)	23	24
**Anthropometric factors**		
Body mass index (kg/m2)	24.7±3.5	24.8±3.6
Fat mass (g)	15813±6970	16128±7901
**BMD**		
Right hip (g/cm^2^)	1.02±0.13	1.02±0.14
Spine (g/cm^2^)	1.18±0.16	1.19±0.16
**Bone resorption**		
βCTx (ng/L)	240.0±148.1	263.9±145.4
**Bone formation**		
BALP (µg/L)	20.2±10.2	19.8±11.6
P1NP (µg/L)	53.3±23.1	57.0±22.6
**Bone regulation**		
OPG (pmol/L)	3.9±1.3	3.8±1.1
RANKL (pmol/L)	0.2±0.3	0.3±0.4
**Sex hormones**		
Total testosterone (nmol/l)	17.5±7.7	17.7±7.7
Free testosterone (pmol/L)	295.9±123.6	291.0±125.8
25-hydroxy vitamin D (nmol/L)	71.2±30.4	67.5±30.0
Oestradiol (pmol/L)	91.5±39.5	90.6±34.4
**Ten-Year Fracture Risk** a		
Major OP fracture risk	3.3±0.1	3.3±0.1
Hip fracture risk	0.6±0.1	0.6±0.1

**Note**. Results are expressed as mean ± standard deviation or %.

Abbreviations: **ABC-3TC**, abacavir-lamivudine; **BALP**, bone-specific alkaline phosphatase; **βCTx**, C-terminal cross-linking telopeptide of type 1 collagen; **BMD**, bone mineral density; **NNRTI**, non-nucleoside reverse transcriptase inhibitor; **NRTI**, nucleoside reverse transcriptase inhibitor; **PI**, protease inhibitor; **OP**, osteoporotic; **OPG**, osteoprotegerin; **P1NP**, procollagen type 1 N-terminal propeptide; **RANKL**, Receptor Activator of Nuclear Factor Kappa Ligand; **TDF-FTC**, tenofovir-emtricitabine.

a According to FRAX® Scores Computed with BMD.

Statistical significance was defined as a 2-sided α of 0.05. All analyses of the main BMD and BTM outcomes were determined a priori and were hypothesis driven. No adjustment was made for multiple comparisons. Statistical analyses were performed with STATA, version 10.1 (Statacorp).

## Results

Of 357 participants enrolled in the parent study, 18 discontinued ABC-3TC and 16 discontinued TDF-FTC by week 96. An additional 22 participants (14 on ABC-3TC and 8 on TDF-FTC) did not have data for hip and spine BMD change from baseline to week 96. Therefore, the analysed per-protocol population comprised the remaining 301 participants (84% of main study population). Baseline characteristics of the population analysed were similar to main study [19] and well balanced between arms (Tables 1, 2).

**Table 2:** Bone mineral density and bone turnover markers outcomes, by treatment group.

Variable	Mean change from baseline to week 48						Mean change from baseline to week 96*					
	ABC-3TC	TDF-FTC	Mean difference(95% CI)	*P*	ABC-3TC (mean %)	TDF-FTC(mean %)	ABC-3TC	TDF-FTC	Mean difference(95% CI)	*P*	ABC-3TC(mean %)	TDF-FTC(mean%)
**BMD**												
Right hip(g/cm^2^)	−0.006	−0.013	0.007 (−0.005 to 0.019)	0.256	−0.6	−1.2	0.004	−0.007	0.011 (0.003 to 0.019)	0.006	0.4	−0.6
Spine (g/cm^2^)	0.005	−0.016	0.021 (0.011 to 0.030)	<0.001	0.5	−1.2	0.008	−0.005	0.013 (0.002 to 0.025)	0.017	0.8	−0.3
**Bone resorption**												
βCTx (ng/L)	7.3	89.8	−82.5 (−120.2 to −44.8)	<0.001	27.5	68.4	10.9	72.5	−61.6 (−97.4 to −25.7)	0.001	31.7	65.4
**Bone formation**												
BALP (µg/L)	−3.7	0.2	−3.9 (−6.5 to −1.4)	0.002	132.9	121.8	−3.1	−0.7	−2.5 (−5.0 to 0.1)	0.060	149	135.5
P1NP (µg/L)	−7.3	4.2	−11.6 (−15.7 to −7.4)	<0.001	−8.4	16.1	−8.4	−0.5	−7.9 (−12.9 to −2.9)	0.002	−8.0	8.6
**Bone regulation**												
OPG (pmol/L)	−0.2	0.0	−0.1 (−0.4 to 0.0)	0.074	−1.4	6.0	−0.2	0.1	−0.3 (−0.6 to 0.0)	0.077	0.5	7.7
RANKL (pmol/L)	−0.1	−0.1	0.02 (−0.04 to 0.09)	0.448	−27.0	−29.3	−0.1	−0.1	0.0 (−0.1 to 0.1)	0.695	−24.1	13.4
**Ten-Year Fracture Risk According to FRAX® Scores Computed with BMD**												
Major OP fracture risk	ND	ND	ND	ND	ND	ND	−0.3	−0.2	−0.1 (−0.3 to 0.1)	0.460	ND	ND
Hip fracture risk	ND	ND	ND	ND	ND	ND	−0.2	−0.1	−0.1 (−0.2 to 0.1)	0.361	ND	ND
**Clinical implication**												
Reaching NOF criteria (%)^a^	ND	ND	ND	ND	ND	ND	0.6	2.5	−1.8 (−4.6 to 0.9)	0.371	ND	ND
Low femoral BMD (%)^b^	ND	ND	ND	ND	ND	ND	3.8	8.7	−4.8 (−10.4 to 0.4)	0.06	ND	ND
Low spine BMD (%)^b^	ND	ND	ND	ND	ND	ND	3.8	7.5	−3.6 (−8.7 to 1.4)	0.125	ND	ND

**Note**. Abbreviations: **ABC-3TC**, abacavir-lamivudine; **BALP**, bone-specific alkaline phosphatase (*n = 270); **βCTx**, C-terminal cross-linking telopeptide of type 1 collagen (*n = 281); **BMD**, bone mineral density; **CI**, confidence interval; **ND**, not done; **NOF**, National Osteoporosis Foundation; **OP**, osteoporotic; **OPG**, osteoprotegerin (*n = 270); **P1NP**, procollagen type 1 N-terminal propeptide (*n = 281); **RANKL**, Receptor Activator of Nuclear Factor Kappa Ligand (*n = 270); **TDF-FTC**, tenofovir-emtricitabine.

a proportions of participants above the thresholds recommended for antiresorptive therapy according to US NOF guidelines.

b low BMD defined as T-score <−1.

### Bone Mineral Density Change from Baseline

**Figure 1:**
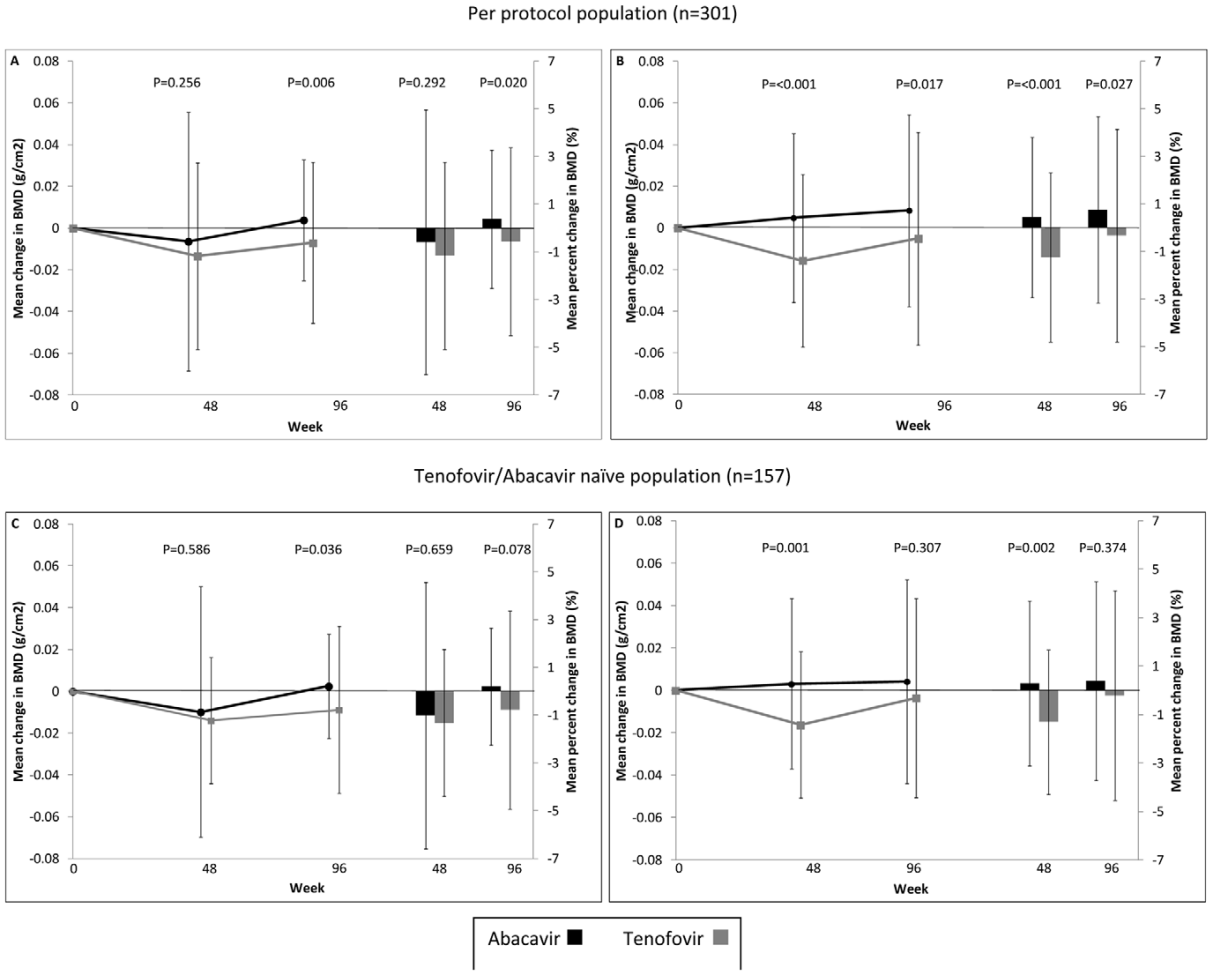
Mean change from baseline to week 96 in right hip (A,B) and lumbar spine bone mineral density (C,D) by study population and randomised arm (abacavir-lamivudine vs. tenofovir-emtricitabine). The right hand side of each graph shows the mean percent change in BMD at weeks 48 and 96. **Note.** p values from t-test comparing mean change from baseline to study week of interest in randomized arms. Error bars represent 1 standard deviation from the mean.Abbreviations: **ABC/3TC**, abacavir/lamivudine; **TDF/FTC**, tenofovir/emtricitabine.

At week 96, the absolute change from baseline in hip BMD in the ABC-3TC group was 0.004 g/cm^2^ versus −0.007 g/cm^2^ in the TDF-FTC group (treatment difference 0.01 g/cm^2^ (95% confidence interval [CI] 0.003 to 0.018; p = 0.006). For lumbar spine, the absolute BMD change over 96 weeks in ABC-3TC group was 0.008 g/cm^2^ and −0.005 g/cm^2^ in the TDF-FTC group (treatment difference 0.01 g/cm^2^ (95% CI 0.002 to 0.024; p = 0.016). BMD changes in the ‘TDF/ABC-naïve’ subpopulation, were of similar magnitude (Table 2, Figure 1).

### Baseline Predictors of Change in Bone Mineral Density

Baseline covariates significantly associated with greater decline in hip BMD over 96 weeks in multivariable analysis were TDF-FTC randomisation (p = 0.001), lower fat mass (p trend = 0.009), lower P1NP (p = 0.015), and higher hip T-score (p trend = 0.006). Baseline predictors of greater decline in spine BMD were TDF-FTC randomisation (p = 0.013), lower fat mass (p trend = 0.005), protease inhibitor use (p = 0.004), and higher spine BMD (p = 0.001; Table 3).

**Table 3:** Baseline covariates assessed in the multivariate model of change in right hip and lumbar spine bone mineral density over 96 weeks.

	Right hip Multivariate Analysis				Lumbar Spine Multivariate Analysis			
Baseline Covariate	Coef.	95% confidence interval	P	P trend	Coef.	95% Confidence Interval	P	P trend
**TDF-FTC randomisation**	−0.01	−0.02 to −0.01	**0.001**		−0.01	−0.02 to −0.00	**0.013**	
**Right Hip T score wk0– WHO categories:**								
≤−2.5*	reference							
−2.5<t<−1.0	−0.02	−0.04 to 0.01	0.166					
≥−1.0	−0.03	−0.05 to −0.00	0.031	**0.006**				
**Fat mass wk0 - quartiles:**								
1110–10590 g	−0.015	−0.03 to −0.00	0.005		−0.02	−0.03 to −0.00	0.023	
10591–15428 g	−0.004	−0.02 to 0.01	0.424		−0.01	−0.02 to 0.01	0.271	
15429–20942 g	−0.002	−0.01 to 0.01	0.711		0.01	−0.01 to 0.02	0.489	
20943–46433 g*	reference			**0.009**	reference			**0.005**
missing	−0.049	−0.11 to 0.02	0.134		0.01	−0.08 to 0.11	0.787	
**P1NP wk0**	0.001	0.00 to 0.00	**0.015**					
**PI at Baseline**					−0.02	−0.03 to −0.01	**0.004**	
**L1**–**L4 Spine BMD wk0 - quartiles:**								
0.788–1.061*					reference			
1.062–1.180					−0.02	−0.03 to −0.00	0.018	
1.181–1.292					−0.01	−0.03 to 0.00	0.057	
1.293–1.798					−0.03	−0.04 to −0.01	<0.001	**0.001**

**Note**. Baseline covariates from the univariate analysis that were assessed in multivariate model and not included in the final model for the hip were N(t)RTI duration, NRTI duration, femoral BMD, free testosterone, P1NP change from baseline to week 12, CTx change from baseline to week 12; for the spine: PI duration, NRTI duration, alkaline phosphatase, spine T score, hip T score;

Abbreviations: **PI,** protease inhibitor; **P1NP**, procollagen type 1 N-terminal propeptide; **TDF-FTC**, Tenofovir-Emtricitabine.

### Bone Turnover Markers

**Figure 2:**
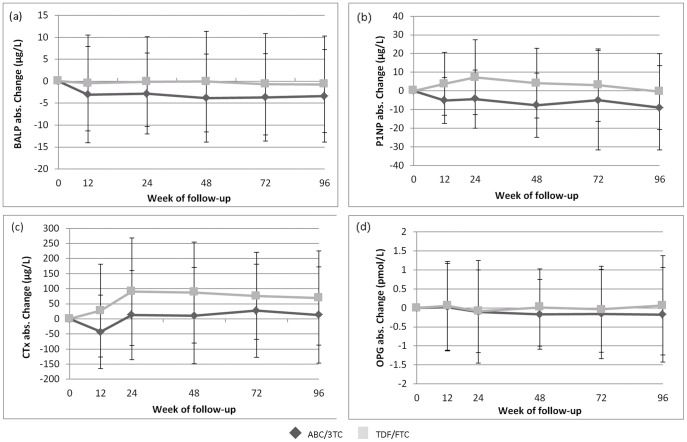
Mean change from baseline to week 96 in bone turnover markers by randomised arm (abacavir-lamivudine vs. tenofovir-emtricitabine). **Note.** Error bars represent 1 standard deviation from the mean (**a**) **BALP**, bone-specific alkaline phosphatase; (**b**) **P1NP**, procollagen type 1 N-terminal propeptide; (**c**) **βCTx**, C-terminal cross-linking telopeptide of type 1 collagen; (**d**) **OPG**, osteoprotegerin; **ABC/3TC**, abacavir/lamivudine; **TDF/FTC**, tenofovir/emtricitabine. There was no significant between-group difference at any time point for RANK.

Significant differences in absolute changes in bone resorption and formation markers were seen after baseline between treatment groups. In the PP population, βCTx (bone resorption marker) increased significantly at week 12 in TDF-FTC compared to ABC-3TC arm (treatment difference 71.8 ng/L (95% CI 40.2 to 103.4; p<0.001)) and then remained stable through week 96. Similarly, increases in bone formation markers were greater with TDF-FTC than with ABC-3TC arm; P1NP was increased at week 12 (difference 8.79 µg/L [95% CI 5.48 to 12.10; p<0.001]) and remained stable thereafter. Another formation marker, BALP, was significantly different from week 24 onwards with greater increases with TDF-FTC than with ABC-3TC (difference 2.83 µg/L [95% CI 0.59 to 5.07; p = 0.014]) (Table 3, Figure 2). There was no significant, between-group difference in OPG or RANKL. Similar results were found in the ABC/TDF-naive subpopulation (data not shown). Changes in the bone resorption marker, βCTx, were correlated at all time points with changes in P1NP, a formation marker (r>0.30, p<0.001). Changes in BALP were correlated with changes in βCTx from week 24 onwards (r>0.15, p<0.014) except at week 48.

### Clinical Implications

There was no difference in FRAX® scores at 96 weeks either within each group or between groups. The FRAX® scores at week 96 for ten-year fracture risk for major osteoporotic fracture were: 3% for both ABC-3TC and TDF-FTC; hip fracture 0.4% with ABC-3TC vs. 0.5% with TDF-FTC in the PP population. There was no significant difference in the proportion (0.6% in ABC-3TC vs. 2.5% in TDF-FTC) of participants who met the NOF guidelines criteria for treatment by week 96. Similarly, there was no significant between-group difference in the incidence of participants who developed low hip BMD (T score ≤−1; 3.8% in ABC-3TC vs. 8.7% in TDF-FTC) or low spine BMD (3.8% in ABC-3TC vs. 7.4% in TDF-FTC) by week 96.

## Discussion

Small, yet significant differences between TDF-FTC and ABC-3TC in changes in absolute BMD values were found in our study, with greater bone loss in the TDF-FTC group over 96 weeks. Independent predictors for bone loss at both the hip and lumbar spine included TDF-FTC randomisation and lower baseline fat mass. Further, lower serum P1NP (bone formation marker) predicted greater hip bone loss, while protease inhibitor use at baseline predicted greater spine bone loss. BTMs significantly increased from week 12 with TDF-FTC, but their early changes (at week 12) did not predict subsequent bone loss. The clinical relevance of these changes is unknown.

Both the ASSERT study and ACTG 5224s, in ART-naive adults, found similar results over 48 weeks, showing greater increases in bone turnover [7] and decrease in BMD [7], [8] in participants randomised to TDF-FTC compared with ABC-3TC. The greater BMD loss observed in ASSERT compared with our study may in part be due to different populations (ASSERT participants were younger, ART-naïve, more immunodeficient and more ethnically diverse).

The association found between protease inhibitor use and spine bone loss confirms findings by other investigators [8], [24]. The spine comprises mainly trabecular bone, which is affected earlier than cortical bone (femoral neck) by high bone turnover [25]. Protease inhibitors can reduce calcium deposition and alkaline phosphatase expression in osteoblasts [26], both markers of osteoblast differentiation. This effect may explain why PINP, a bone formation marker primarily expressed during osteoblast proliferation, was more sensitive in detecting TDF-FTC effects on bone loss. We showed PINP increased with TDF-FTC treatment, while BAP did not, and lower PINP levels predicted greater bone loss from the hip, but not the spine. The mechanisms underlying the effect of TDF on increasing bone loss have not been clearly established, although recent *in vitro* studies suggest TDF may alter gene expression in both osteoblasts and osteoclasts [27], [28]. Furthermore, in similar settings without viraemia, a pre-exposure prophylaxis study found that initiation of TDF is also associated with bone loss, though bone turnover was not reported [29].

Higher baseline hip T-score and spine BMD predicted greater bone loss from the hip and spine, respectively in our study, an observation reported previously in the setting of allogeneic bone marrow transplantation [30]. This finding provides reassurance that patients with low BMD are not particularly at risk of greater bone loss when receiving TDF. The findings regarding the effect of lower fat mass on bone loss are supported by similar evidence in the general population, suggesting a link between body composition and bone density and the protective role of obesity on bone [31]. Other studies in HIV-infected adults have reported that low weight and BMI were associated with low BMD [32], [33], but we did not observe this association.

BTM levels increase following ART initiation [7], [19]. One study found reductions in bone formation with increased resorption, which increased with more advanced untreated HIV infection, suggesting “uncoupling” of the usually well regulated processes of bone resorption and formation [17]. After ART initiation, a “recoupling” of bone formation and resorption appears to occur, albeit with higher levels of bone turnover [17]. It is not clear whether the early changes in BTMs after ART initiation reflect the effects of specific ART drugs, HIV suppression or immune reconstitution. Our study is the first to report BTM changes with ART switching in stable, virologically suppressed individuals.

We also evaluated whether baseline BTM levels and early BTM changes can predict BMD change with ART. Changes in markers to week 12 were not associated with BMD decrease at week 96. Only lower baseline levels of one bone formation marker (P1NP) predicted greater bone loss at the hip. BTMs were significantly increased in the TDF-FTC arm compared with ABC-3TC, as seen in the ASSERT study [7]. These changes occurred early in the study (week 12) and bone turnover remained significantly higher in those randomized to TDF-FTC compared with ABC-3TC through 96 weeks. This is in contrast to the ASSERT study findings of similar bone resorption marker levels from week 48 onwards. Both studies show that formation and resorption remained coupled during study follow-up.

Although we could not assess fracture rates in this study, we explored other clinical implications by evaluating the 10-year fracture risk using the FRAX® algorithm and the incidence of participants above the FRAX threshold recommended for antiresorptive therapy according to NOF guidelines. No significant difference was found between TDF-FTC and ABC-3TC, even in analyses restricted to the older patients for which FRAX and NOF guidelines were designed, implying limited clinical significance. It is likely, however, that in addition to the small changes in BMD, the study was underpowered to detect significant risk differences between the groups. In addition, the FRAX equation requires additional clinical data not collected in STEAL (prior personal history of fracture, prior rheumatoid arthritis, family history of hip fracture, alcohol use). Therefore, the fracture risk may have been underestimated. The NOF guidelines were developed and validated in postmenopausal women and men aged at least 50 years, so the NOF estimates derived in this study should be viewed more in terms of the difference between groups rather than the absolute risk in each group. Lastly, FRAX and the NOF guidelines have not been validated in HIV-infected populations.

Our study has limitations. Our cohort was at low overall risk (average age 45 years, predominantly male) for bone-related harm as demonstrated by small BMD changes, with limited statistical power to determine predictors of change. In addition, as participants were on different regimens when entering the study, the sizes of the different sub-populations according to their baseline NRTI and PI agents were small, with attenuated statistical power for secondary analyses. The relatively short duration of the study and the small sample size did not allow for investigation of fractures and a possible association with the risk factors found. DXA scans were not centrally read to minimise measurement variability. An earlier time-point (e.g. 4 weeks) for BTM measurement may have provided more insight into the early pathophysiological effect of ART on bone metabolism.

Our study suggests that HIV-infected adults may benefit from assessment of risk factors associated with fractures prior to switching to TDF-FTC because of the associated higher bone turnover, and adults with a low fat mass are at higher risk for bone loss. In addition, measurement of P1NP might be useful in predicting greater hip bone loss with TDF-FTC, but this requires confirmation in larger studies.

## Supporting Information

Protocol S1 **A randomised, open-label trial to assess the safety and efficacy of switching to fixed-dose tenofovir-emtricitabine or abacavir-lamivudine: the STEAL study.**
(PDF)

Protocol S2 **STEAL BMD Sub-Study - Concept Sheet.**
(PDF)

Checklist S1 (DOCX)
