# Supplementary material for: Changes in Bone Turnover and Bone Loss in HIV-Infected Patients Changing Treatment to Tenofovir-Emtricitabine or Abacavir-Lamivudine
Source: PLoS One. 2012 Jun 15;7(6):e38377. doi: 10.1371/journal.pone.0038377 (PMC3376146; doi:10.1371/journal.pone.0038377)
Supplement: Protocol S1 — A randomised, open-label trial to assess the safety and efficacy of switching to fixed-dose tenofovir-emtricitabine or abacavir-lamivudine: the STEAL study. (PDF) [file pone.0038377.s001.pdf]

A randomised, open-label trial to assess the safety and efficacy of switching to fixed-dose tenofovir-emtricitabine or abacavir-lamivudine: the STEAL study.

Original protocol date: 19th August 2005

Protocol version: Version 5.0

Amendment date: 2nd July 2008

Name and address of sponsor:

National Centre in HIV Epidemiology and Clinical Research (NCHECR)

University of New South Wales (UNSW)

Sydney, Australia

Telephone: +61 2 9385 0900

Facsimile: +61 2 9385 0910

Email: steal@nchecr.unsw.edu.au

Title and name of principal investigator and site address/telephone numbers:

A/Prof Andrew Carr
HIV, Immunology and Infectious Diseases Unit
St Vincent's Hospital
Level 4, Xavier Building
Darlinghurst, NSW, 2010
Australia
Telephone: + 61 2 8382-3359
Facsimile: + 61 2 8382-2090
Email: acarr@stvincents.com.au

Protocol Steering Committee

Professor David A. Cooper
Director
NCHECR
Level 2, 376 Victoria Street
Darlinghurst, NSW, 2010
AUSTRALIA
Telephone: + 61-2 9385 0900
Facsimile: + 61-2 9385 0920
Email: dcooper@nchecr.unsw.edu.au

A/Prof Andrew Carr
Staff Specialist
HIV, Immunology and Infectious Diseases Unit
St Vincent's Hospital
Level 4, Xavier Building
Darlinghurst NSW 2010
Telephone: + 61-2 8382-3359
Facsimile: + 61-2 8382-2090
Email: acarr@stvincents.com.au

Associate Professor Sean Emery
Head, Therapeutic and Vaccine Research Program
NCHECR
Level 2, 376 Victoria Street
Darlinghurst, NSW, 2010
AUSTRALIA
Telephone: + 61-2 9385 0900
Facsimile: +61-2 9385 0910
Email: semery@nchecr.unsw.edu.au

Dr Nick Medland
The Centre Clinic
Rear 77 Fitzroy Street
St Kilda VIC 3182
AUSTRALIA
Telephone: + 61-3 9525-5866
Facsimile: + 61-3 9525-3673
Email: nick_medland@vicaids.asn.au

Dr David Baker
407 Doctors
407 Bourke Street
Surry Hills, NSW 2010
AUSTRALIA
Telephone: + 61-2 9332-2531
Facsimile: + 61-2 9380-5114
Email: db@407.com.au

Dr Mark Bloch
Holdsworth House Medical Practice
Suite1
32A Oxford Street
Darlinghurst, NSW 2010
Telephone: +61 -2 9331-7228
Facsimile: +61 -2 9331-5705
Email: MarkBloch@holdsworthhouse.com.au

Janaki Amin
Statistician
NCHECR
Level 2, 376 Victoria Street
Darlinghurst, NSW, 2010
AUSTRALIA
Telephone: + 61-2 9385 0900
Facsimile: +61-2 9385 0920
Email: jamin@nchecr.unsw.edu.au

Allison Humphries
Senior Clinical Project Coordinator
Therapeutic and Vaccine Research Program
NCHECR
Level 2, 376 Victoria Street
Darlinghurst, NSW, 2010
AUSTRALIA
Telephone: + 61-2 9385 0900
Facsimile: +61-2 9385 0910
Email: ahumphries@nchecr.unsw.edu.au

Sites and Principal Investigators

407 Doctors
Dr. David Baker
407 Bourke Street
Surry Hills, NSW, 2010
Australia
Telephone: + 61 2 9332-2531
Facsimile: + 61 2 9380-5114
Email: db@407.com.au

AIDS Medical Unit
Dr. Mark Kelly
2nd Floor, Biala
270 Roma Street
Brisbane, QLD, 4002
Australia
Telephone: +61 7 3224-5526
Facsimile: + 61 7 3224-5827
Email: markdeniskelly@bigpond.com

Albion Street Centre
Assoc. Prof. Don Smith
150 Albion Street
Darlinghurst, NSW, 2010
Australia
Telephone: + 61 2 9332-9656
Facsimile: + 61 2 9332-4219
Email: Dsmith@nchecr.unsw.edu.au

Burwood Clinic
Dr. Nicholas Doong
8 Burwood Road
Burwood, NSW, 2134
Australia
Telephone: + 61 2 9745-2755
Facsimile: + 61 2 9744-7927
Email: drndoong@bigpond.net.au

Care and Prevention Programme
Dr William O’Donohue
17 O'Brien Street
Adelaide, SA 5000
Telephone: +61 8 8231-4026
Facsimile: +61 8 8231-1211
Email: gary@careandprevention.org

Carlton Clinic
Dr. Jonathan Anderson
88 Rathdowne Street
Carlton, VIC, 3053
Australia
Telephone: + 61 3 9347-9422
Facsimile: + 61 3 9349-2991
Email: richard.moore@maynegroup.com

Centre Clinic
Dr. Nicholas Medland
Rear 77 Fitzroy Street
St. Kilda, VIC, 3182
Telephone: + 61 3 9525-5866
Facsimile: + 61 3 9525 3673
Email: nick_medland@vicaids.asn.au

Clinic 16
Dr Linda Dayan
Royal North Shore Hospital
16 Herbert St
St Leonards, 2065
Telephone: + 61 2 9926-7414
Facsimile + 61 2 9926-5582
Email: ldayan@nsccahs.health.nsw.gov.au

Dolls House
Dr Darren Russell
Sexual Health Services
The Doll's House Sexual Health Clinic
Cairns Base Hospital
The Esplanade
CAIRNS QLD 4870
Telephone: + 61 7 4050-6205
Facsimile + 61 7 4050-6359
Email: darren_russell@health.qld.gov.au

Flinders Medical Centre
Dr Mark Boyd
Flinders Medical Centre
Flinders Drive
Bedford Park SA 5042
Telephone: +61 8 8204 4292
Facsimile: +61 8 8204 4733
Email: mark.boyd@fmc.sa.gov.au

Fremantle Hospital
Dr John Dyer
Infectious Diseases Department
Level 2, B Block
Alma Steet
Fremantle WA 6160
Telephone: +61 8 9431-2274
Facsimile: +61 8 9431-2035
Email: john.dyer@health.wa.gov.au

Gladstone Medical Centre
Dr. David Orth
38 Gladstone Road
Highgate Hill, QLD, 4101
Australia
Telephone: + 61 7 3844-9599
Facsimile: + 61 7 3846-2957
Email: grmc1@powerup.com.au

Gold Coast Sexual Health Clinic
Dr. John Chuah
2019 Gold Coast Highway
Miami QLD 4220
Australia
Telephone: + 61 7 5576-9031
Facsimile: + 61 7 5576-9041
Email: chuahj@health.qld.gov.au

Holdsworth House Medical Practice
Dr. Mark Bloch
Suite1
32A Oxford Street
Darlinghurst, NSW, 2010
Australia
Telephone: + 61 2 9331-7228
Facsimile: + 61 2 9360-9232
Email: MarkBloch@holdsworthhouse.com.au

HHMP – Byron Bay
Dr Mark Bloch
37 Fletcher St
Byron Bay
NSW 2481
Australia
Telephone: + 61 2 6680 7211
Facsimile: + 61 2 6680 7311
Email: MarkBloch@holdsworthhouse.com.au

Lismore Sexual Health Clinic
Dr David Smith
Lismore Sexual Health & AIDS Services
66 Hunter Street
Lismore, NSW 2480
Telephone: + 61 2 6620-2980
Facsimile: + 61 2 6620-2985
Email: davidsm@nrhs.health.nsw.gov.au

Liverpool Hospital
Dr John Quin
Director, Sexual Health, HIV Medicine
Liverpool Health Service Immunology
Bigge Park Centre
PO Box 103
Liverpool NSW 2170
Telephone: + 61 2 9827 8022
Facsimile: + 61 2 9602 4352
Email: john.quin@swsahs.nsw.gov.au

Melbourne Sexual Health Centre
Dr. Tim Read
580 Swanston Street
Carlton, VIC, 3053
Australia
Telephone: + 61 3 9347-0244
Facsimile: + 61 3 9347-2230
Email: tread@mshc.org.au

Monash Medical Centre
Dr Ian Woolley
Monash Medical Centre
L4, 246 Clayton Road
Clayton, VIC 3168
Australia
Telephone: + 61 3 9594-6666
Facsimile: + 61 3 9594-4124
Email: ian.woolley@med.monash.edu.au

Prahran Market Clinic
Dr. Norman Roth
131 Commercial Road
South Yarra, VIC, 3141
Australia
Telephone: + 61 3 9826-4500
Facsimile: + 61 3 9826-4499
Email: njroth@office.net.au

Prince of Wales Hospital
Dr Jeffrey Post
Department of Infectious Diseases
Level 4, Dickinson Building
Prince of Wales Hospital
Randwick, NSW 2031
Telephone: + 61 2 9332-1090
Facsimile: + 61 2 9332-4129
Email: postje@sesahs.nsw.gov.au

Royal Adelaide Hospital
Dr David Shaw
Infectious Diseases Unit
Royal Adelaide Hospital
North Terrrace
Adelaide SA 5000
Telephone: + 61 8 8222 5635
Facsimile: + 61 8 8222 5888
Email: david.shaw@imvs.sa.gov.au

Royal Brisbane Hospital
A/Professor Anthony Allworth
Infectious Diseases Unit
Wattlebrae Day Therapy Unit
Royal Brisbane Hospital
Herston Road
Herston, QLD 4029
Telephone: + 61 7 3636-8155
Facsimile: + 61 7 3253-1388
Email: Tony_Allworth@health.qld.gov.au

Royal Melbourne Hospital
Dr. Alan Street
Deputy Director
Royal Melbourne Hospital
C/o Post Office, RMH
Parkville, VIC 3052
Telephone: + 61 3 9342-7212
Facsimile: + 61 3 9342-7277
Email: Alan.Street@mh.org.au

Royal Perth Hospital
Dr David Nolan
Department of Clinical Immunology
Level 2, North Block
Wellington Street
Perth, WA, 6000
Australia
Telephone: + 61 8 9224-2899
Facsimile: + 61 8 9224-2920
Email: D.Nolan@murdoch.edu.au

St Vincent's Hospital
Prof. David A. Cooper
Immunology B Clinic
Level 4, Xavier Building
Darlinghurst, NSW, 2010
Australia
Telephone: + 61 2 9385-0900
Facsimile: + 61 2 9385-0920
Email: dcooper@nchecr.unsw.edu.au

Taylor Square Clinic
Dr Robert Finlayson
Taylor Square Private Clinic
393 Bourke Street
Darlinghurst, NSW, 2010
Australia
Telephone: + 61 2 9331-6151
Facsimile: + 61 2 9331-3943
Email: robfin@medemail.com.au

The Alfred Hospital
Assoc. Prof. Jenny Hoy
Clinical Research -Infectious Diseases Unit
Second Floor Burnet Institute
Commercial Road
Melbourne, VIC, 3004
Australia
Telephone: + 61 3 9276-6900
Facsimile: + 61 3 9529-7019
Email: jennifer.hoy@med.monash.edu.au

Westmead Hospital
Dr Dominic Dwyer
CIDMLS, ICPMR
Westmead Hospital
Hawkesbury Road
Westmead, NSW 2145
Telephone: + 61 2 9845-6694
Facsimile: + 61 2 9845-8346
Email: dominic_dwyer@wmi.usyd.edu.au

Investigator Agreement and Signature Page
between the NCHECR, the Sponsor (if applicable) and the study investigator (s)

Principal Investigator:

Co-investigators (please list, if applicable)

This study will compare the overall safety and efficacy of simplified dual-NRTI-based ART with either once-daily ABC-3TC FDC or once-daily TDF-FTC FDC. The study will be conducted over 2 years in HIV-infected adults currently receiving two NRTIs as part of suppressive ART and is expected to involve 350 eligible participants.

Clinical work commenced in December 2005. This trial protocol, ICH Guidelines on Good Clinical Practice, the NCHECR standard operating procedures, the Therapeutic Goods Administration Access to Unapproved Therapeutic Goods – Clinical Trials in Australia regulations and the Declaration of Helsinki will be adhered to in all cases.

All participant data will be completely and accurately recorded in the participant case record forms. Completed case record forms will be submitted to NCHECR following study visits. Participant medical records will also be kept up-to-date regarding study information. The trial monitor will be allowed to review the participant case record forms, the source medical records, the trial progress and the consent forms. In addition, the sponsor’s auditors and regulatory authority auditors will be allowed access to all study documents, including source documents, if necessary.

Before entering this study, all participants, or in special cases, their legal representative or guardian will give their written informed consent. The signed consent forms will be retained by the investigator(s)/co-investigator(s) listed below.

I/We accept responsibility for the conduct of the research detailed in the proposal including all protocol-specific assessments, and I/We agree to abide by all decisions made by our Ethics Committee.

I/We agree to the above, which, in conjunction with the NHMRC Statement on Ethical Conduct in Research Involving Humans, will serve as the basis for co-operation in this study.

RESPONSIBLE INVESTIGATOR
(signature and date)

____

CO-INVESTIGATORS
(signature and date)

____

SPONSOR’S REPRESENTATIVE
(signature and date)

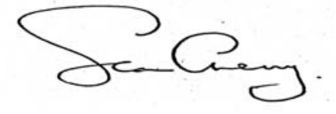

NCHECR MONITOR(S)/COORDINATOR (S)

____

Table of Contents

Protocol synopsis 9
Study flow chart 13

1.0 Introduction 14
1.1 Background and rationale 14
1.2 Summary of information on 3TC-ABC 17
1.3 Summary of information on FTC-TDF 18
1.4 Research Hypothesis 19
2.0 Trial objectives 19
2.1 Primary Objective 19
2.2 Secondary Objectives 19
3.0 Participant population 19
3.1 Inclusion Criteria 20
3.2 Exclusion Criteria 20
3.3 Study Drug Discontinuation 21
3.4 Premature Cessation of the Study 21
4.0 Trial design 22
4.1 Summary of Study Design 22
4.2 Study Conduct 23
5.0 Treatment of subjects 23
5.1 Treatment 23
5.2 Treatment Failure 24
5.3 Prohibited Concomitant Medications 24
5.4 Toxicity Management 25
6.0 Study procedures 28
6.1 Initial Screening Period 28
6.2 Randomisation Visit 30
6.3 Follow-up Visits 31
7.0 Data Safety and Monitoring Board (DSMB) 33
8.0 Recording and reporting adverse events 34
8.1 Recording Adverse Events 34
8.2 Reporting Adverse Events 35
8.3 Protocol-Specific Adverse Events 35
9.0 Packaging, labeling and accountability of trial supplies 39
9.1 Packaging and Labelling 39
9.2 Handling and Dispensing of Study Drugs 40
9.3 Return and Destruction of Study Drug 40
10.0 Biological samples 40
10.1 Blood collection 40
10.2 Labelling of Specimen Tubes 41
10.3 Transportation of Samples to Pathology 41
10.4 Storage of Samples 41
10.5 Processing of Samples 42
10.6 Reporting of Results 42
10.7 Plasma HIV RNA Determination 42
11.0 Statistics 42
12.0 Data collection, source documents and record retention 44
13.0 Ethics committee/regulatory approval and informed consent 46
14.0 Confidentiality of data 47
14.1 Confidentiality of Participant Records 47
14.2 Confidentiality of Study Data 47
15.0 Financing and insurance 47
16.0 Quality control and quality assurance 48
17.0 Publication policy 48

List of references 50

Appendices 52
1. Definitions and Criteria for HIV Disease and AIDS Events 52
2. Grading of Adverse Events 56
3. Sample Participant Information/Consent Form 70
4. STEAL Extension – Characterisation of participants clinically diagnosed with a hypersensitivity reaction to abacavir (ABC) in the STEAL Study ……….80

Protocol Synopsis

Title	A randomised, open label trial to assess the safety and efficacy of switching to fixed-dose tenofovir-emtricitabine or abacavir-lamivudine: the STEAL study.
Objectives	To compare the overall safety and efficacy of simplified dual-nucleoside reverse transcriptase inhibitor (NRTI)-based antiretroviral treatment (ART) with either once-daily abacavir (ABC)-lamivudine (3TC) fixed dose combination (FDC) or once-daily tenofovir (TDF)-emtricitabine (FTC) FDC over 2 years in HIV-infected adults currently receiving two NRTIs as part of combination ART.
Study design	The study is a randomised, open-label, 2-year trial. Three hundred and fifty (350) eligible subjects will be randomised 1:1 to switch from their existing NRTIs to either ABC 600mg-3TC 300 mg FDC or to TDF 300mg-FTC 200mg FDC.<br>Stratification will be based on;<br>1. baseline NRTIs (TDF, ABC or other)<br>2. current protease inhibitor (PI) therapy (yes or no)<br>3. site
Participant population	Eligible participants will satisfy all of the following criteria within 30 days prior to randomisation.<br><br>*Inclusion criteria*<br>1. documented HIV infection<br>2. age at least 18 years<br>3. stable (≥ 12 weeks) ART including at least two NRTIs, currently well tolerated, with no plan to change any non-nucleoside reverse transcriptase inhibitor (NNRTI) or PI component of the ART regimen at or after baseline<br>4. HIV RNA < 50 copies/mL plasma for the preceding 12 weeks<br>5. Glomerular filtration rate (GFR) ≥ 70 mL/min/1.73m^2 (estimated by the abbreviated MDRD equation) Estimated GFR = 186 x ([S_{CR}/88.4]$^{-1.154}$) x age$^{-0.203}$ x (0.742 if female) x (1.210 if African-American)<br>6. provision of written, informed consent<br><br>*Exclusion criteria*<br>1. HLA-B*5701 positive at screening OR evidence of previous ABC hypersensitivity OR clinical failure in participants taking abacavir for at least 30 days<br>2. current therapy comprising triple NRTI alone (three NRTIs with a NNRTI and/or a PI is permitted)<br>3. current or prior use of ABC/3TC FDC (Kivexa) or TDF/FTC FDC (Truvada)<br>4. history of non-traumatic osteoporotic fracture<br>5. prior hypersensitivity or intolerance to ABC, 3TC, TDF or FTC<br>6. prior clinical failure to a regimen containing ABC or TDF<br>7. prior use of TDF for control of previously active hepatitis B (HBsAg+ or HBV DNA+) in participants likely to be resistant to 3TC/FTC

	8. current therapy including unboosted atazanavir<br>9. concurrent use of aminoglycosides, IV amphotericin B, cidofovir, cisplatin, foscarnet, IV pentamidine, probenecid, adefovir or immunomodulatory agents<br>10. clinical evidence of cirrhosis (e.g. irregular liver, features of portal hypertension)<br>11. creatinine clearance < 50 mL/min (estimated by the Cockcroft-Gault equation)<br>Male: $\frac{(140 - \text{age in years}) \times (\text{wt in kg})}{0.814 \times (\text{serum creatinine in } \mu mol/L)} = CL_{Cr}$ (mL/min)<br>Female: $\frac{(140 - \text{age in years}) \times (\text{wt in kg})}{0.814 \times (\text{serum creatinine in } \mu mol/L)} \times 0.85 = CL_{Cr}$ (mL/min)
Criteria for Evaluation	The primary endpoint is virological failure defined by HIV RNA > 400 copies/mL in the absence of intercurrent illness or immunisation, on two consecutive occasions at least 1 week apart (Roche Amplicor version 1.5, LLD 50 copies/mL)<br><br>Secondary measures of interest include:<br>1. plasma HIV RNA levels<br>2. CD4+ lymphocyte count<br>3. virological resistance in those with virological failure<br>4. all serious adverse events, regardless of causality<br>5. clinical adverse events (all grades and grade 3-4), regardless of causality<br>6. use of concomitant medications for toxicity<br>7. adherence (clinician assessed)<br>8. quality of life (SF-8)<br>9. lipid parameters<br>10. glycaemic parameters<br>11. DEXA parameters (soft tissue and bone)<br>12. non-traumatic bone fracture<br>13. resolution of adverse events (e.g. dyslipidaemia, low BMD, diabetes).<br>14. progression to AIDS (Category C)<br>15. all cause mortality<br>16. modifications to antiretroviral therapy
Data analysis	Data analysis will conducted in two parts.<br><br>**PART ONE**<br><br>The comparison of rates of virological failure and secondary measures between the two randomised groups after all participants have completed 2 years of follow-up or have permanently withdrawn or been lost to follow-up. All analyses will be on an intent-to-treat basis with the aim of assessing equivalence of outcome between the randomised arms.<br><br>**PART TWO**<br><br>This analysis will compare trial outcomes between treatment groups on the basis of definition of regimen failure. Regimen failure definitions to be examined will be:<br>• virologic failure

	• composite of virologic, clinical and metabolic endpoints<br>• composite of clinical endpoints<br>• composite of metabolic endpoints<br>as defined below.<br><br>*These definitions do not mandate changes to study therapy.*<br><br><u>Virologic</u><br>HIV RNA > 400 copies/mL in the absence of intercurrent illness or immunisation, on two consecutive occasions at least 1 week apart (Roche Amplicor version 1.5, LLD 50 copies/mL)<br><u>Clinical</u><br>i. death OR<br>ii. progression to AIDS OR*<br>iii. non-fatal myocardial infarction or ischaemic stroke OR*<br>iv. treatment-related serious adverse events OR*<br>v. treatment-related grade 3-4 clinical adverse events OR*<br>vi. discontinuation of any ART component for more than 4 weeks (change in doses are permitted e.g. change from bd to qd lopinavir) OR<br>vii. hypersensitivity reactions (using ABC hypersensitivity CRF) OR*<br>*these events will be adjudicated by a blinded endpoint committee. Only events that satisfy apriori established levels of diagnostic rigour will be included in the primary analysis<br><br><u>Metabolic</u><br>i. Renal, either<br>a. GFR <60 mL/min per 1.73 m^2 on two consecutive occasions at least 48 hours apart (with patient well hydrated), using the abbreviated MDRD equation<br>b. Phosphate <0.65 mmol/L on two consecutive occasions at least 48 hours apart (with patient well hydrated)<br>OR<br>ii. Hepatic, either<br>a. Lactate > 5 mmol/L or > 2mmol/L with relevant symptoms on two consecutive occasions at least 48 hours apart (with patient well hydrated and physically rested)<br>OR<br>b. alanine aminotransferase (ALT) > 5 times the upper normal limit on two occasions at least 48 hours apart in the absence of active hepatitis A (HAV IgM+) or C (HCVRNA+)<br>OR<br><br>iii. Lipid, either<br>a. new fasting total cholesterol > 6.5 mmol/L* OR<br>b. increase in total cholesterol > 2.0 mmol/L* OR<br>c. new fasting HDL cholesterol < 0.9 mmol/L* OR

	d. reduction in HDL cholesterol > 0.5 mmol/L* OR<br>e. commencement of new lipid-lowering therapy OR<br>**on two occasions at least 4 weeks apart (after fasting for 10 hours).*<br><br>iv. Glycaemic, either<br>a. new diabetes mellitus (WHO criteria) OR<br>b. new therapy for pre-existing, worsening diabetes OR<br><br>v. Bone, either<br>a. new osteoporosis (t-score < -2.5) OR<br>b. new therapy for low BMD (e.g. bisphosphonate, PTH, calcitriol, vitamin D, but not calcium alone) OR<br>c. non-traumatic fracture of long or torso bones OR<br><br>vi. Lipodystrophy - decline in limb fat mass of > 10%
Statistical Considerations	The rates of virological failure are estimated to be 15% over 2 years. A sample size of 175 subjects per arm gives a probability of 90% to detect a two tailed 95% confidence interval of ± 15% around a given expected 0% difference between treatment arms in virologic failure rates<br><br>A DSMB analysis of safety and efficacy data will be conducted after the last participant has completed 24 weeks of follow-up. A blinded interim analysis will be performed after the last participant has completed 48 weeks of follow-up.

Study Flow Chart

**Visit**	**Screen (-4)**	**0**	**4**	**12**	**24**	**36**	**48**	**60**	**72**	**84**	**96**
**Clinical**											
Consent	X										
Medical history	X										
HIV history		X									
Dispense FDC		X	X	X	X	X	X	X	X	X	
Safety / AEs		X	X	X	X	X	X	X	X	X	X
Con meds		X	X	X	X	X	X	X	X	X	X
Adherence		X	X	X	X	X	X	X	X	X	X
Quality of life		X			X		X		X		X
Physical exam		X			X		X		X		X
Weight	X		X	X	X	X	X	X	X	X	X
Height	X										X
**Laboratory**											
HLA-B*5701[1]	X										
Biochemistry[2]	X		X	X	X	X	X	X	X	X	X
Haematology[3]		X		X	X	X	X	X	X	X	X
CD4+ count		X		X	X	X	X	X	X	X	X
HIV RNA[4]	X	X	X	X	X	X	X	X	X	X	X
Metabolics[5]	X				X		X		X		X
DEXA[6]		X					X				X
Storage[7]		X		X	X		X		X		X

[1] **HLA-B*5701 –** to be processed and shipped to a central laboratory for sequencing
[2]**Biochemistry** – electrolytes (sodium, potassium, bicarbonate, chloride, calcium), phosphate, amylase (with fractionation if abnormal), urea, creatinine, lactate, LFTs (total protein, albumin, total bilirubin, ALT, AST, alkaline phosphatase), anion gap
[3]**Haematology -** haemoglobin, white cells, neutrophils, lymphocytes, platelets
[4]**HIV RNA** – real time plasma HIV RNA analysis
[5]**Metabolics -** fasting (≥10 hours) cholesterol (total, HDL, LDL calculated) triglycerides, glucose, insulin, HOMA
[6]**DEXA -** bone and soft-tissue scan (will not be performed on pregnant participants)
[7]**Storage –** 20mL plasma, 20mL serum, viable PBMCs

1.0 Introduction

1.1 Background and Rationale

Combination antiretroviral therapy (ART) generally comprises 2 nucleoside analogue reverse transcriptase inhibitors (NRTIs) together with either one non-nucleoside analogue reverse transcriptase inhibitor (NNRTI) or one ritonavir-boosted protease inhibitor (PI). Triple NRTI therapy and therapy with two NRTIs and an unboosted PI appear less potent, whereas 3-class therapy to date is no more potent and probably more toxic.[1-4]

The optimal two NRTIs to use as an ART backbone are unknown. There are multiple potential dual-NRTI combinations available from the licensed available NRTIs, zidovudine (AZT), tenofovir (TDF), abacavir (ABC), lamivudine (3TC), emtricitabine (FTC), stavudine (d4T) and didanosine (ddI).[1-10] The most popular dual-NRTI combinations in Australia are TDF-3TC, AZT-3TC and ABC-3TC; FTC is now available as a substitute for 3TC. All the above NRTIs, except AZT (and d4T in some countries), can be administered once a day. Most dual-NRTI combinations appear to have similar virological potency, with differences in clinical effectiveness driven largely by tolerability. One key factor determining tolerability is simplicity, as simple regimens are associated with greater adherence and so greater ongoing suppression of HIV replication. To address this, AZT and 3TC were co-formulated as the fixed dose combination (FDC) pill (Combivir) in 1997, although Combivir is a twice-daily medication.

Two once-a-day dual-NRTI FDC tablets are now available for the treatment of adult HIV infection, abacavir 600mg + lamivudine 300mg (ABC-3TC) and tenofovir 300mg + emtricitabine 200mg (TDF-FTC).[11,12] Both FDCs are at least as well tolerated over 6 to 12 months as previous standard of care dual-NRTI combinations such as d4T-3TC and AZT-3TC. Both ABC and TDF appear to induce substantially less lipoatrophy than either AZT or d4T-based ART.[13-15] Data support the use of both FDCs with either an NNRTI or a boosted PI. Neither ABC-3TC nor TDF-FTC has drug interactions that prevent dosing with an NNRTI or a PI, although TDF should not be dosed with ddI (because of the risk of CD4+ lymphocyte count declines) and TDF also necessitates RTV boosting of atazanavir. In addition, TDF levels are increased by co-administered atazanavir or lopinavir, although greater toxicity has not been reported. Both FDCs are licensed in the United States, and will possibly be available

in Australia in late 2005 / early 2006. These tablets are likely to be the most widely prescribed dual NRTIs for the foreseeable future.

The available (non-randomised) data suggest that TDF-FTC and ABC-3TC may have similar antiviral potency.[5-10] TDF-FTC and ABC-3TC have not been directly compared, however, in terms of their capacity to suppress HIV replication without inducing clinically significant toxicity. Over the short term, TDF-FTC may be better tolerated, as ABC induces a hypersensitivity reaction in 8% of adults.[11] Hypersensitivity to abacavir is a multi-organ clinical syndrome usually characterised by a sign or symptom in 2 or more of the following groups: (1) fever, (2) rash, (3) gastrointestinal (including nausea, vomiting, diarrhea, or abdominal pain), (4) constitutional (including generalized malaise, fatigue, or achiness), and (5) respiratory (including dyspnea, cough, or pharyngitis). In a West Australian cohort, ABC hypersensitivity occurred in over 95% and less than 1% of HLA-B*5701-positive and negative adults, respectively.[16] HLA-B*5701 is present in about 10% of Australian Caucasians. However, in an ethnically diverse, globally-recruited cohort, HLA-B*5701 genotype was not a powerful predictor of ABC hypersensitivity.[17] These data suggest that use of ABC in Caucasian, HLA-B*5701-negative Australians adults may reduce the risk of ABC hypersensitivity in Australia. In terms of converting ABC from a twice to once-a-day medication, it is important to note that one study found that 'severe' hypersensitivity was more common with once daily ABC (5%) than with twice a day ABC (2%).[9]

TDF is associated with two relatively unique medium to long-term toxicities, namely renal tubular acidosis and loss of bone mineral density.[11] Both toxicities appear to occur infrequently over a 3-year period in adults previously naïve to ART.[5] The renal and bone effects of TDF have not been observed with ABC, although many of the detailed studies on these effects that have been undertaken with TDF have not been performed for ABC.

According to the US National Kidney Foundation, glomerular filtration rate (GFR) is the best overall indicator of kidney function, as the Cockcroft-Gault equation overestimates GFR by approximately 16%.[18,19] GFR is also an independent predictor of mortality and cardiovascular disease in HIV-uninfected adults.[18] Development of grade 3 GFR (30-59 mL/min per 1.73 m^2) requires active intervention and grade 4 GFR (15-29 mL/min per 1.73 m^2) requires preparation for dialysis or transplantation. Only 0.1% of 1298 participants receiving TDF developed grade 3 or grade 4 serum creatinine[20] (similar to that seen in the phase 3 naïve ART study of TDF[5]), whereas

10% developed a grade 3 or 4 GFR after a median 9 months exposure. Prior renal disease, abnormal baseline GFR, hypertension and concurrent nephrotoxic medications were independent predictors of grade 3 or 4 GFR. This analysis suggests that TDF might lead to clinically significant renal dysfunction over longer periods, especially in those with other recognised risk factors. No control group was presented to demonstrate the specificity of these data for TDF, and longer term outcomes are unknown.

TDF also reduces bone mineral density (BMD). The mean decline in spine bone mineral density (BMD) over 2 years of TDF therapy was 3.3%,[5] which equated to a mean decline in *t*-score of 2.2% (vs 1% for d4T), and a decline in total BMD of at least 5% in the spine or 7% in the hip in 28% (versus 21% with d4T). The proportion of subjects with normal spinal BMD and with available data that progressed to osteopenia was 8% with TDF and 5% with d4T. These decreases were associated with significant changes in 4 biochemical markers of bone metabolism (bone-specific alkaline phosphatase [bsALP], osteocalcin, serum C-telopeptide and urinary N-telopeptide) suggesting increased bone turnover. The clinical significance of these data are unclear, however, as no significant differences were observed in hip BMD measurements and there were more bone fractures (11 vs 5) observed in the d4T- vs TDF-treated participants, almost all of which were traumatic.

It is commonly assumed that 3TC and FTC have very similar potency and safety. Whether there are no differences is unknown. FTC-triphosphate has a longer intra-cellular half-life (39 hours) than 3TC-triphosphate (14 hours), suggesting a greater potential barrier to resistance in those whose dosing is suboptimal. Some antiretroviral toxicities such as dyslipidaemia and lipodystrophy, both strongly associated with some NRTIs,[21] have not been fully assessed in studies comparing 3TC to FTC, and their comparative safety and efficacy over longer periods are unknown. In addition, FTC induces a reversible skin discolouration in about 3% of adults, mainly in blacks, although this rarely results in discontinuation of FTC therapy.[11] Therefore, participants changing from 3TC to FTC should be regarded as having switched therapy and as having an altered potential adverse event profile, particularly for a switch lasting longer than 48 weeks.

Almost all ART studies measure treatment success as ongoing HIV suppression without change to assigned therapy. This approach ignores toxicities that are insidious and asymptomatic but that may nevertheless be clinically significant over the long term. Given that current standard-of-care dictates permanent therapy for

over 30 years in most participants, it has been suggested that longer term trials should include such toxicities as failures.[22] FDC-based NRTI will be highly popular with participants. Both FDCs have potential advantages and disadvantages.

1.2 Summary of information on 3TC-ABC

Lamivudine (3TC) is a synthetic nucleoside analogue, which is phosphorylated intracellularly to its active metabolite, lamivudine triphosphate. The principal mode of action of lamivudine triphosphate is inhibition of reverse transcriptase via viral DNA chain termination. Abacavir (ABC) is a carbocyclic synthetic nucleoside analogue that is converted intracellularly by cellular enzymes to the active metabolite, carbovir triphosphate, an analogue of deoxyguanosine-5-triphosphate (dGTP). Carbovir triphosphate inhibits the activity of HIV-1 reverse transcriptase both by competing with the natural substrate dGTP and by its incorporation into viral DNA resulting in chain termination.

In a single-dose, 3-way crossover bioavailability study of one 3TC-ABC FDC tablet versus two ABC (2 x 300mg) and two 3TC (2 x 150mg) tablets administered simultaneously in healthy subjects (n=25), there was no difference in the extent of absorption.

While there is extensive clinical experience with each of these drugs as separate products, data are limited for the fixed dose combination (FDC) formulation and single daily dosing of ABC (600mg)[11]. Recent data indicate that the active metabolite of ABC has an intracellular terminal half-life in excess of 20 hours. Such pharmacokinetic properties support once daily dosing. The Zodiac study compared the safety and efficacy of abacavir regimens of ART administered once versus twice daily in a randomised, placebo-controlled trial enrolling 770 treatment naïve subjects who were followed for 48 weeks.[23] Other components of the regimen were lamivudine and efavirenz. The study revealed that abacavir delivered once daily was non-inferior compared to abacavir delivered twice daily. There were no significant differences in safety variables and tolerability. In treatment naïve subjects study CNA30024 revealed the equivalence of abacavir regimens compared with AZT regimens over a 48-week period.[10]

The most common side effects with 3TC-ABC FDC are trouble sleeping, depression, headache, tiredness, dizziness, nausea, diarrhea, rash, fever, stomach pain, abnormal dreams, and anxiety. The more serious potential side effects include:

1. ABC Hypersensitivity - particular caution is urged in the diagnosis and management of ABC hypersensitivity reactions that are known to occur in approximately 5% of adult subjects treated with ABC (see section 7.3).
2. Lactic Acidosis with steatohepatitis
3. Worsening of HBV infection. Participants with HBV infection who take 3TC-ABC FDC and then stop it, may get 'flare-ups' of their hepatitis.

1.3 Summary of information on FTC-TDF

FTC is a synthetic nucleoside analog of cytidine, which is phosphorylated by cellular enzymes to form emtricitabine 5'-triphosphate. This inhibits the activity of the HIV-1 reverse transcriptase by competing with the natural substrate deoxycytidine 5'-phosphate and by being incorporated into nascent viral DNA which results in chain termination. TDF is an acyclic nucleoside phosphate diester analog of adenosine monophosphate. It requires hydrolysis for conversion to tenofovir and subsequent phosphorylations by cellular enzymes to form tenofovir diphosphate. This then inhibits the activity of HIV-1 reverse transcriptase by competing with the natural substrate deoxyadenosine 5'-triphosphate and, after incorporation into DNA, by DNA chain termination.

One FTC-TDF FDC tablet was bioequivalent to one FTC (200mg) capsule plus one TDF (300mg) tablet following a single-dose administration to fasting healthy subjects (n=39).

Safety and efficacy studies using the FDC of FTC-TDF are ongoing. Both components are cytosine analogs, have similar resistance profiles, and can each be administered once daily. Multidrug regimens in which FTC and/or TDF were compared demonstrated similar efficacy and safety (refer to inclosed product information for further details[12]).

The most common side effects include dizziness, diarrhea, nausea, vomiting, headache, rash, gas, and skin discoloration. More serious potential side effects include:

1. Lactic acidosis
2. Hepatotoxicity

3. Worsening of HBV infection
4. Renal tubular acidosis
5. Reduction of bone mineral density
6. Lipodystrophy

1.4 Research Hypothesis

ABC-3TC-based ART is clinically equivalent to TDF-FTC-based ART over 2 years when either is substituted as fixed dose combination (FDC) tablets for current NRTI therapy in HIV-infected adults with an undetectable plasma HIV viral load.

2.0 Trial Objectives

2.1 Primary objective

To assess whether the overall safety and efficacy of simplified dual-NRTI-based ART with either once-daily ABC-3TC FDC is equivalent to once daily TDF-FTC FDC over 2 years in HIV infected adults currently receiving two NRTIs as part of suppressive ART.

2.2 Secondary objectives

The secondary objectives include but are not limited to:

1. to compare the type and severity of adverse events, both clinical and laboratory (including non-traumatic bone fracture, progression to AIDS, and mortality) between the two regimens
2. to compare the overall body composition changes, including soft tissue, BMD t and z scores between the two randomised arms
3. to examine if there are any differences in the modifications to antiretroviral therapy between the two arms
4. to examine the use of concomitant therapy between the two regimens
5. to compare the resistance profile of the two fixed dose combinations to assess NRTI drug resistance profiles in virus from virologic failures
6. to examine if there is a difference in adherence as assessed by a clinician
7. to compare the quality of life between participants randomised to each of the treatment arms

3.0 Participant Population

3.1 Inclusion Criteria

1. documented HIV infection
2. age at least 18 years
3. stable (≥ 12 weeks) ART including at least two NRTIs, currently well tolerated, with no plan to change any NNRTI or PI component of the ART regimen at or after baseline
4. HIV RNA < 50 copies/mL plasma for the preceding 12 weeks
5. GFR ≥ 70 mL/min/1.73m^2 (estimated by the abbreviated MDRD equation[24] Estimated GFR = 186 x ($[S_{CR}/88.4]^{-1.154}$) x age$^{-0.203}$ x (0.742 if female) x (1.210 if African-American)
6. provision of written, informed consent

3.2 Exclusion Criteria

1. HLA-B*5701 positive at screening OR evidence of previous ABC hypersensitivity OR clinical failure in participants taking abacavir for at least 30 days
2. current therapy comprising triple NRTI therapy alone (three NRTIs with a NNRTI and/or a PI is permitted)
3. current or prior use of ABC/3TC FDC (Kivexa) or TDF/FTC FDC (Truvada)
4. history of non-traumatic osteoporotic fracture
5. prior hypersensitivity or intolerance to ABC, 3TC, TDF or FTC
6. prior clinical failure to a regimen containing ABC or TDF
7. prior use of TDF for control of previously active hepatitis B (HBsAg+ or HBV DNA+) in participants likely to be resistant to 3TC/FTC
8. current therapy including unboosted atazanavir
9. concurrent use of aminoglycosides, IV amphotericin B, cidofovir, cisplatin, foscarnet, IV pentamidine, probenecid, adefovir or immunomodulatory agents
10. Clinical evidence of cirrhosis (e.g. irregular liver, features of portal hypertension)
11. creatinine clearance < 50 mL/min (estimated by the Cockcroft-Gault equation)[18,19]

$$\text{Male: } \frac{(140 - \text{age in years}) \times (\text{wt in kg})}{0.814 \times (\text{serum creatinine in } \mu\text{mol/L})} = CL_{Cr} \text{ (mL/min)}$$

$$\text{Female: } \frac{(140 - \text{age in years}) \times (\text{wt in kg})}{0.814 \times (\text{serum creatinine in } \mu\text{mol/L})} \times 0.85 = CL_{Cr} \text{ (mL/min)}$$

3.3 Study Drug Discontinuation

Prior to the study drugs being discontinued NCHECR should be contacted and the reasons for discontinuation should be documented in the subjects medical notes.

Participants who cease study drugs will, wherever possible, continue to be followed up according to the protocol study plan. Participants may revoke consent for follow-up without jeopardising their relationship with either their doctor or the NCHCER. If a participant wishes to revoke consent then, if possible the participant should return for one final visit that includes all the assessments scheduled for the week 96 visit.

The criteria for permanent study drug discontinuation will include:

- Drug-related toxicity of sufficient severity (refer Section 5.3, Toxicity Management)
- Requirement for or use of prohibited concomitant medications (refer Section 5.2)
- At the request of the primary care provider if s/he thinks the study is no longer in the best interest of the subject
- At the discretion of the investigator, or NCHECR, or for administrative reasons

Premature Cessation of the Study

The study may be terminated prematurely for one of the following reasons:

1. DSMB review of summarised data has concluded that one or both of the study regimens are ineffective or unsafe, or that the study has attained its primary objective.
2. Sponsor Decision: for a number of reasons the sponsor may be required to close the study prior to the anticipated 2 year follow-up period.

In the instance of premature cessation of the study, all participants will be asked to complete all assessments scheduled for the final visit as soon as possible prior to study close.

4.0 Trial Design

4.1 Summary of Study Design

The study is a randomised, open-label, 2-year trial. Three hundred fifty (350) eligible subjects will be randomised 1:1 to switch from their existing NRTIs to either ABC 600mg-3TC 300 mg FDC or to TDF 300mg-FTC 200mg FDC after confirmation of eligibility. This will be open to all Australian and New Zealand *(dependent on availability of study drug)* study sites and stratification will be based on;

1. baseline NRTIs (TDF, ABC, or other)
2. current PI therapy (yes or no)
3. site

The primary endpoint is virological failure defined by HIV RNA > 400 copies/mL in the absence of intercurrent illness or immunisation, on two consecutive occasions at least 1 week apart (Roche Amplicor version 1.5, LLD 50 copies/mL)

Secondary measures of interest include:

1. plasma HIV RNA levels
2. CD4+ lymphocyte count
3. virological resistance in those with virological failure (genotype if two HIV RNA readings ≥ 2 weeks apart are both greater than 1000 copies/mL plasma)
4. all serious adverse events, regardless of causality
5. clinical adverse events (all grades and grade 3-4), regardless of causality
6. use of concomitant medications for toxicity
7. adherence (clinician assessed)
8. quality of life (SF-8)
9. lipid parameters
10. glycaemic parameters
11. biochemistry (including renal bloods)
12. DEXA parameters (soft tissue and bone)
13. non-traumatic bone facture
14. resolution of adverse events (e.g. dyslipidaemia, low BMD, diabetes)
15. progression to AIDS (Category C)
16. all cause mortality
17. modifications to antiretroviral therapy

4.2 Study Conduct

This study will be conducted in accordance with the ethical principles that have their origin in the Declaration of Helsinki and will be consistent with Good Clinical Practice (GCP) and applicable regulatory requirements.

The study will be conducted in compliance with the protocol. The protocol, amendments and the subject informed consent will receive Institutional Review Board (IRB)/Independent Ethics Committee (IEC) approval/favourable opinion prior to initiation of the study.

Freely given written informed consent must be obtained from every subject or his or her legally acceptable representative prior to clinical trial participation, including informed consent for any screening procedures conducted to establish subject eligibility for the trial.

The rights, safety and well being of the trial subjects are the most important considerations and should prevail over interests of science and society.

Study personnel involved in conducting this trial will be qualified by education, training, and experience to perform their respective task(s).

This trial will not use the services of study personnel where sanctions have been invoked or where there has been scientific misconduct or fraud (e.g., loss of medical licensure, debarment).

Systems with procedures that assure the quality of every aspect of the study will be implemented.

5.0 Treatment of Subjects

5.1 Treatment

At entry, subjects will be randomised to receive either of the following study drugs in combination with the remainder of their existing ART (having ceased the NRTI component):

Kivexa™: ABC 600mg + 3TC 300mg FDC once daily
Truvada™: FTC 200mg + TDF 300mg FDC once daily*

**If the study commences before Truvada is available as a section 100 formulation then subjects will commence on FTC and TDF as separate drugs once daily and change to the FDC once approved in Australia.*

Tablets are in bottles of 30 and should be stored below 30°C. Study treatment should be continued for 24 months, or until all study visits are completed.

- All baseline assessments including scans must be completed no more then 30 days prior to initiating study medication.
- Study medication will be dispensed at designated Pharmacy Departments. One months supply will be dispensed at week 0, two months at week 4, and then 3 months supply will be dispensed from week 12 onwards.
- Medication should be taken orally with water once daily either with or without food.
- Dosage reduction of study drug is not possible due to the single once daily dosage. Dosage reduction of other concurrent antiretroviral treatment may be as per the product information.
- Cessation of study drug may be required in subjects experiencing toxicity (refer Section 3.3).

Both study drugs will be available on PBS post-study. There will be no formal drug accountability conducted as part of this study, however adherence will be assessed by the clinician.

5.2 Treatment failure

If after randomisation the participant experiences a virologic or clinical failure genotypic analysis will be performed. Then it is at the investigators discretion what change is required to the antiretroviral regimen.

5.3 Prohibited Concomitant Medications

Aminoglycosides:	amikacin, gentamicin, tobramycin, neomycin

Antibiotics:	IV pentamidine
Antifungal agents:	IV amphotericin B
Antineoplastic agents:	cisplatin
Antiviral agents:	cidofovir, foscarnet, adefovir*
Immunomodulatory agents:	interleukin 2, interferon, thalidomide
Neuromuscular agents:	probenecid

*Adefovir may be taken during the study if the participant requires therapy for hepatitis B and is not taking TDF.

5.4 Toxicity Management

Please refer to the toxicity table in Appendix 2 for information regarding the grading of specific clinical and laboratory events. If an event is not listed in the table there is information at the start of the table to help define the grade. This section of the table is copied below.

**ESTIMATING SEVERITY GRADE**				
**PARAMETER**	**GRADE 1**	**GRADE 2**	**GRADE 3**	**GRADE 4**
Clinical adverse event NOT identified elsewhere in this DAIDS AE grading table	Symptoms causing no or minimal interference with usual social & functional activities	Symptoms causing greater than minimal interference with usual social & functional activities	Symptoms causing inability to perform usual social & functional activities	Symptoms causing inability to perform basic self-care functions OR Medical or operative intervention indicated to prevent permanent impairment, persistent disability, or death

5.4.1 Management of general toxicities

5.4.1.1Grades 1 and 2 Laboratory Abnormality or Clinical Event

Continue study drug at the discretion of the investigator

5.4.1.2 *Grade 3 Laboratory Abnormality or Clinical Event*

Grade 3 clinically significant laboratory abnormalities should be confirmed by repeat testing within three to five calendar days of receipt of results and before study drug discontinuation, unless such a delay is not consistent with good medical practice

For grade 3 clinical event, study drug may be continued if the event is considered to be unrelated to study drug.

For a grade 3 clinical event, or clinically significant laboratory abnormality confirmed by repeat testing, that is considered to be related to study drug, study drug should be withheld until the toxicity returns to ≤ grade 2.

When restarting study drug following resolution of the adverse event, the study drug should be restarted at full dose after discussion with the study monitor.

If a toxicity recurs to ≥ grade 3 following FDC re-challenge and is considered related to FDC, then FDC should be permanently discontinued and subjects managed according to local practice. Participants should remain in follow-up as specified by the protocol.

5.4.1.3 Grade 4 Laboratory Abnormality or Clinical Event

For grade 4 clinical event or clinically significant laboratory abnormality confirmed by repeat testing that is considered related to study drug, study drug should be permanently discontinued and subjects managed according to local practice. The subject should be followed as clinically indicated until the event resolves to baseline, or is otherwise explained, whichever occurs first.

Study drug may be continued without dose interruption for non-clinically significant grade 4 laboratory abnormality (e.g. grade 4 CK after strenuous exercise or triglyceride elevation that is non-fasting or that can be medically managed) or clinical event considered unrelated to study drug.

5.4.2 Management of specific toxicities

5.4.2.1 ABC hypersensitivity

Refer to section 7.3.

5.4.2.2 GFR <59 mL/min per 1.73 m^2

Repeat GFR estimation with participant well-hydrated; assess for concomitant nephrotoxic therapy (e.g. ACE inhibitor, Cox1-2 inhibitor), hypertension, diabetes, renal outflow obstruction (ultrasound); withhold TDF if no other cause found; consider review by nephrologist.

5.4.2.3 Grade 3 / 4 hypophosphataemia

Cease TDF if grade 3 with new-onset fatigue / weight loss / proteinuria 2+, or if grade 4 (both on 2 occasions) in absence of new nephrotoxic drug (e.g. NSAID, ACE inhibitor).

5.4.2.4 Hyperlactataemia

For repeat values > 5mmol/L with participant well-hydrated and adequately rested, cease all NRTIs

For values 2-5mmol/L with participant well-hydrated and adequately rested, and with symptoms of hyperlactataemia (unexplained new onset fatigue, nausea, weight loss, new peripheral neuropathy), consider NRTI cessation

5.4.2.5 ALT > 5 times ULN

Repeat ALT and exclude (where relevant) acute hepatitis A, B or C (HAV IgM, HBsAg, HBV DNA, HCV RNA, HCV antibody), hepatic steatosis (lactate) or biliary disease (upper abdominal ultrasound). Determine presence of specific symptoms of hepatitis. Withhold all ART if no other cause found.

5.4.2.6 Acute hepatitis B

If receiving TDF-FTC, refer for specialist opinion

If receiving ABC-3TC, switch to TDF-FTC or add adefovir (only if not on TDF)10 mg daily.

5.4.2.7 Cholesterol > 6.5 mmol/L or HDL cholesterol < 0.9 mmol/L

Results should be confirmed on repeat testing approximately 4 weeks later. If persistently abnormal, estimate ischaemic heart disease risk by Framingham equation and if 10-year risk estimated > 15%; consider therapy (either a switch of PI [except atazanavir] to an NNRTI and/or lipid-lowering therapy).

Address other cardiovascular risk factors e.g. hypertension, diabetes and smoking.

5.4.2.8 Diabetes

Confirm with repeat fasting plasma glucose, institute diet and exercise counselling, consider endocrinological review

Address other cardiovascular risk factors.

5.4.2.9 Significant decline in BMD (> grade 1 toxicity refer to appendix 2)

Seek endocrinological review.

5.4.2.10 Fracture (non-traumatic)

Assess BMD; consider endocrinological review.

6.0 Study Procedures

6.1 Initial Screening Period

Potentially eligible individuals can be screened within 30 days before the randomisation visit and all results from screening must be available for randomisation. All subjects should be given adequate information about the trial including the Participant Information Sheet and be given an opportunity to ask questions about the trial. Written consent for the main trial should be obtained at the screening visit before any protocol specified assessments are performed. The following evaluations will be performed within 30 days prior to randomisation:

NOTE: Subjects must be in fasting state (≥10 hours), however should be advised to take free fluids.

6.1.1 Clinical assessments

- Complete medical history to include:
    - history of osteoporotic fracture#
    - any prior hypersensitivities to ART#
    - history of renal or liver disease#
    - personal and family history of diabetes, hypertension, ischaemic heart disease, and ischaemic stroke.
    - smoking history
- intercurrent medical conditions/malignancies within the previous 4 weeks
- ethnicity and gender at birth
- measure weight and height

6.1.2 Laboratory assessments

- HLA-B*5701 genetic typing (unless proven prior tolerance to ABC for ≥ 12 weeks)#
- safety serum chemistries and liver function tests:, electrolytes (sodium, potassium, bicarbonate, chloride, calcium, phosphate), amylase (with fractionation if abnormal), urea, creatinine, lactate, LFTs (total protein, albumin, total bilirubin, ALT, AST, alkaline phosphatase), anion gap
- glomerular filtration rate will be calculated by the abbreviated MDRD equation#:
  Estimated GFR = 186 x $([S_{CR}/88.4]^{-1.154})$ x $age^{-0.203}$ x (0.742 if female) x (1.210 if African-American)
- creatinine clearance will be calculated by the Cockcroft-Gault equation#:

  Male: $\frac{(140 - \text{age in years}) \times (\text{wt in kg})}{0.814 \times (\text{serum creatinine in µmol/L})} = CL_{Cr}$ (mL/min)

  Female: $\frac{(140 - \text{age in years}) \times (\text{wt in kg})}{0.814 \times (\text{serum creatinine in µmol/L})} \times 0.85 = CL_{Cr}$ (mL/min)
- fasting lipid parameters (triglycerides and cholesterol [total, HDL, LDL calculated])
- fasting glycaemic parameters (glucose, insulin, HOMA)
- real time plasma HIV-RNA analysis#

#required for assessment of eligibility

6.2 Randomisation visit

To proceed, all subjects must have fulfilled the eligibility criteria by the results of evaluations at screening. <u>All randomisation evaluations must be completed prior to the commencement of study drug</u>.

Trial entry procedures will include:

6.2.1 Clinical assessments

- full physical examination to include cardiovascular, respiratory, central nervous, skin, gastrointestinal and haematological systems and ears, nose and throat examination
- Full HIV history to include: mode of transmission, HIV duration, antiretroviral treatment history, and current stage of HIV disease and any prior AIDS events (CDC classification – refer Appendix 1)
- symptoms reported by the subject or symptoms identified after examination will be reviewed by the clinician at each visit and recorded on the CRFs as adverse events
- updated medical history including changes or additions to diagnoses, diseases, or any change in antiretroviral drugs, concomitant medications
- complete the clinician assessed adherence question in the eCRF (i.e. participant adherent x% of the time)
- complete SF8 quality of life questionnaire

6.2.2 Laboratory assessments

- real time plasma HIV-RNA analysis
- haematology - full blood count (haemoglobin, white cells, neutrophils, lymphocytes, platelets)
- T-lymphocytes subsets (total lymphocytes and absolute CD4+)
- storage plasma (20mL), and serum (20mL) for post-hoc analysis of indices of lipid/glucose metabolism (e.g. leptin), other cardiovascular markers (e.g. fibrinogen, PAI-1), HIV-RNA analysis and futures assays of potential interest (e.g. resistin)

- viable PBMC samples for post-hoc analysis of indices of potential interest.

6.2.3 Lipodystrophy assessments

- DEXA scan (dual-energy x-ray absorptiometry) - scans will not be conducted on any pregnant or breastfeeding woman.

6.2.4 Drug Supply and Randomisation

After completion of all randomisation study visit assessments the participant will be randomised online via the web based CRF. Eligibility must be confirmed and the participant will then be automatically randomised to commence either:

1. ABC 600mg + 3TC 300mg fixed dose combination once daily OR
2. TDF 300mg + FTC 200mg fixed dose combination* once daily

*if the fixed dose combination is not available at the commencement of the study as a section 100 drug then TDF and FTC will be prescribed separately until the fixed dose is available.

The participant will cease their existing NRTIs but continue on their current PI and/or NNRTI regimen. All current antiretroviral treatment should be documented on the antiretroviral eCRF.

The treatment assignment will be stratified according to:

1. baseline NRTIs (TDF, ABC or other)
2. current PI therapy (yes or no)
3. site

The investigator should complete a prescription for the randomised regimen and the participant should fill the prescription at the associated chemist that same day or the next. Therefore the study drug should be commenced as soon as possible after randomisation.

6.3 Follow-up visits

The scheduled date of study visits will be calculated from the date of randomisation (day 0, week 0). A schedule of the dates for study visits will be automatically provided for each participant on the eCRF, once randomisation has been completed.

For study visit week 4, the actual visit can be from week 2 to week 8. The week 12 visit can be from week 8 to week 18. For study visits after week 24, the actual visit can be 6 weeks either side of the scheduled visit date. To avoid unnecessary scanning delays, clinical sites are requested to book all study scans in advance.

NOTE: Subjects must be in fasting state, but encouraged to take free fluids at weeks 24, 48, 72, and 96.

6.3.1 Clinical assessments

- targeted physical examination (symptom directed) [week 24, 48, 72 and 96]
- symptoms reported by the subject or symptoms identified after examination will be reviewed by the clinician at each visit and recorded on the CRFs as adverse events [all visits]
- updated medical history including changes or additions to diagnoses, diseases, or any change in antiretroviral drugs, concomitant medications [all visits]
- measure weight [all visits]
- measure height [week 96]
- complete the clinician assessed adherence question in the eCRF (i.e. participant adherent x% of the time) [all visits]
- complete SF8 quality of life questionnaire [week 24, 48, 72 and 96]

6.3.2 Laboratory assessments

- haematology - full blood count (haemoglobin, white cells, neutrophils, lymphocytes, platelets) [all visits, except week 4]
- safety serum chemistries and liver function tests:, electrolytes (sodium, potassium, bicarbonate, chloride, calcium, phosphate), amylase (with fractionation if abnormal), urea, creatinine, creatine phosphokinase (CPK), lactate, LFTs (total protein, albumin, total bilirubin, ALT, AST, alkaline phosphatase), anion gap [all visits]
- glomerular filtration rate will be calculated by the abbreviated MDRD equation: Estimated GFR = 186 x $([S_{CR}/88.4]^{-1.154})$ x $age^{-0.203}$ x (0.742 if female) x (1.210 if African-American) [all visits]
- creatinine clearance will be calculated by the Cockcroft-Gault equation [all visits]:

Male: $\frac{(140 - \text{age in years}) \times (\text{wt in kg})}{0.814 \times (\text{serum creatinine in } \mu\text{mol/L})} = CL_{Cr}$ (mL/min)

Female: $\frac{(140 - \text{age in years}) \times (\text{wt in kg})}{0.814 \times (\text{serum creatinine in } \mu\text{mol/L})} \times 0.85 = CL_{Cr}$ (mL/min)

- fasting lipid parameters (triglycerides and cholesterol [total, HDL, LDL calculated]) [weeks 24, 48, 72 and 96]
- fasting glycaemic parameters (glucose, insulin, HOMA) [weeks 24, 48, 72 and 96]
- T-lymphocytes subsets (total lymphocytes, percent and absolute CD4+) [all visits, except week 4]
- real time plasma HIV-RNA analysis [all visits]
- storage plasma (20mL), and serum (20mL) for post-hoc analysis of indices of lipid/glucose metabolism (e.g. leptin), other cardiovascular markers (e.g. fibrinogen, PAI-1), HIV-RNA analysis and futures assays of potential interest (e.g. resistin) [weeks 12, 24, 48, 72 and 96]
- viable PBMC samples for post-hoc analysis of indices of potential interest [weeks 12, 24, 48, 72 and 96].
- *if virological failure occurs a genotype should be performed.*

6.3.3 Lipodystrophy assessments

- DEXA scan (dual-energy x-ray absorptiometry) [weeks 48 and 96] – scans will not be conducted on any pregnant or breastfeeding woman.

General Comments

- The window period for each study visit is continuous so that NO results are collected that cannot be utilised.
- If a visit is conducted after the window period ceases for that specific visit, it will be counted as the next visit, and the previous visit should be documented as “missed”. DO NOT conduct a second visit in the same window period.

7.0 Data and Safety Monitoring Board (DSMB)

An independent DSMB will be established. Clinicians or investigators responsible for the clinical care of study subjects or representatives of Pharmaceutical Companies are not permitted to be members of the DSMB.

The DSMB will monitor the trial and will review efficacy and safety data by treatment allocation. Safety monitoring will be carried out every six months and will include laboratory data, Grade 3 and Grade 4 adverse events, viral load data, serious adverse events (SAEs) and adverse events leading to cessation of study medication. A summary of safety and efficacy end point data will be undertaken when all recruited subjects have completed 24 weeks on study.

The DSMB will develop a formal stopping rule for early evidence of regimen failure or success. They will make their recommendations to the Protocol Steering Committee who will decide whether to amend the trial. Separate procedures will be developed to assist the DSMB in these tasks. All data will be reviewed blind to treatment allocation.

8.0 Recording and Reporting Adverse Events

8.1 Recording Adverse Events

Adverse events may occur through the course of this study and within the specified follow-up period. Adverse events may also occur during the screening period prior to randomisation as a result of protocol-specified interventions. All such events will be recorded at each participant visit on the adverse event eCRF.

An adverse event is defined as "Any untoward medical occurrence in a participant or clinical investigation subject administered a pharmaceutical product and which does not necessarily have to have a causal relationship with this treatment. An adverse event (AE) can therefore be any unfavourable and unintended sign (including abnormal laboratory findings, for example), symptom, or disease temporally associated with the use of a medicinal product, whether or not considered related to the medicinal product." (ICH Good Clinical Practice).

Pre-existing or recurrent conditions that occur during the study (for example seasonal allergies, asthma or recurrent headaches) should not be considered as adverse events unless they change in frequency or severity.

The investigator or co-investigator should assess each adverse event for the following:

8.1.1 Serious Adverse Events (SAEs)

A serious adverse event is any adverse event that fulfills one of more of the following criteria:

- Results in death,
- Is life-threatening,

Note: the term life-threatening in this definition refers to an event in which the participant was at risk of death at the time of event; it does not refer to an event which hypothetically might have caused death if it were more severe.

- Requires in-participant hospitalisation or prolongation of existing hospitalisation,
- Results in persistent or significant disability/incapacity, or
- Is a congenital anomaly/birth defect.

Medical and scientific judgement should be exercised in deciding whether expedited reporting is appropriate in other situations, such as important medical events that may not be immediately life-threatening or result in death or hospitalisation, but may jeopardise the participant or may require intervention to prevent one of the outcomes listed in the definition above. These should also be considered serious.

8.1.2 Relationship to Study Drug

The investigator or co-investigator must determine the likelihood that the test drug caused the adverse event. The clinician should document this and sign and date the source document thus ensuring that medically qualified assessment of causality has been carried out.

8.2 Reporting Adverse Events

All serious adverse events should be reported to the monitor immediately by either telephone or fax, using the serious adverse event reporting form provided. The sponsor will report any serious adverse event that fulfills the criteria for expedited reporting (unexpected and drug related events) to the appropriate regulatory authorities within the required reporting timeframe.

The investigator or the NCHECR representative responsible for the original ethics submission must also report serious adverse events to the ethics committee as required by the ethics committee reporting procedures.

Any serious adverse event that is ongoing at the post-study follow-up visit must be followed until resolution or until the event stabilises (for those events that will not resolve).

8.3 Protocol-specific adverse events

8.3.1 Abacavir Hypersensitivity

8.3.1.1 Signs and Symptoms

The abacavir hypersensitivity reaction is characterised by the appearance of symptoms indicating multi-organ involvement. The majority of participants have fever and/or rash as part of the syndrome, however reactions have occurred without rash or fever. Symptoms can occur at any time during treatment with abacavir, but usually appear within the first six weeks of initiation of treatment (median time to onset 11 days). The symptoms worsen with continued therapy and can be life threatening. These symptoms usually resolve shortly after discontinuation of abacavir.

Frequently observed signs and symptoms include fever, rash, malaise or fatigue, gastrointestinal symptoms such as nausea, vomiting, diarrhoea, or abdominal pain and respiratory symptoms such as dyspnoea, sore throat, or cough. Other signs and symptoms include myalgia, arthralgia, oedema, pharyngitis, headache, paresthesia or myolysis.

Physical findings may include rash (usually maculopapular or urticarial), lymphadenopathy or mucous membrane lesions (conjunctivitis, mouth ulceration). Abnormal chest x-ray findings may also be present (predominantly infiltrates, which can be localised).

Laboratory abnormalities may include elevated liver function tests (such as hepatic transaminases), increased creatine phosphokinase or creatinine levels, and lymphopenia.

Anaphylaxis, hypotension, liver failure, renal failure, adult respiratory distress syndrome or respiratory failure may occur.

Some participants with hypersensitivity were initially thought to have respiratory disease (pneumonia, bronchitis, pharyngitis), a flu-like illness, gastroenteritis or reactions to other medications. This delay in diagnosis of hypersensitivity has resulted in abacavir being continued or re-introduced, leading to a more severe hypersensitivity reaction or death. Therefore, the diagnosis of hypersensitivity reaction should be carefully considered for participants presenting with symptoms of these diseases. If hypersensitivity reaction cannot be ruled out, no medicinal product

containing abacavir (Ziagen, Trizivir or the abacavir/lamivudine fixed dose combination) should be restarted.

Participants who develop a hypersensitivity reaction must discontinue abacavir and must never be rechallenged with any medicinal product that contains abacavir (Ziagen, Trizivir or the abacavir/lamivudine fixed dose combination). Restarting any abacavir-containing product following a hypersensitivity reaction results in a prompt return of symptoms within hours. **This recurrence of the hypersensitivity reaction may be more severe than on initial presentation, and may include life-threatening hypotension and death.**

There have been infrequent reports of hypersensitivity reactions following reintroduction of abacavir, where the interruption was preceded by a single key symptom of hypersensitivity (rash, fever, malaise/fatigue, gastrointestinal or a respiratory symptom).

On very rare occasions hypersensitivity reactions have been reported in participants who have re-started therapy, and who had no preceding symptoms of a hypersensitivity reaction.

8.3.1.2 Management of Hypersensitivity Reactions

Participants developing signs or symptoms of hypersensitivity MUST contact their doctor immediately for advice.

If a hypersensitivity reaction is diagnosed the abacavir-containing product MUST be discontinued immediately. The participant should be asked to return all unused supplies of the abacavir-containing product for disposal to prevent an accidental re-challenge.

An abacavir containing medicinal product (Ziagen, Trizivir or the abacavir/lamivudine fixed dose combination), MUST NEVER be administered following a hypersensitivity reaction, as more severe symptoms will recur within hours and may include life-threatening hypotension and death.

To avoid a delay in diagnosis and minimise the risk of a life-threatening hypersensitivity reaction, the abacavir-containing product should be permanently discontinued if hypersensitivity cannot be ruled out, even when other diagnoses are

possible (respiratory diseases, flu-like illness, gastroenteritis or reactions to other medications).

Symptomatic support for abacavir hypersensitivity may be indicated. This should include, for example, administration of intravenous fluids to participants who develop hypotension. Antihistamines or corticosteroids have been used in cases of abacavir hypersensitivity, however there are no clinical data demonstrating the benefit of these in the management of the reaction.

Laboratory and other investigations which may be useful in the evaluation and treatment of abacavir hypersensitivity include, but may not be limited to, measurement of ALT, AST, creatine phosphokinase, serum creatinine and white blood cell differential count and chest x-ray, if respiratory symptoms are present.

8.3.1.3 Special considerations following an interruption of abacavir therapy

If therapy with abacavir has been discontinued and restarting therapy is under consideration, the reason for discontinuation should be evaluated to ensure that the participant did not have symptoms of a hypersensitivity reaction. **If hypersensitivity reaction cannot be ruled out, no medicinal product containing abacavir (Ziagen, Trizivir or the abacavir/lamivudine fixed dose combination) should be restarted.**

There have been infrequent reports of hypersensitivity reaction following reintroduction of an abacavir-containing product where the interruption was preceded by a single key symptom of hypersensitivity (rash, fever, malaise/fatigue, gastrointestinal symptoms or a respiratory symptom). If a decision is made to restart any abacavir-containing product in these participants, this should be done only under direct medical supervision.

On very rare occasions hypersensitivity reactions have been reported in participants who have re-started therapy, and who had no preceding symptoms of a hypersensitivity reaction. If a decision is made to re-start an abacavir-containing product, this must be done only if medical care can be accessed readily by the participant or others.

8.3.1.4 Essential participant information

Investigators must ensure that participants are fully informed regarding the following information on the hypersensitivity reaction:

- Participants must be made aware of the possibility of a hypersensitivity reaction to abacavir that may result in a life threatening reaction or death.
- Participants developing signs or symptoms possibly linked with a hypersensitivity reaction MUST CONTACT their doctor IMMEDIATELY.
- Participants who are hypersensitive to abacavir should be reminded that they must never take any abacavir containing medicinal product (Ziagen, Trizivir or the abacavir/lamivudine fixed dose combination) again.

In order to avoid restarting the abacavir-containing product, participants who have experienced a hypersensitivity reaction should be asked to return the remaining tablets or oral solution to the pharmacy.

Participants who have stopped an abacavir-containing product for any reason, and particularly due to possible adverse reactions or illness, must be advised to contact their doctor before restarting.

Each participant should be reminded to read the Package Leaflet included in the pack. Participants should be reminded of the importance of removing the Alert Card included in the pack, and keeping it with them at all times.

<u>8.3.1.5 Reporting of Hypersensitivity Reactions</u>

All cases of potential abacavir hypersensitivity should be reported to NCHECR regardless of whether or not the reaction and/or associated signs and symptoms meet seriousness criteria (see Section 8.1.2). The Abacavir Hypersensitivity Case Report Form should be completed and faxed to NCHECR within one week of the onset of the hypersensitivity reaction.

9.0 Packaging, Labeling and Accountability of Trial Supplies

9.1 Packaging and Labelling

Packaging of ABC + 3TC and FTC + TDF will be in bottles containing 30 tablets. Subjects will be instructed to take one tablet once a day. Labelling will be consistent with the requirements of the TGA.

At the commencement of the study the participants randomised to TDF-FTC FDC may need to commence the drugs independently until the co-formulation is available as a section 100 drug.

9.2 Handling and Dispensing of Study Drugs

Drug supplies should be stored in a secure area.

ABC+3TC (FDC) should be stored between 15°C - 30°C, protected from moisture, freezing, and excessive heat.

FTC +TDF (FDC) should be stored between 15°C – 30°C, protected from moisture, freezing, and excessive heat.

Once the subject has received a treatment assignment, a 30-day supply of study drugs will be dispensed to the subject from clinical supplies stored at the local pharmacy. A 60-90 day supply of study drugs will be dispensed at the Week 4 and subsequent visits. The subject must be instructed to store all medication out of reach of children.

There will be no drug accountability conducted in this study. The pharmacy should check that the supplies are in good condition and are complete as per the shipping records.

9.3 Return and Destruction of Study Drug

There will be no need to return unused or empty bottles to the pharmacy as this study is a post-marketing study and the drug is being used within the indication for usage. Therefore there is no drug accountability being conducted.

10 Biological Samples

10.1 Blood collection

It is important that the handling of blood samples is undertaken according to local guidelines and regulations for handling infectious substances. The blood tubes required to be used for each test should be as per your local laboratory guidelines. Please check with your local laboratory as to their requirements; however it is a requirement that blood for the HIV RNA assay is NOT collected in PPT tubes.

It is preferred that the blood is collected within 14 days of the planned visit date.

All lipid and glycaemic parameters must be done in the fasting state. Subjects should consume no food or beverage for at least 10 hours prior to blood collection. Prior to blood collection, subjects must have:

- Followed a regular diet for 3 days
- Undertaken normal physical activity for 3 days but no strenuous physical activity within the previous 24 hours

When blood is taken, subjects must have been sitting for at least 5 minutes. A tourniquet may be applied, but no longer than 2 minutes.

Ensure all tubes are filled to capacity. Invert all the tubes 5-10 times after filling, DO NOT SHAKE.

All samples should be kept at ROOM temperature until delivered to the laboratory.

10.2 Labeling of Specimen Tubes

It is important that the blood tubes are labeled accurately and legibly. If your normal procedure is to write directly onto the tube then please ensure you use an indelible marker and all required sections are completed. NCHECR will provide you with specimen labels for you to use if you prefer. These labels are only to be used on the blood tubes to be sent to the laboratory, not for freezing samples.

10.3 Transportation of Samples to Pathology

It is important that during the handling and transportation of blood samples precaution is taken according to local guidelines and regulations for handling infectious substances.

You will be required to set up the procedures for transporting the blood tubes to your local laboratory. It is important that the samples arrive at the laboratory within 2-3 hours of blood collection (this time frame is determined by the chemistry, lipids and glucose).

10.4 Storage of Samples

SydPath Clinical Services will be coordinating the storage samples. Blood packs will be sent to the sites and samples will be stored in the interim at local laboratories and then sent for central storage to SydPath.

10.5 Processing of Samples

All samples will be analysed at the local laboratory. It is important that the handling of blood samples is undertaken according to local guidelines and regulations for handling infectious substances. The investigator may be contacted should the technical condition of the sample, absence of information or inconsistencies on the request form be such that the samples cannot be processed. Demographic errors (i.e. incomplete or inconsistent participant information) will be called through to the site and a faxed confirmation of correct information requested. Where such errors are noted after a report has been sent to the site, then a second corrected report will be reissued by the local laboratory.

10.6 Reporting of Results

The results will be reported to you as per standard procedure. It is important that once these results are received they are entered into the eCRF and a hardcopy of the results are kept in the medical notes. If you receive the laboratory results electronically, the electronic format is adequate for source data verification.

10.7 Plasma HIV RNA Determination

Plasma HIV RNA can be measured by using either the Roche MONITOR Amplicor assay version 1.5 with a lower limit of detection of 50 copies HIV RNA/mL or the Chiron bDNA assay. Blood should not be collected in PPT tubes for HIV RNA analysis.

11.0 Statistics

The rates of treatment failure (primary endpoint) will be compared between the 2 randomised groups after all participants have completed 2 years of follow-up or have been permanently withdrawn or lost to follow-up. All analyses will be on an intent-to-treat basis with the aim of assessing the equivalence of efficacy and safety outcomes between the randomised treatment arms.

It is estimated that rate of virologic failure will be 15% over two years[25]. Given this failure rate, a sample size of 175 subjects per arm gives a probability of 90% to detect a two tailed 95% confidence interval of +/- 15% around a given expected 0% difference between treatment arms in virologic failure rates.

Additional analyses will compare trial outcomes on the basis of several definitions of regimen failure. Regimen failure definitions to be examined will be:

- virologic failure
- composite of virologic, clinical and metabolic endpoints
- composite of clinical endpoints
- composite of metabolic endpoints.

The rates of treatment failure as a composite of virologic, clinical, and metabolic events are estimated to be 40% to 45% over 2 years. Most component event rates are extrapolations from 48-week event rates (see table 2) and so must be regarded as estimates only. To provide a probability of 80% for a two tailed 95% confidence interval of +/- 15% around a given expected 0% difference between treatment arms in failure rates would require a sample size of 175 subjects per arm.

Components of the combined endpoint that are likely to differ between the treatment arms are ABC hypersensitivity, renal endpoints and bone endpoints. Based on the estimated event rates in each arm, a sample size of 175 per arm will have between 5% and 27% power to determine the difference between arms in event rate of these endpoints to be statistically significant (two sided α=0.05). Therefore the study has limited power with which to make strong conclusions based upon observed differences between study arms/or specified events.

Table 2 Estimated rates of primary event based on week 48 clinical trial data

**Event Participant**		**ABC-3TC**		**TDF-FTC**	
		**Naive*** (%)	**Any** (%)	**Naive*** (%)	**Any** (%)
Virological	HIV RNA > 400 x 2 or >1000 x 1	12	12	12	12
Clinical	Total	18	15	14	13
	Death	<1	<1	<1	<1
	AIDS	1	1	1	1
	Discontinuation of FDC for SAE or gd3-4 AE	3	2	3	2
	Discontinue for other reasons	10	10	10	10
	Discontinuation for ABC hypersensitivity	4	2	0	0
Renal	Total	2	2	10	5
	GFR < 50 mL/min per $1.73m^2$	2	2	10	5
	Grade 3 hypophosphataemia x 2	0	0	1	1

Hepatic	Total	1	1		1	1
	Hyperlactataemia	<1	<1		<1	<1
	ALT > 5 x ULN	<1	<1		<1	<1
	Acute hepatitis B	<1	<1		<1	<1
Lipid	Total	3	3		3	3
	Fasting total cholesterol > 5.5 mmol/L	0	0		0	0
	Fasting HDL cholesterol < 0.9 mmol/L	0	0		0	0
	New lipid-lowering therapy	3	3		3	3
Glycaemic	Total	3	3		3	3
	Diabetes mellitus	3	3		3	3
	New diabetes therapy	1	1		1	1
Bone	Total	1	1		5	3
	Osteoporosis	1	1		4	2
	Commencement of osteoporosis therapy	0	0		1	1
	Fracture	<1	<1		<1	<1
Fat	Decline in lamb fat mass by > 10%	5	5		5	5
**Total**		**45**	**42**		**53**	**45**

An analysis of safety and efficacy end point data will be undertaken when all recruited subjects have completed 24 weeks on study for DSMB review. An interim analysis will be performed after the last participant has completed 48 weeks of follow-up. This interim analysis will maintain concealment of randomised treatment allocation, and will compare the virological, clinical and metabolic outcomes.

Adverse events will be assigned preferred terms and categorised into body systems according to the Medical Dictionary for Drug Regulatory Affairs (MedDRA) classification of the World Health Organisation (WHO) terminology. The proportion of patients who experienced serious adverse events and adverse events of special interest will be summarised by treatment group, and by body system, and adverse event within each body system.

12.0 Data Collection, Source Documents and Record Retention

The investigator and the institution where the trial will be conducted will permit trial-related monitoring, audits, ethics committee review and regulatory inspection providing direct access to the source documents.

The data will be collected on trial specific electronic case record forms. The investigator is responsible for ensuring that the data collected is complete, accurate and recorded in a timely manner. Following each participant visit, the investigator or designated site staff member will submit the CRF electronically to the NCHECR. The data will then be reviewed for completeness and accuracy. Any discrepancies will be notified to the investigator or site coordinator for clarification.

The investigator should also complete accurate source documentation to support the data collected on the case record form. Source documents include all recordings of observations or notations of clinical activities and all reports and records necessary for the evaluation and reconstruction of the clinical trial. Source documents include, but are not limited to, the participant medical records, laboratory reports, DEXA scans, participant progress notes, pharmacy records and any other similar reports or records of procedures performed in accordance with the protocol.

It is not acceptable for the eCRF to be the only record of the participant participation in the study and participant progress should also be recorded in the participant medical records. This is to ensure that anyone who would access the participant medical records has adequate knowledge that the participant is participating in a clinical trial.

Any document that acts as a source document (the point of the initial recording of a piece of data) should be signed and dated by the person recording or reviewing the data for issues of medical significance (for example the review of laboratory reports). Persons signing the source documents should be listed as a site staff member.

The NCHECR monitor will visit the investigator site to conduct source document verification. The number of visits will be from 3-4, one shortly after study start-up and then on an annual basis. This will depend upon the recruitment rate at each investigational site; however, the monitor will conduct a minimum of two source data verification visits during the course of the study.

The investigator is responsible for retaining all essential documents listed in the ICH Good Clinical Practice guideline. These should be organised in a comprehensive filing system that is accessible to study monitors and other relevant personnel.

In Australia, the Therapeutic Goods Administration requires that the investigator must retain all study documents for 15 years following completion of the trial. The investigator is responsible for ensuring that trial records are not accidentally destroyed and it may be necessary to clearly label participant medical records to ensure that they are not accidentally destroyed in error.

13.0 Ethics Committee/Regulatory Approval and Informed Consent

The investigator (or NCHECR on behalf of the investigator) is responsible for obtaining ethics committee approval of the protocol in compliance with the local regulatory requirements prior to entering any participant into the clinical trial. The approval must clearly identify the protocol and all documents approved by the ethics committee including version numbers of the protocol and informed consent. A copy of the approval should be sent to the sponsor of the study prior to the first participant commencing the study.

The investigator (or NCHECR on behalf of the investigator) should also obtain approval for any amendments to the protocol or informed consent during the course of the study. The investigator (or NCHECR on behalf of the investigator) must comply with all ethics committee reporting requirements for serious adverse events, annual updates and end of study reports and must agree to abide by any ethics committee conditions of approval.

The investigator must obtain documented freely-given consent from each potential subject or participant prior to any protocol-specific procedures being conducted on that participant. Consent must be documented by the subject's or participant's dated signature on a Consent Form along with the dated signature of the person conducting the consent discussion.

If the subject/participant is illiterate, an impartial witness should be present during the entire consent discussion. Once the discussion is complete the participant/subject should sign and date the informed consent, if capable. The impartial witness should also sign and date the informed consent along with the person who conducted the consent discussion.

If the subject is legally incompetent (i.e. mentally incapacitated) the written consent of a parent, guardian or legal representative of the participant must be obtained.

A copy of the signed and dated consent form should be given to the subject/participant before participation in the trial. The ethics committee must review and approve the initial and any amendment consent forms prior to use in the study. The subject/participant or the legally acceptable representative should be informed in a timely manner of any new information that becomes available during the course of

the study that may affect the subject's/participant's willingness to continue participation in the trial.

This study will be conducted in accordance with the ethical principles laid out in the Declaration of Helsinki (1996) and the National Statement on Ethical Conduct in Research Involving Humans.

14.0 Confidentiality of Data

14.1 Confidentiality of Participant Records

By signing of the protocol, the investigator agrees that the sponsor, ethics committee or regulatory authorities may consult and/or copy study documents to verify information in the case record form. By signing of the consent form the participant agrees to this process.

Participant confidentiality will be maintained at all times and no documents containing the participant's name or other identifying information will be collected by the sponsor. It may be necessary for the sponsor's representatives, the ethics committee and regulatory authority representatives to have direct access to the subject/participant's medical records. If study documents need to be photocopied during the process of verifying case record form data, the subject/participant will be identified by a unique code only; full names and other identifying information will be masked.

14.2 Confidentiality of Study Data

By signing this protocol, the investigator affirms to the sponsor that information provided to the investigator by the sponsor will be maintained in confidence and will be divulged only as necessary to the ethics committee and institution employees directly involved in the study. Both ethics committee members and employees must also understand the confidentiality requirements for any information divulged to them. The data generated by this study will be considered confidential by the investigator, except to the extent that it is included in a publication as agreed in the publication policy of this protocol.

15.0 Financing and Insurance

Investigators will be paid according to the separate financial agreement document that must be signed and dated prior to study commencement.

It is the investigators responsibility to provide details of the study budget to the ethics committee, as required by each individual ethics committee. Unless the clinical site comes under the St Vincent's Hospital ethics application, in which case NCHECR will do this on behalf of the investigator.

NCHECR will organise indemnity cover for all investigators. The standard Australian Pharmaceutical Manufacturers Association indemnity form can be accessed on the Medicines Australia website at http://www.medicinesaustralia.com.au under Information and Publications/Other Publications/February 1997.

The compensation available to participants in the event of trial related injury. The Australian Pharmaceutical Manufacturers Association compensation guidelines can be accessed on the Medicines Australia website at http://www.medicinesaustralia.com.au under Information and Publications/Other Publications/February 1997.

16.0 Quality Control and Quality Assurance

By signing of this protocol, the sponsor agrees to be responsible for implementing and maintaining quality control and quality assurance systems with written standard operating procedures to ensure that trials are conducted and data are generated, documented and reported compliance with the protocol, Good Clinical Practice standards and all applicable local laws and regulations relating to the conduct of a clinical trial.

17.0 Publication Policy

There will be two final written manuscripts arising from the STEAL Study protocol. The first manuscript will describe the results analysed as per the primary endpoint. The second manuscript will analyse the results as per the composite endpoint. In the interests of collegiality and recognising that completion of this trial will have resulted from the contribution of many people the masthead authorship for the first manuscript will be "The STEAL Study Group". The protocol steering committee (PSC) will constitute the writing committee for this primary manuscript. For the second manuscript, the writing committee will be listed as authors and completed with the

phrase 'on behalf of the STEAL Study Group'. In addition, one person from each investigational site will be listed in a separate appendix as being part of the STEAL Study Group for both papers. The PSC will determine if there is a need for other appendices in which to identify others who have contributed in a significant way to the design AND conduct AND reporting of resultant trial data. If the journal will not accept group authorship the writing committee will be listed as authors and be completed with the phrase 'on behalf of the STEAL Study Group'.

Additional manuscripts that are expected to report on the findings of any subsequent substudies should have named investigators and be completed with the phrase 'on behalf of the STEAL Study Group'. In these circumstances an appendix should contain the names of the PSC.

All proposed manuscripts should be submitted to the PSC 45 days before they are to be submitted to a journal for peer review.

Conference presentations should identify an authorship group consistent with those who have contributed to the data to be reported. All proposed conference presentations should be submitted to the PSC at least 20 days before submission of an abstract.

List of References

1. Department of Health and Human Services. Guidelines for the use of antiretroviral agents in HIV-1-infected adults and adolescents. http://www.aidsinfo.nih.gov/guidelines/adult/AA_102904.pdf (accessed March 21, 2005)
2. Yeni PG, Hammer SM, Hirsch MS, et al. Treatment for adult HIV infection: 2004 recommendations of the International AIDS Society-USA Panel. **JAMA** 2004; 292: 251-65.
3. Gulick RM, Ribaudo HJ, Shikuma CM, et al. Triple-nucleoside regimens versus efavirenz-containing regimens for the initial treatment of HIV-1 infection. **N Engl J Med** 2004; 350: 1850-61.
4. Shafer RW, Smeaton LM, Robbins GK, et al. Comparison of four-drug regimens and pairs of sequential three-drug regimens as initial therapy for HIV-1 infection. **N Engl J Med** 2003; 349: 2304-15.
5. Gallant JE, Staszewski S, Pozniak AL, et al. Efficacy and safety of tenofovir DF vs stavudine in combination therapy in antiretroviral-naive participants; a 3-year randomized trial. **JAMA** 2004, 292: 191-201.
6. Molina JM, Journot V, Morand-Joubert L, et al. Simplification therapy with once-daily emtricitabine, didanosine, and efavirenz in HIV-1-infected adults with viral suppression receiving a protease inhibitor-based regimen: a randomized trial. **J Infect Dis** 2005; 191: 830-39.
7. Saag MS, Cahn P, Raffi F, et al. Efficacy and safety of emtricitabine vs stavudine in combination therapy in antiretroviral-naive participants: a randomized trial. **JAMA** 2004; 292: 180-89.
8. Benson CA, van der Horst C, LaMarcac A, et al. A randomized study of emtricitabine and lamivudine in stably suppressed participants with HIV. **AIDS** 2004; 18: 2269-76.
9. Moyle GJ, Dejesus E, Cahn P, et al. Abacavir once or twice daily combined with once-daily lamivudine and efavirenz for the treatment of antiretroviral-naive HIV-infected adults: results of the Ziagen Once Daily in Antiretroviral Combination Study. **J Acquir Immune Defic Syndr** 2005; 38: 417-25.
10. DeJesus E, Herrera G, Teofilo E, et al. Abacavir versus zidovudine combined with lamivudine and efavirenz, for the treatment of antiretroviral-naive HIV-infected adults. **Clin Infect Dis** 2004; 39: 1038-46.
11. Truvada product label. http://www.fda.gov/cder/foi/label/2004/21752lbl.pdf
12. Epzicom product label. http://www.fda.gov/cder/foi/label/2004/21652lbl.pdf
13. Carr A, Workman C, Smith D, et al. Abacavir substitution for nucleoside analogs in participants with HIV lipoatrophy: a randomized trial. **JAMA** 2002; 288: 207-15.
14. Martin A, Smith D, Carr A, et al. Changes in lipodystrophy 2 years after switching from thymidine nucleoside analogues to abacavir. **AIDS** 2004; 18: 1029-36.
15. G Moyle, C Sabin, J Cartledge, et al. A randomised, open-label comparative trial of abacavir or tenofovir DF as replacement for a thymidine analogue in persons with lipoatrophy and suppressed HIV RNA on HAART. In: Abstracts of the 12th Conference on Retroviruses and Opportunistic Infections, Boston, USA, February, 2005 (abstract).
16. Mallal S, Nolan D, Witt C, et al. Association between presence of HLA-B*5701, HLA-DR7, and HLA-DQ3 and hypersensitivity to HIV-1 reverse-transcriptase inhibitor abacavir. **Lancet** 2002; 359: 727-32.
17. Hetherington S, Hughes AR, Mosteller M, et al. Genetic variations in HLA-B region and hypersensitivity reactions to abacavir. **Lancet** 2002; 359: 1121-22.

18. Levey AS, Bosch JP, Lewis JB. A more accurate method to estimate glomerular filtration rate from serum creatinine: a new prediction equation. **Ann Intern Med** 1999; 130: 461-70.
19. Levey AS, Coresh J, Balk E, et al. National Kidney Foundation practice guidelines for chronic kidney disease: evaluation, classification, and stratification. **Ann Intern Med** 2003; 139: 137-47.
20. Becker S, Balu R, Fusco J. Beyond serum creatinine: identification of renal insufficiency using glomerular filtration: implications for clinical research and care. In: Abstracts of the 12th Conference on Retroviruses and Opportunistic Infections, Boston, February, 2005 (abstract 819).
21. Grinspoon SK, Carr A. Cardiovascular risk and body fat abnormalities in HIV-infected adults. **N Engl J Med** 2005; 352: 48-62.
22. Carr A. Improvement of the study, analysis, and reporting of adverse events associated with antiretroviral therapy. **Lancet** 2002; 360: 81-85.
23. Gazzard B et al. Zodiac: Ziagen once daily in antiretroviral combination therapy (CNA30021). 43rd Intersciences Conference on Antimicrobial Agents and Chemotherapy. September 2003. Chicago, IL.
24. The Australian Creatinine Consensus Working Group. Chronic kidney disease and automatic reporting of estimated glomerular filtration rate: a position statement. **MJA** 2005, 183 (3): 138-141.
25. Gallant JE, Staszeswki S, Pozniak AL, et al. Efficacy and safety of tenofovir DF vs stavudine in combination therapy in antiretroviral-naive patients : a 3 year randomized trial. **JAMA** 2004; 292: 191-201.

APPENDIX 1

DEFINITIONS AND CRITERIA FOR HIV DISEASE AND AIDS EVENTS

Based on the 1993 US Centres for Disease Control and Prevention definitions (MMWR 1992; 41 [No RR-17]: 1-19) and modified for this trial.

1. CATEGORY B, NON-AIDS HIV DISEASE, "ARC"

a) Constitutional disease:
- Unexplained weight loss >10% from baseline
- Unexplained diarrhoea as 2 or more loose stools per day persisting > 1 month
- Unexplained fever, >38.5^{0}C, persisting > 14 days or more than 15 days in a 30 day period

b) Infections:
- Oral hairy leukoplakia
- Oral candidiasis
- Herpes zoster (shingles), multi-dermatomal or second of two distinct episodes
- Listeriosis
- Pelvic inflammatory disease, particularly with tubo-ovarian abscess
- Vulvovaginal candidiasis, persistent, frequent or poorly responsive to treatment
- Bacilliary angiomatosis

c) Neurological:
- HIV-related peripheral neuropathy
- HIV-related vacuolar myelopathy

d) Neoplasia:
- CIN/ cervical dysplasia- moderate or severe or carcinoma-in-situ

e) Other:
- Idiopathic thrombocytopenic purpura
- Other symptomatic conditions attributed to HIV infection or defective cell-mediated immunity
- Other symptomatic conditions complicated because of HIV infection

2. CATEGORY C, AIDS with criteria for acceptance in this trial

	PRESUMPTIVE CRITERIA	DEFINITIVE CRITERIA
CONSTITUTIONAL DISEASE		
HIV wasting syndrome	Unexplained, involuntary weight loss >10% body weight associated with persistent diarrhoea with 2 or more liquid stools/day for >30 days OR chronic weakness OR persistent fever for >30days. Should exclude other causes such as cancer, TB, MAI, cryptosporidiosis or other specific enteritis	none
INFECTIONS		
Candidiasis of bronchi, trachea or lungs	none	macroscopic appearance at bronchoscopy or autopsy, or histology or cytology/smear (not culture)
Oesophageal candidiasis	Recent onset of retrosternal pain on swallowing PLUS a clinical diagnosis of oral candidiasis or by cytology smear (not culture), PLUS clinical response to treatment	macroscopic appearance at endoscopy or autopsy, or histology or cytology/smear (not culture)

	PRESUMPTIVE CRITERIA	DEFINITIVE CRITERIA
Coccidiodomycosis, disseminated or extrapulmonary	none	histology or cytology, culture or antigen detection from affected tissue
Cryptococcosis, meningitis or extrapulmonary	none	histology or cytology/microscopy, culture or antigen detection from affected tissue
Cryptosporidiosis	none	persistent diarrhoea > 1 month, histology or microscopy
CMV retinitis	Symptomatic or asymptomatic. Typical appearance on fundoscopy of discrete patches of retinal whitening, spreading along blood vessels, associated with vasculitis, haemorrhage and necrosis, confirmed by ophthalmologist.	none
CMV end-organ disease	none	compatible symptoms, plus histology or detection of antigen from affected tissue
CMV radiculomyelitis	Leg weakness and decreased reflexes or syndrome consistent with cord lesion presenting sub-acutely over days to weeks. Myelogram shows no mass lesion. CSF shows >5 WBC with >50% polymorphs and no other pathogen or persistence of symptoms after appropriate treatment for other pathogens, OR CMV shown by PCR, antigen or culture	none
CMV meningoencephalitis	Rapid (days to 1-4 weeks) syndrome with progressive delirium, cognitive impairment +/- seizures and fever (often with other CMV disease elsewhere). CT/MRI may show periventricular abnormalities with or without contrast enhancement. CSF may be normal or show evidence of CMV	none
HSV mucocutaneous ulceration	none	persistent ulceration documented > 1 month, plus histology or culture or detection of antigen from affected tissue
HSV visceral disease e.g. bronchitis, pneumonitis, oesophagitis	none	symptoms, plus histology or culture or detection of antigen from affected tissue
HZV disease	not AIDS	not AIDS
Histoplasmosis, disseminated or extrapulmonary	none	symptoms, plus histology or culture or detection of antigen from affected tissues
Isosporiasis	none	persistent diarrhoea > 1 month, histology or microscopy
Leishmaniasis, visceral	none	symptoms, plus histology
Microsporidiosis	none	persistent diarrhoea > 1 month, histology or microscopy

	PRESUMPTIVE CRITERIA	DEFINITIVE CRITERIA
MAC, and other atypical mycobacteriosis	Symptoms of fever, fatigue, anaemia or diarrhoea, plus AFBs seen in stool, blood, body fluid or tissue but not grown on culture, and no concurrent diagnosis of TB, except pulmonary	symptoms of fever, fatigue, anaemia or diarrhoea, culture from stool, blood, body fluid or tissue, except pulmonary
Tuberculosis, pulmonary	Symptoms of fever, dyspnoea, cough, weight loss or fatigue, plus AFBs seen in sputum or lavage or lung tissue but not grown in culture, plus responds to standard TB treatment	symptoms of fever, dyspnoea, cough, weight loss or fatigue, plus culture from sputum or lavage or lung tissue
Tuberculosis, extrapulmonary	Symptoms, plus AFBs seen from affected tissue or blood but not grown in culture, concurrent diagnosis of pulmonary TB or responds to standard TB treatment	symptoms, plus culture from blood or affected tissue
PCP	Recent symptoms, plus typical CXR appearance if on PCP prophylaxis or any CXR appearance if not on prophylaxis and CD4 <200, negative bronchoscopy if already treated for PCP for > 7 days or not done, no bacterial pathogens in sputum, and responds to PCP treatment	microscopy or histology
Extrapulmonary *pneumocystis*	none	symptoms plus microscopy or histology
Recurrent bacterial pneumonia	Second pneumonic episode within 1 year, new CXR appearance, symptoms and signs, diagnosed by a doctor	second pneumonic episode with 1 year, new CXR appearance, detection of bacterial pathogen
PML (Progressive Multifocal Leukoencephalopathy)	Symptoms and brain scan consistent with PML, and no response to toxo treatment	histology
Recurrent salmonella septicaemia	none	second distinct episode, culture
Cerebral toxoplasmosis	Symptoms of focal intracranial abnormality or decreased consciousness, and brain scan consistent with lesion(s) having mass effect or enhanced by contrast, and either positive toxoplasma serology or responds to treatment clinically and by scan	histology or microscopy
Other extrapulmonary toxoplasmosis	none	symptoms plus histology or microscopy

NEOPLASMS		
KS, Kaposi's sarcoma	Typical appearance without resolution. clinicians who have seen few cases should not make presumptive diagnoses	Histology
Primary cerebral lymphoma	Symptoms consistent with lymphoma, at least one lesion with mass effect on brain scan, no response clinically and by scan to toxoplasma treatment	

	PRESUMPTIVE CRITERIA	DEFINITIVE CRITERIA
B-cell, non-Hodgkin's lymphoma	none	histology
Cervical carcinoma, invasive	none	histology, not carcinoma-in-situ
NEUROLOGICAL		
HIV encephalopathy	Cognitive or motor dysfunction interfering with usual activity, progressive over weeks or months in the absence of another condition to explain the findings, should have brain scan +/- CSF to exclude other causes. Should be grade 2 or worse in at least 2 domains by NARS (see below) excluding abnormal domains at trial entry	none
OTHER		
Indeterminate intracerebral lesion(s)	Neurological illness with evidence for an intracerebral lesion(s) by brain scan where the differential diagnosis is either cerebral toxoplasmosis, PML, cerebral lymphoma or HIV encephalopathy	none

ABBREVIATED NARS (Neuropsychiatric AIDS Rating Scale) grading for <u>HIV ENCEPHALOPATHY</u>

Adapted from: Price RW, Brew BJ. The AIDS dementia complex. J Infect Dis 1988; 158 (5): 1079-83, and

Hughes CP, Berg L, Danziger WL. A new clinical scale for the staging of dementia. Brit J Psych 1982; 140: 566-92.

NARS stage	Cognitive-Behavioural Domains					
	Orientation	Memory	Motor	Behaviour	Problem solving	Activities of daily living
0.5	fully oriented	complains of memory problems	fully ambulatory slightly slowed movements	normal	has slight mental slowing	slight impairment in business dealings
1	fully oriented, may have brief periods of "spaciness"	mild memory problems	balance, co-ordination and handwriting difficulties	more irritable, labile or apathetic, withdrawn	difficulty planning and completing work	can do simple daily tasks, may need prompting
2	some disorientation	memory moderately impaired, new learning impaired	ambulatory but may require walking aid	some impulsivity or agitated behaviour	severe impairment, poor social judgement, gets lost easily	needs assistance with ADL
3	frequent disorientation	severe memory loss, only fragments of memory remain	ambulatory with assistance	may have organic psychosis	judgement very poor	cannot live independently
4	confused and disoriented	virtually no memory	bedridden	mute and unresponsive	no problem solving ability	nearly vegetative

APPENDIX 2
GRADING OF ADVERSE EVENTS

DIVISION OF AIDS TABLE FOR GRADING THE SEVERITY OF ADULT ADVERSE EVENTS PUBLISH DATE: DECEMBER, 2004

General Instructions

Estimating Severity Grade

If the need arises to grade a clinical AE that is not identified in the DAIDS AE grading table, use the category “Estimating Severity Grade” located at the top of Page 2.

Determining Severity Grade

If the severity of an AE could fall under either one of two grades (e.g., the severity of an AE could be either Grade 2 or Grade 3), select the higher of the two grades for the AE.

Definitions

Basic Self-care Functions	Activities such as bathing, dressing, toileting, transfer/movement, continence, and feeding.
LLN	Lower limit of normal
Medical Intervention	Use of pharmacologic or biologic agent(s) for treatment of an AE.
NA	Not Applicable
Operative Intervention procedures.	Surgical OR other invasive mechanical
ULN	Upper limit of normal
Usual Social & Functional Activities	Adaptive tasks and desirable activities, such as going to work, shopping, cooking, use of transportation, pursuing a hobby, etc

CLINICAL				
**PARAMETER**	**GRADE 1 MILD**	**GRADE 2 MODERATE**	**GRADE 3 SEVERE**	**GRADE 4 POTENTIALLY LIFE-THREATENING**
**ESTIMATING SEVERITY GRADE**				
Clinical adverse event NOT identified elsewhere in this DAIDS AE grading table	Symptoms causing no or minimal interference with usual social & functional activities	Symptoms causing greater than minimal interference with usual social & functional activities	Symptoms causing inability to perform usual social & functional activities	Symptoms causing inability to perform basic self-care functions OR Medical or operative intervention indicated to prevent permanent impairment, persistent disability, or death
**SYSTEMIC**				
Acute systemic allergic reaction	Localized urticaria (wheals) with no medical intervention indicated	Localized urticaria with medical intervention indicated OR Mild angioedema with no medical intervention indicated	Generalized urticaria OR Angioedema with medical intervention indicated OR Symptomatic mild bronchospasm	Acute anaphylaxis OR Life-threatening bronchospasm OR laryngeal edema
Chills	Symptoms causing no or minimal interference with usual social & functional activities	Symptoms causing greater than minimal interference with usual social & functional activities	Symptoms causing inability to perform usual social & functional activities	NA
Fatigue<br><br>Malaise	Symptoms causing no or minimal interference with usual social & functional activities	Symptoms causing greater than minimal interference with usual social & functional activities	Symptoms causing inability to perform usual social & functional activities	Incapacitating fatigue/ malaise symptoms causing inability to perform basic self-care functions
Fever (nonaxillary)	37.7 – 38.6°C	38.7 – 39.3°C	39.4 – 40.5°C	> 40.5°C
Pain (indicate body site)<br><br>DO NOT use for pain due to injection (See Injection Site Reactions: Injection site pain)<br><br>See also Headache, Arthralgia, and Myalgia	Pain causing no or minimal interference with usual social & functional activities	Pain causing greater than minimal interference with usual social & functional activities	Pain causing inability to perform usual social & functional activities	Disabling pain causing Inability to perform basic self-care functions OR Hospitalization (other than emergency room visit) indicated

PARAMETER	GRADE 1 MILD	GRADE 2 MODERATE	GRADE 3 SEVERE	GRADE 4 POTENTIALLY LIFE-THREATENING
Unintentional weight loss	NA	5 – 9% loss in body weight from baseline	10 – 19% loss in body weight from baseline	≥ 20% loss in body weight from baseline OR Aggressive intervention indicated [e.g., tube feeding or total parenteral nutrition (TPN)]
**INFECTION**				
Infection (any other than HIV infection)	Localized, no systemic antimicrobial treatment indicated AND Symptoms causing no or minimal interference with usual social & functional activities	Systemic antimicrobial treatment indicated OR Symptoms causing greater than minimal interference with usual social & functional activities	Systemic antimicrobial treatment indicated AND Symptoms causing inability to perform usual social & functional activities OR Operative intervention (other than simple incision and drainage) indicated	Life-threatening consequences (e.g., septic shock)
**SKIN – DERMATOLOGICAL**				
Alopecia	Thinning detectable by study participant (or by caregiver for young children and disabled adults)	Thinning or patchy hair loss detectable by health care provider	Complete hair loss	NA
Cutaneous reaction – rash	Localized macular rash	Diffuse macular, maculopapular, or morbilliform rash OR Target lesions	Diffuse macular, maculopapular, or morbilliform rash with vesicles or limited number of bullae OR Superficial ulcerations of mucous membrane limited to one site	Extensive or generalized bullous lesions OR Stevens-Johnson syndrome OR Ulceration of mucous membrane involving two or more distinct mucosal sites OR Toxic epidermal necrolysis (TEN)
Hyperpigmentation	Slight or localized	Marked or generalized	NA	NA
Hypopigmentation	Slight or localized	Marked or generalized	NA	NA
Pruritis (itching – no skin lesions)<br><br>(See also Injection Site Reactions: Pruritis associated with injection)	Itching causing no or minimal interference with usual social & functional activities	Itching causing greater than minimal interference with usual social & functional activities	Itching causing inability to perform usual social & functional activities	NA

PARAMETER	GRADE 1 MILD	GRADE 2 MODERATE	GRADE 3 SEVERE	GRADE 4 POTENTIALLY LIFE-THREATENING
**CARDIOVASCULAR**				
Cardiac arrhythmia (general) (By ECG or physical exam)	Asymptomatic AND No intervention indicated	Asymptomatic AND Non-urgent medical intervention indicated	Symptomatic, non-life-threatening AND Non-urgent medical intervention indicated	Life-threatening arrhythmia OR Urgent intervention indicated
Cardiac – Ischaemia / Infarction	NA	NA	Symptomatic ischemia (stable angina) OR Testing consistent with ischemia	Unstable angina OR Acute myocardial infarction
Haemorrhage (significant acute blood loss)	NA	Symptomatic AND No transfusion indicated	Symptomatic AND Transfusion of ≤ 2 units packed RCBs (for children ≤ 10 cc/kg indicated	Life threatening hypotension OR Transfusion of > 2 units packed RCBs (for children > 10 cc/kg indicated
Hypertension (with repeat testing at same visit)	> 140 – 159 mmHg systolic OR > 90 – 99 mmHg diastolic	> 160 – 179 mmHg systolic OR > 100 – 109 mmHg diastolic	> 180 mmHg systolic OR > 110 mmHg diastolic	Life-threatening consequences (e.g., malignant hypertension) OR Hospitalization indicated (other than emergency room visit)
Hypotension	NA	Symptomatic, corrected with oral fluid replacement	Symptomatic, IV fluids indicated	Shock requiring use of vasopressors or mechanical assistance to maintain blood pressure
Pericardial effusion	Asymptomatic, small effusion requiring no intervention	Asymptomatic, moderate or larger effusion requiring no intervention	Effusion with non-life threatening physiologic consequences OR Effusion with non-urgent intervention indicated	Life-threatening consequences (e.g., tamponade) OR Urgent intervention indicated
Prolonged PR Interval	PR interval 0.21 – 0.25 sec	PR interval > 0.25 sec	Type II 2nd degree AV block OR Ventricular pause > 3.0 sec	Complete AV block
Prolonged QTc	Asymptomatic, QTc interval 0.45 – 0.47 sec OR increase interval < 0.03 sec above baseline	Asymptomatic, QTc interval 0.48 – 0.49 sec OR Increase in interval 0.03 – 0.05 sec above baseline	Asymptomatic, QTc interval ≥ 0.50 sec OR Increase in interval ≥ 0.06 sec above baseline	Life-threatening consequences, e.g. Torsade de pointes or other associated serious ventricular dysrhythmia
Thrombosis/ embolism	NA	Deep vein thrombosis AND No intervention indicated (e.g., anticoagulation. Lysis filter, invasive procedure)	Deep vein thrombosis AND Intervention indicated (e.g.; anticoagulation, lysis filter, invasive procedure)	Embolic event (e.g., pulmonary embolism, life –threatening thrombus)

PARAMETER	GRADE 1 MILD	GRADE 2 MODERATE	GRADE 3 SEVERE	GRADE 4 POTENTIALLY LIFE-THREATENING
Vasovagal episode (associated with a procedure of any kind)	Present without loss of consciousness	Present with transient loss of consciousness	NA	NA
Ventricular dysfunction (congestive heart failure)	NA	Asymptomatic diagnostic finding AND intervention indicated	New onset with symptoms or Worsening symptomatic congestive heart failure	Live threatening congestive heart failure
**GASTROINTESTINAL**				
Anorexia	Loss of appetite associated without decreased oral intake	Loss of appetite associated with decreased oral intake without significant weight loss	Loss of appetite associated with significant weight loss	Life threatening consequences OR Aggressive intervention indicated [e.g., tube feeding or total parenteral nutrition (TPN)]
Ascites	Asymptomatic	Symptomatic AND Intervention indicated (e.g., diuretics or therapeutic paracentesis)	Symptomatic despite intervention	Life – threatening consequences
Cholecystitis	NA	Symptomatic AND Medical intervention indicated	Radiologic,endoscopic , or operative intervention indicated	Life-threatening consequences (e.g., sepsis or perforation)
Constipation	NA	Persistent constipation requiring regular use of dietary modifications, laxatives, or enemas	Obstipation with manual evacuation indicated	Life-threatening consequences (e.g., obstruction)
Diarrhea	Transient or intermittent episodes of unformed stools OR Increase of ≤ 3 stools over baseline per 24-hour period	Persistent episodes of unformed to watery stools OR Increase of 4 – 6 stools over baseline per 24-hour period	Bloody diarrhea OR Increase of ≥ 7 stools per 24-hour period OR IV fluid replacement indicated	Life-threatening consequences (e.g., hypotensive shock)
Dysphagia-Odynophagia	Symptomatic but able to eat usual diet	Symptoms causing altered dietary intake without medical intervention indicated	Symptoms causing severely altered dietary intake with medical intervention indicated	Life-threatening reduction in oral intake
Mucositis/stomatitis (clinical exam) Indicate site (e.g., larynx, oral) See Genitourinary for Vulvovaginitis See also Dysphagia-Odynophagia and Proctitis	Erythema of the mucosa	Patchy pseudomembranes or ulcerations	Confluent pseudomembranes or ulcerations OR Mucosal bleeding with minor trauma	Tissue necrosis OR Diffuse spontaneous mucosal bleeding OR Life –threatening consequences (e.g., aspiration, choking)

PARAMETER	GRADE 1<br>MILD	GRADE 2<br>MODERATE	GRADE 3<br>SEVERE	GRADE 4<br>POTENTIALLY LIFE-THREATENING
Nausea	Transient (< 24 hours) or intermittent nausea with no or minimal interference with oral intake	Persistent nausea resulting in decreased oral intake for 24 – 48 hours	Persistent nausea resulting in minimal oral intake for > 48 hours OR Aggressive rehydration indicated (e.g., IV fluids)	Life-threatening consequences (e.g., hypotensive shock)
Pancreatitis	NA	Symptomatic AND Hospitalization not indicated (other than emergency room visit)	Symptomatic AND Hospitalization indicated (other than emergency room visit)	Life-threatening consequences (e.g., circulatory failure, hemorrhage, sepsis)
Proctitis (functional symptomatic)<br>Also see Mucositis/stomatitis for clinical exam	Rectal discomfort AND No intervention indicated	Symptoms causing greater than minimal interference with usual social & functional activities OR Medical intervention indicated	Symptoms causing inability to perform usual social & functional activities OR Operative intervention indicated	Life-threatening consequences (e.g., perforation)
Vomiting	Transient or intermittent vomiting with no or minimal interference with oral intake	Frequent episodes of<br>vomiting with no or mild dehydration	Persistent vomiting resulting in orthostatic hypotension OR Aggressive rehydration indicated (e.g., IV fluids)	Life-threatening consequences (e.g., hypotensive shock)
**NEUROLOGIC**				
Alteration in personality-behavior or in mood (e.g., agitation, anxiety depression, mania psychosis)	Alteration causing no or minimal interference with usual social & functional activities	Alteration causing greater than minimal interference with usual social and functional activities	Alteration causing inability to perform usual social & functional activities	Behavior potentially harmful to self or others (e.g., suicidal and homicidal ideation or attempt, acute psychosis) OR causing inability to perform basic self-care functions
Altered Mental Status<br><br>For Dementia, see Cognitive and behavioural /attentional disturbance (including dementia and attention deficit disorder)	Changes causing no or minimal interference with usual social & functional activities	Mild lethargy or somnolence causing greater than minimal interference with usual social & functional activities	Confusion, memory impairment, lethargy, or somnolence causing inability to perform usual social & functional activities	Delirium OR obtundation, OR coma
Ataxia	Asymptomatic ataxia detectable on exam OR Minimal ataxia causing no or minimal interference with usual social & functional activities	Symptomatic ataxia causing greater than minimal interference with usual social & functional activities	Symptomatic ataxia causing inability to perform usual social & functional activities	Disabling ataxia causing inability to perform basic self-care functions

PARAMETER	GRADE 1 MILD	GRADE 2 MODERATE	GRADE 3 SEVERE	GRADE 4 POTENTIALLY LIFE-THREATENING
Cognitive and behavioural/ attentional disturbance (including dementia and attention deficit disorder)	Disability causing no or minimal interference with usual social & functional activities OR Specialized resources not indicated	Disability causing greater than minimal interference with usual social & functional activities OR Specialized resources on part-time basis indicated	Disability causing inability to perform usual social & functional activities OR Specialized resources on a full-time basis indicated	Disability causing inability to perform basic self-care functions OR Institutionalization indicated
CNS ischemia (acute)	NA	NA	Transient ischemic attack	Cerebral vascular accident (CVA, stroke) with neurological deficit
Headache	Symptoms causing no or minimal interference with usual social & functional activities	Symptoms causing greater than minimal interference with usual social & functional activities	Symptoms causing inability to perform usual social & functional activities	Symptoms causing inability to perform basic self-care functions OR Hospitalization indicated (other than emergency room visit) OR Headache with significant impairment of alertness or other neurologic function
Insomnia	NA	Difficulty sleeping causing greater than minimal interference with usual social & functional activities	Difficulty sleeping causing inability to perform usual social & functional activities	Disabling insomnia causing inability to perform basic self-care functions
Neuromuscular weakness (including myopathy & neuropathy)	Asymptomatic with decreased strength on exam OR Minimal muscle weakness causing no or minimal interference with usual social & functional activities	Muscle weakness causing greater than minimal interference with usual social & functional activities	Muscle weakness causing inability to perform usual social & functional activities	Disabling muscle weakness causing inability to perform basic self-care functions OR Respiratory muscle weakness impairing ventilation
Neurosensory alteration (including paresthesia and painful neuropathy)	Asymptomatic with sensory alteration on exam or minimal paresthesia causing no or minimal interference with usual social & functional activities	Sensory alteration or paresthesia causing greater than minimal interference with usual social & functional activities	Sensory alteration or paresthesia causing inability to perform usual social & functional activities	Disabling sensory alteration or paresthesia causing inability to perform basic self-care functions
Seizure: (new onset)<br><br>See also Seizure: (known pre-existing seizure disorder)	NA	1 seizure	2 – 4 seizures	Seizures of any kind which are prolonged, repetitive (e.g., status epilepticus), or difficult to control (e.g., refractory epilepsy)

PARAMETER	GRADE 1 MILD	GRADE 2 MODERATE	GRADE 3 SEVERE	GRADE 4 POTENTIALLY LIFE-THREATENING
Seizure: (known pre-existing seizure disorder)<br><br>For worsening of existing epilepsy the grades should be based on an increase from previous level of control to any of these levels.	NA	Increased frequency of pre-existing seizures (non-repetitive) without change in seizure character OR Infrequent breakthrough seizures while on stable medication in a previously controlled seizure disorder	Change in seizure character from baseline either in duration or quality (e.g., severity or focality)	Seizures of any kind which are prolonged, repetitive (e.g., status epilepticus), or difficult o control (e.g., refractory epilepsy)
Syncope (not associated with a procedure)	NA	Present	NA	NA
Vertigo	Vertigo causing no or minimal interference with usual social & functional activities	Vertigo causing greater than minimal interference with usual social & functional activities	Vertigo causing inability to perform usual social & functional activities	Disabling vertigo causing inability to perform basic self-care functions
**RESPIRATORY**				
Bronchospasm (acute)	FEV1 or peak flow reduced to 70 – 80%	FEV1 or peak flow 50 – 69%	FEV1 or peak flow 25 – 49%	Cyanosis OR FEV1 or peak flow < 25% OR Intubation
Dyspnea or respiratory distress	Dyspnea on exertion with no or minimal interference with usual social & functional activities	Dyspnea on exertion causing greater than minimal interference with usual social & functional activities	Dyspnea at rest causing inability to perform usual social & functional activities	Respiratory failure with ventilatory support indicated
**MUSCULOSKELETAL**				
Arthralgia See also Arthritis	Joint pain causing no or minimal interference with usual social & functional activities	Joint pain causing greater than minimal interference with usual social & functional activities	Joint pain causing inability to perform usual social & functional activities	Disabling joint pain causing inability to perform basic self-care functions
Arthritis See also Arthralgia	Stiffness or joint swelling causing no or minimal interference with usual social & functional activities	Stiffness or joint swelling causing greater than minimal interference with usual social & functional activities	Stiffness or joint swelling causing inability to perform usual social & functional activities	Disabling joint stiffness or swelling causing inability to perform basic self-care functions

PARAMETER	GRADE 1<br>MILD	GRADE 2<br>MODERATE	GRADE 3<br>SEVERE	GRADE 4<br>POTENTIALLY LIFE-THREATENING
Bone Mineral Loss				
Adult ≥ 21 years	BMD t-score<br>-2.5 to -1.0	BMD t-score < -2.5	Pathological fracture (including loss of vertebral height)	Pathologic fracture causing life-threatening consequences
Adult < 21 years	BMD z-score<br>-2.5 to -1.0	BMD z-score < -2.5	Pathological fracture (including loss of vertebral height)	Pathologic fracture causing life-threatening consequences
Myalgia<br>(non-injection site)	Muscle pain causing no or minimal interference with usual social & functional activities	Muscle pain causing greater than minimal interference with usual social & functional activities	Muscle pain causing inability to perform usual social & functional activities	Disabling muscle pain causing inability to perform basic self-care functions
Osteonecrosis	NA	Asymptomatic with radiographic findings AND No operative intervention indicated	Symptomatic bone pain with radiographic findings OR Operative intervention indicated	Disabling bone pain with radiographic findings causing inability to perform basic self-care functions
**GENITOURINARY**				
Cervicitis<br>(symptoms)<br>(For use in studies evaluating topical study agents)<br>For other cervicitis see Infection: Infection (any other than HIV infection)	Symptoms causing no or minimal interference with usual social & functional activities	Symptoms causing greater than minimal interference with usual social & functional activities	Symptoms causing inability to perform usual social & functional activities	Symptoms causing inability to perform basic self-care functions
Cervicitis<br>(clinical exam)<br>(For use in studies evaluating topical study agents)<br>For other cervicitis see Infection: Infection (any other than HIV infection)	Minimal cervical abnormalities on examination (erythema, mucopurulent discharge, or friability)<br>OR Epithelial disruption < 25% of total surface	Moderate cervical abnormalities on examination (erythema, mucopurulent discharge, or friability)<br>OR Epithelial disruption of 25 – 49% total surface	Severe cervical abnormalities on examination (erythema, mucopurulent discharge, or friability) OR Epithelial disruption 50 – 75% total surface	Epithelial disruption > 75% total surface
Inter-menstrual bleeding (IMB)	Spotting observed by participant OR Minimal blood observed during clinical or colposcopic examination	Inter-menstrual bleeding not greater in duration or amount than usual menstrual cycle	Inter-menstrual bleeding greater in duration or amount than usual menstrual cycle	Hemorrhage with life-threatening hypotension OR Operative intervention indicated

PARAMETER	GRADE 1<br>MILD	GRADE 2<br>MODERATE	GRADE 3<br>SEVERE	GRADE 4<br>POTENTIALLY LIFE-THREATENING
Urinary tract obstruction (e.g., stone)	NA	Signs or symptoms of urinary tract obstruction without hydronephrosis or renal dysfunction	Signs or symptoms of urinary tract obstruction with hydronephrosis or renal dysfunction	Obstruction causing life-threatening consequences
Vulvovaginitis (symptoms)<br><br>(Use in studies evaluating topical study agents)<br><br>For other vulvovaginitis see Infection: Infection (any other than HIV infection)	Symptoms causing no or minimal interference with usual social & functional activities	Symptoms causing greater than minimal interference with usual social & functional activities	Symptoms causing inability to perform usual social & Functional activities	Symptoms causing inability to perform basic self-care functions
Vulvovaginitis (clinical exam)<br><br>(Use in studies evaluating topical study agents)<br>For other vulvovaginitis see Infection: Infection (any other than HIV infection)	Minimal vaginal abnormalities on examination OR Epithelial disruption < 25% of total surface	Moderate vaginal abnormalities on examination OR Epithelial disruption of 25 - 49% total surface	Severe vaginal abnormalities on examination OR Epithelial disruption 50 - 75% total surface	Vaginal perforation OR Epithelial disruption > 75% total surface
**OCULAR/VISUAL**				
Uveitis	Asymptomatic but detectable on exam	Symptomatic anterior uveitis OR Medical intervention indicated	Posterior or pan-uveitis OR Operative intervention indicated	Disabling visual loss in affected eye(s)
Visual changes (from baseline)	Visual changes causing no or minimal interference with usual social & functional activities	Visual changes causing greater than minimal interference with usual social & functional activities	Visual changes causing inability to perform usual social & functional activities	Disabling visual loss in affected eye(s)
**ENDOCRINE/METABOLIC**				
Abnormal fat accumulation (e.g., back of neck, breasts, abdomen)	Detectable by study participant (or by caregiver for young children and disabled adults)	Detectable on physical exam by health care provider	Disfiguring OR Obvious changes on casual visual inspection	NA
Diabetes mellitus	NA	New onset without need to initiate medication OR Modification of current medications to regain glucose control	New onset with initiation of medication indicated OR Diabetes uncontrolled despite treatment modification	Life-threatening consequences (e.g., ketoacidosis, hyperosmolar non ketotic coma)

PARAMETER	GRADE 1 MILD	GRADE 2 MODERATE	GRADE 3 SEVERE	GRADE 4 POTENTIALLY LIFE-THREATENING
Gynecomastia	Detectable by study participant or caregiver (for young children and disabled adults)	Detectable on physical exam by health care provider	Disfiguring OR Obvious on casual visual inspection	NA
Hyperthyroidism	Asymptomatic	Symptomatic causing greater than minimal interference with usual social & functional activities OR Thyroid suppression therapy indicated	Symptoms causing inability to perform usual social & functional activities OR Uncontrolled despite treatment modification	Life-threatening consequences (e.g., thyroid storm)
Hypothyroidism	Asymptomatic	Symptomatic causing greater than minimal interference with usual social & functional activities OR Thyroid replacement therapy indicated	Symptoms causing inability to perform usual social & functional activities OR Uncontrolled despite treatment modification	Life-threatening consequences (e.g., myxedema coma)
Lipoatrophy (e.g., fat loss from the face, extremities, buttocks)	Detectable by study participant (or by caregiver for young children and disabled adults)	Detectable on physical exam by health care provider	Disfiguring OR Obvious on casual visual inspection	NA

LABORATORY				
**PARAMETER**	**GRADE 1 MILD**	**GRADE 2 MODERATE**	**GRADE 3 SEVERE**	**GRADE 4 POTENTIALLY LIFE-THREATENING**
**HEMATOLOGY Standard International Units are listed in italics**				
Absolute neutrophil count (ANC)	1,000 – 1,300/mm^3 1.000 x 10^9 – 1.300 x 10^9/L	750 – 999/mm^3 0.750 x 10^9 – 0.999 x 10^9/L	500 – 749/mm^3 0.500 x 10^9 – 0.749 x 10^9/L	< 500/mm^3 < 0.500 x 10^9/L
Fibrinogen, decreased	100 – 200 mg/dL 1.00 – 2.00 g/L OR 0.75 – 0.99 x LLN	75 – 99 mg/dL 0.75 – 0.99 g/L OR 0.50 – 0.74 x LLN	50 – 74 mg/dL 0.50 – 0.74 g/L OR 0.25 – 0.49 x LLN	< 50 mg/dL < 0.50 g/L OR < 0.25 x LLN OR Associated with gross bleeding
Hemoglobin (Hgb)	8.5 – 10.0 g/dL 1.32 – 1.55 mmol/L	7.5 – 8.4 g/dL 1.16 – 1.31 mmol/L	6.50 – 7.4 g/dL 1.01 – 1.15 mmol/L	< 6.5 g/dL < 1.01 mmol/L
International Normalized Ratio of prothrombin time (INR)	1.1 – 1.5 x ULN	1.6 – 2.0 x ULN	2.1 – 3.0 x ULN	> 3.0 x ULN
Methemoglobin	5.0 – 10.0%	10.1 – 15.0%	15.1 – 20.0%	> 20.0%
Prothrombin Time (PT)	1.1 – 1.25 x ULN	1.26 – 1.50 x ULN	1.51 – 3.00 x ULN	> 3.00 x ULN
Partial Thromboplastin Time (PTT)	1.1 – 1.66 x ULN	1.67 – 2.33 x ULN	2.34 – 3.00 x ULN	> 3.00 x ULN
Platelets, decreased	100,000 – 124,999/mm^3 100.000 x 10^9 – 124.999 x 10^9/L	50,000 – 99,999/mm^3 50.000 x 10^9 – 99.999 x 10^9/L	25,000 – 49,999/mm^3 25.000 x 10^9 – 49.999 x 10^9/L	< 25,000/mm3 < 25.000 x 10^9/L
WBC, decreased	2,000 – 2,500/mm^3 2.000 x 10^9 – 2.500 x 10^9/L	1,500 – 1,999/mm^3 1.500 x 10^9 – 1.999 x 10^9/L	1,000 – 1,499/mm^3 1.000 x 10^9 – 1.499 x 10^9/L	< 1,000/mm^3 < 1.000 x 10^9/L
**CHEMISTRIES Standard International Units are listed in italics**				
Acidosis	NA	pH < normal, but ≥ 7.3	pH < 7.3 without life-threatening consequences	pH < 7.3 with life-threatening consequences
Albumin, serum, low	3.0 g/dL – < LLN 30 g/L – < LLN	2.0 – 2.9 g/dL 20 – 29 g/L	< 2.0 g/dL < 20 g/L	NA
Alkaline Phosphatase	1.25 – 2.5 x ULN†	2.6 – 5.0 x ULN†	5.1 – 10.0 x ULN†	> 10.0 x ULN†
Alkalosis	NA	pH > normal, but ≤ 7.5	pH > 7.5 without life-threatening consequences	pH > 7.5 with life-threatening consequences
ALT (SGPT)	1.25 – 2.5 x ULN	2.6 – 5.0 x ULN	5.1 – 10.0 x ULN	> 10.0 x ULN
AST (SGOT)	1.25 – 2.5 x ULN	2.6 – 5.0 x ULN	5.1 – 10.0 x ULN	> 10.0 x ULN
Bicarbonate, serum, low	16.0 mEq/L – < LLN 16.0 mmol/L – < LLN	11.0 – 15.9 mEq/L 11.0 – 15.9 mmol/L	8.0 – 10.9 mEq/L 8.0 – 10.9 mmol/L	< 8.0 mEq/L < 8.0 mmol/L

† Use age and sex appropriate values (e.g. bilirubin)

PARAMETER	GRADE 1 MILD	GRADE 2 MODERATE	GRADE 3 SEVERE	GRADE 4 POTENTIALLY LIFE-THREATENING
Bilirubin (Total)	1.1 – 1.5 x ULN	1.6 – 2.5 x ULN	2.6 – 5.0 x ULN	> 5.0 x ULN
Calcium, serum, high (corrected for albumin)	10.6 – 11.5 mg/dL<br>2.65 – 2.88 mmol/L	11.6 – 12.5 mg/dL<br>2.89 – 3.13 mmol/L	12.6 – 13.5 mg/dL<br>3.14 – 3.38 mmol/L	> 13.5 mg/dL<br>> 3.38 mmol/L
Calcium, serum, low (corrected for albumin)	7.8 – 8.4 mg/dL<br>1.95 – 2.10 mmol/L	7.0 – 7.7 mg/dL<br>1.75 – 1.94 mmol/L	6.1 – 6.9 mg/dL<br>1.53 – 1.74 mmol/L	< 6.1 mg/dL<br>< 1.53 mmol/L
Cardiac troponin I (cTnI)	NA	NA	NA	Levels consistent with myocardial infarction or unstable angina as defined by the manufacturer
Cardiac troponin T (cTnT)	NA	NA	NA	≥ 0.20 ng/mL<br>OR<br>Levels consistent with myocardial infarction or unstable angina as defined by the manufacturer
Cholesterol (fasting)	200 – 239 mg/dL<br>5.18 – 6.19 mmol/L	240 – 300 mg/dL<br>6.20 – 7.77 mmol/L	> 300 mg/dL<br>> 7.77 mmol/L	NA
Creatine Kinase	3.0 – 5.9 x ULN†	6.0 – 9.9 x ULN†	10.0 – 19.9 x ULN†	≥ 20.0 x ULN†
Creatinine	1.1 – 1.3 x ULN†	1.4 – 1.8 x ULN†	1.9 – 3.4 x ULN†	≥ 3.5 x ULN†
Glomerular Filtration Rate (GFR) - abbreviated MDRD equation	60 - 89 mL/min per 1.73m^2	30 - 59 mL/min per 1.73m^2	15 – 29 mL/min per 1.73m^2	< 15 (or dialysis) mL/min per 1.73m^2
Glucose, serum, high				
Nonfasting	116 – 160 mg/dL<br>6.44 – 8.88 mmol/L	161 – 250 mg/dL<br>8.89 –13.88mmol/L	251 – 500 mg/dL<br>13.89 – 27.75 mmol/L	> 500 mg/dL<br>> 27.75 mmol/L
Fasting	110 – 125 mg/dL<br>6.11 – 6.94 mmol/L	126 – 250 mg/dL<br>6.95 – 13.88 mmol/L	251 – 500 mg/dL<br>13.89 – 27.75 mmol/L	> 500 mg/dL<br>> 27.75 mmol/L
Glucose, serum, low	55 – 64 mg/dL<br>3.05 – 3.55 mmol/L	40 – 54 mg/dL<br>2.22 – 3.06 mmol/L	30 – 39 mg/dL<br>1.67 – 2.23 mmol/L	< 30 mg/dL<br>< 1.67 mmol/L
Lactate	< 2.0 x ULN without acidosis	≥ 2.0 x ULN without acidosis	Increased lactate with pH < 7.3 without life-threatening consequences	Increased lactate with pH < 7.3 with life-threatening consequences
LDL cholesterol (fasting)	130 – 159 mg/dL<br>3.37 – 4.12 mmol/L	160 – 190 mg/dL<br>4.13 – 4.90 mmol/L	≥ 190 mg/dL<br>≥ 4.91 mmol/L	NA
Lipase	1.1 – 1.5 x ULN	1.6 – 3.0 x ULN	3.1 – 5.0 x ULN	> 5.0 x ULN
Magnesium, serum, low	1.2 – 1.4 mEq/L<br>0.60 – 0.70 mmol/L	0.9 – 1.1 mEq/L<br>0.45 – 0.59 mmol/L	0.6 – 0.8 mEq/L<br>0.30 – 0.44 mmol/L	< 0.60 mEq/L<br>< 0.30 mmol/L
Pancreatic amylase	1.1 – 1.5 x ULN	1.6 – 2.0 x ULN	2.1 – 5.0 x ULN	> 5.0 x ULN
Phosphate, serum, low	2.5 mg/dL – < LLN<br>0.81 mmol/L – < LLN	2.0 – 2.4 mg/dL<br>0.65 – 0.80 mmol/L	1.0 – 1.9 mg/dL<br>0.32 – 0.64 mmol/L	< 1.00 mg/dL<br>< 0.32 mmol/L

† use age and sex appropriate values (e.g. bilirubin)

PARAMETER	GRADE 1<br>MILD	GRADE 2<br>MODERATE	GRADE 3<br>SEVERE	GRADE 4<br>POTENTIALLY LIFE-THREATENING
Potassium, serum, high	5.6 – 6.0 mEq/L<br>5.6 – 6.0 mmol/L	6.1 – 6.5 mEq/L<br>6.1 – 6.5 mmol/L	6.6 – 7.0 mEq/L<br>6.6 – 7.0 mmol/L	> 7.0 mEq/L<br>> 7.0 mmol/L
Potassium, serum, low	3.0 – 3.4 mEq/L<br>3.0 – 3.4 mmol/L	2.5 – 2.9 mEq/L<br>2.5 – 2.9 mmol/L	2.0 – 2.4 mEq/L<br>2.0 – 2.4 mmol/L	< 2.0 mEq/L<br>< 2.0 mmol/L
Sodium, serum, high	146 – 150 mEq/L<br>146 – 150 mmol/L	151 – 154 mEq/L<br>151 – 154 mmol/L	155 – 159 mEq/L<br>155 – 159 mmol/L	≥ 160 mEq/L<br>≥ 160 mmol/L
Sodium, serum, low	130 – 135 mEq/L<br>130 – 135 mmol/L	125 – 129 mEq/L<br>125 – 129 mmol/L	121 – 124 mEq/L<br>121 – 124 mmol/L	≤ 120 mEq/L<br>≤ 120 mmol/L
Triglycerides (fasting)	NA	500 – 750 mg/dL<br>5.65 – 8.48 mmol/L	751 – 1,200 mg/dL<br>8.49 – 13.56 mmol/L	> 1,200 mg/dL<br>> 13.56 mmol/L
Uric acid	7.5 – 10.0 mg/dL<br>0.45 – 0.59 mmol/L	10.1 – 12.0 mg/dL<br>0.60 – 0.71 mmol/L	12.1 – 15.0 mg/dL<br>0.72 – 0.89 mmol/L	> 15.0 mg/dL<br>> 0.89 mmol/L

APPENDIX 3

SAMPLE PARTICIPANT INFORMATION/CONSENT

ST VINCENT'S HOSPITAL/THE UNIVERSITY OF NEW SOUTH WALES

PATIENT INFORMATION STATEMENT AND CONSENT FORM

A randomised open label trial to assess the safety and efficacy of switching to fixed-dose tenofovir-emtricitabine or abacavir-lamivudine (STEAL)

STEAL Study

Principal Investigator: A/Prof Andrew Carr
HIV, Immunology and Infectious Diseases Unit
Level 4, Xavier Building
Darlinghurst, NSW, 2010
Telephone: + 61 2 8382-3359
Facsimile: + 61 2 8382-2090
Email: acarr@stvincents.com.au

Contact Person: Alli Humphries
NCHECR
University of New South Wales (UNSW)
Sydney
Telephone: +61 2 9385 0900
Facsimile: +61 2 9385 0910

Patient Information and Consent Form

You are invited to participate in the research study outlined below.

1. Background to Research Study

Before you decide whether or not to take part in this research study, it is important that you understand why the study is being done, the procedures and assessments you will undergo, possible risks and benefits of the study and other important study information. Please read this information carefully and, if you have further questions, please ask the study doctor or research nurse. You may also wish to discuss the study with relatives or friends and may have someone with you while this study is explained to you.

The foundation of all HIV therapy is a family of HIV drugs called nucleoside analogue reverse transcriptase inhibitors. Examples include zidovudine (AZT), stavudine (d4T), didanosine (ddI), lamivudine (3TC). Many of these drugs had many side effects and require dosing several times a day. More recently, newer formulations of these drugs, as well as additional nucleoside analogues such as abacavir, tenofovir and emtricitabine (FTC), have been developed that have less side effects and can be taken once-daily. These newer pills are easier to take correctly, and so control HIV better than older pills. The most recent change to HIV nucleoside analogues has been to combine two drugs into the one tablet which helps to reduce the number of pills taken each day.

There are two new HIV combination pills that will be available in Australia in late 2005/early 2006. These two HIV combination pills each combine two HIV reverse transcriptase inhibitor drugs into the one tablet which only needs to be taken once a day:

1. Abacavir 600mg and Lamivudine 300mg (in the tablet form known as Kivexa®)
2. Emtricitabine 200mg and Tenofovir 300mg (in the tablet form known as Truvada™)

The individual drugs in each tablet are not new and are licensed for the treatment of HIV disease. In the new, once daily, one tablet formulation the long term safety of the two drugs may differ. We therefore aim to compare these two new once daily tablets to assess their safety. To be eligible for this study you must be HIV-1 positive and currently taking two nucleoside or nucleotide reverse transcriptase inhibitor drugs as part of your anti-HIV treatment.

The National Centre in HIV Epidemiology and Clinical Research, University of New South Wales in Australia is the sponsor of this study. No drug company is involved in this study.

2. Purpose of the Study

The objective of this study is to compare the safety and effectiveness over two years of two, once daily, one tablet combinations of anti-HIV drugs for the control of HIV in patients who are currently taking two nucleoside analogues as part of their HIV treatment.

3. The Study Design

An ethics committee has examined and approved this study before any participant is allowed to enroll in the study. A total of approximately 350 subjects will participate from various approved sites in Australia and New Zealand.

If you are eligible to enter the study, you will be randomised to receive either:

1. Kivexa® (Abacavir 600mg and Lamivudine 300mg)
2. Truvada™ (Emtricitabine 200mg and Tenofovir 300mg)

*If the study commences before Truvada is available in early 2006 then you will commence on emtricitabine and tenofovir as separate drugs once daily and change to the FDC once approved in Australia.

Whether you receive Kivexa® or Truvada™ will be chosen at random by a computer and your doctor has no influence on the treatment chosen for you. You will have a 50% chance of receiving one or the other. This is an open-label study so you will be aware of which treatment you are prescribed. You will need to stop your current reverse transcriptase inhibitor treatment but continue your other anti-HIV drugs (either non-nucleoside analogs and/or protease inhibitors).

4. Clinic Visits and Procedures

If, after reading this information sheet and discussing the trial with your doctor, you decide to take part you will be asked to sign a form confirming that you agree to participate.

You will be required to attend the clinic on 11 occasions over the 96 weeks of the study. You would normally visit your doctor 9 times in 96 weeks as part of your routine care. Therefore, the study requires that you visit the clinic 2 more times than you would for standard HIV monitoring.

Initial Visit (to screen for eligibility for study)

- Medical history
- Laboratory blood tests to screen for eligibility and gain a picture of your current state of health (fasting)
- If you are not already taking abacavir an additional blood test will be taken that looks at your genetic make-up; specifically the HLA-B*5701 gene. (Research shows that people with the HLA-B*5701 gene have a much higher chance of having an allergic reaction to abacavir).
- Weight and height
- Pregnancy test if required

Randomisation Visit (on commencement of study drugs)

- Physical examination by your doctor
- Laboratory blood tests for viral load and T-Cells (non-fasting)
- Pregnancy test if required
- Questionnaire about your quality of life
- Full body DEXA scan (to be done prior to commencement of study drug)

Visits at Week 4, 12, 36, 60 and 84

- Laboratory blood tests for safety and efficacy (non-fasting)
- Pregnancy test if required
- Follow up and assessment by study nurse
- Weight

Visit at Week 24, 48, 72 and 96

- Laboratory blood tests for safety and efficacy (fasting)
- Pregnancy test if required
- Follow up and assessment by study nurse
- Weight
- Physical examination by your doctor
- Questionnaire about your quality of life
- Full body DEXA scan (week 48 and 96 only)
- Height (week 96 only)

At certain visits (Randomisation and weeks 12, 24, 48, 72 and 96) you are invited to participate in providing additional clinical samples for research studies of HIV. For this research we will take an extra (*insert the amount of blood*) mls (*and tablespoons*) of blood. The samples collected for this research will be made available to researchers in Australia through the Immunovirology Research Network (IVRN), which is an Australia-wide research program funded by the Commonwealth Department of Health and Aging. The researchers will conduct laboratory projects seeking to understand aspects of HIV and the body's defence against the virus (the immune response) with the goal of improving treatment and prevention approaches. One aspect of the research supported by the Network is to study aspects of an individual's genetic make-up, which may influence the immune system's response to HIV. For this research, only genes that are believed to be relevant to the onset or outcomes of HIV and its treatment will be studied.

To analyse the results from these studies a limited amount of information about your illness will be made available to the IVRN. This information will only be provided in a coded (de-identified) format and may include items such as: your age, sex, the mode of transmission of HIV, the duration of your infection, the viral genotype and load, T cell counts, and anti-HIV treatments you have received.

If you agree to participate in this sub-study, you have the option of discontinuing at any time without withdrawing from the main study. If you change your mind, you must contact research staff and let them know that you do not want your samples used for this additional testing. The results of these tests will be used for research purposes only. Neither you nor your doctor will be given the results from these tests

5. Interaction with Concurrent Medication

Always consult your doctor about any other medication or recreational drug that you are taking. You must tell any doctor you see during the study all the medications you are taking especially before starting any new medications. This includes any other prescribed medicines, over-the-counter medicines, herbal medicines or supplements. In addition, you must tell the study doctor or nurse before enrolling in any other clinical trials while on this study.

6. Risks of Participating in the Study

Some of the side effects of the drugs in the study are listed below. Please note that this list does not include all the side effects seen with these drugs. This list includes the more serious or common side effects. If you have questions concerning any additional side effects, please ask the study doctor or nurse at your site.

Trouble sleeping	Depression	Vomiting
Headache	Tiredness	Gas
Dizziness	Nausea	Fever
Diarrhoea	Skin Discolouration	Lipodystrophy
Worsening of Hepatitis infection	Liver Toxicity	Stomach Pain

Abnormal Dreams	Anxiety	Kidney Problems
Decrease in Bone Density	A buildup of an acid in the blood	Rash

Abacavir Hypersensitivity

The anti-HIV drug Abacavir is a component of Kivexa®, therefore patients randomised to receive Kivexa® will be taking Abacavir. About 5% to 8% of patients taking Abacavir may have a hypersensitivity reaction (a serious allergic reaction) **that can very rarely be life-threatening**.

IF YOU NOTICE A SKIN RASH OR TWO OR MORE OF THE FOLLOWING SETS OF SYMPTOMS WHILE TAKING ABACAVIR, STOP TAKING IT AND CALLYOUR DOCTOR IMMEDIATELY.

• fever
• nausea, vomiting, diarrhoea, or abdominal pain
• severe tiredness, achiness, or generally ill feeling
• sore throat, shortness of breath, or cough

If you must stop treatment with abacavir because you have had this serious reaction to abacavir, **NEVER** take abacavir again. If you take abacavir again after you have had this serious reaction, **WITHIN HOURS** you may experience life-threatening symptoms that may include lowering of your blood pressure or death. You should return all of your unused Kivexa® to your doctor or pharmacist for proper disposal.

7. Risks of Study Procedures

Risks of Blood Drawing

As part of this study, you will have your blood drawn (approximately xxmls) at each visit. This procedure is uncomfortable but rarely results in any significant problems. Side effects that have been noted with drawing blood include feeling light-headed or faint, fainting, formation of a blood clot, bruising and/or infection at the site of the needlestick.

Risks of Radiation from the DEXA scan:

One toxicity of anti-HIV therapy is lipodystrophy. The best way for assessing this is with a DEXA scan. A DEXA takes 20-30 minutes to perform and does not involve any injections or preparation. And The DEXA scan involves the use of radiation. The radiation dose each time you have the scan is about 1 microsieverts. This is a very small amount of radiation. Harmful effects of radiation have not been demonstrated at this dose level and the risk is minimal. The radiation dose in this study is comparable to that from many routine nuclear medicine and medical x-ray procedures. Everyone receives about 2000-3000 times this amount in a year from natural background radiation.

Pregnancy/Breast Feeding

There may be harmful effects to the unborn child, pregnant and breast-feeding females, which are not yet known. If you think you may be pregnant at any time during the study, tell your doctor or study staff immediately so that you can have a pregnancy test. If you do become pregnant during the study you should tell your study doctor immediately. Your study doctor will discuss the options with you at that time and will follow your progress until your baby is born. You will also be enrolled onto a pregnancy registry for either Truvada™ or Kivexa®. You should not breast feed if you are HIV-positive because of the chance of passing the HIV virus to your baby. If you are a woman who has or will have a baby, talk to your doctor about the best way to prevent transmission of HIV to your baby and how to feed your baby.

8. Benefits of Participating in the Study

The information gained from this study may be of benefit in the treatment of HIV infected patients in the future. You will receive anti-HIV drugs that have been shown

to extend life in patients with HIV, reduce the number of other infections that occur with HIV infection, and otherwise improve your quality of life. However, we cannot guarantee that you as an individual will receive any benefits from this study.

Only you can decide whether to join. This involves giving written consent. Please ask your clinic doctor or nurse questions about the trial. If you do not join, it will not affect your future medical care.

You will be told if important new information relevant to the trial becomes available which could affect your willingness to continue taking part. At the end of the study, you will be told when study results may be available and how to learn about them.

If at any time your trial doctor considers that it is in your best interest to be withdrawn from the trial he or she will explain the reasons and arrange for your care to continue.

9. Alternative Treatments/Procedures
Your study doctor will discuss all of the available treatment options open to you.

10. Compensation
If you suffer any adverse experience resulting directly from the study drug/procedure, compensation will be provided for the reasonable costs of medical treatment to the extent such costs are not covered by your medical insurance or government health schemes. Compensation will be provided in accordance with the guidelines issued by Medicines Australia.

11. Study expenses
Kivexa® (Abacavir 600mg and Lamivudine 300mg) OR Truvada™ (Emtricitabine 200mg and Tenofovir 300mg) will be provided free of charge for the duration of the study. You will be required to continue to pay for your other anti-HIV drugs.

The only specific cost in this study will be the travel costs for attending the clinic on the 11 occasions for study specific visits. It is hoped you can arrange for most of these visits to tie in with your normal 3 monthly visits with your doctor.

12. Financial Support for the Study
This study is being fully funded by the National Centre in HIV Epidemiology and Clinical Research (NCHECR).

13. Questions about the study
You may ask the doctor at the study site if you have questions about the study. He/she will do all that is possible to answer your questions and concerns as they arise.

14. Statement of Subject Rights

Your participation in this study is entirely voluntary. If you decide not to take part or to withdraw from the study at any time, you will not be penalised nor lose any benefits to which you are otherwise entitled.

Your doctor will have explained the details of this trial to you and answered any questions you may have. You should be satisfied with the information you have been given and had adequate time to consider whether you want to participate. If you decide you would like to take part in this study, you will be asked to sign a consent form. If you do not want to take part, or if you choose to withdraw from the trial at any time, you will continue to receive the best medical care offered by your doctor.

Your doctor may decide to withdraw you from the trial and continue to care for you if, for example, your condition worsens and alternative treatments are thought to be preferable, or if you experience adverse effects during participation in the study. You are encouraged to contact your doctor should you decide not to continue your participation in the study. Your doctor will explain the best way for you to discontinue your participation in the research study.

All information obtained in connection with this study will remain confidential. You will be identified by your initials and a subject number (assigned to you as part of the trial) on data collection forms and laboratory results. You will not be identified in any publication or public presentation of data from this study. Only authorised personnel from the NCHECR (the sponsor's monitors and auditors), the ethics committee and government regulatory authorities will have access to your medical records. By signing this form, you give permission that these authorised persons may access your medical records. However, your records will always be treated as strictly confidential.

If new information becomes available during the course of the study that is relevant to your willingness to continue taking part in the study you will be informed promptly.

If you would like more information about the study, do not hesitate to ask your Clinic Doctor or Study Nurse ___________________ contact number _______________________________

If you have a problem during the study and would like to talk to an independent person you may contact *(insert ethics committee or other relevant contact person and contact details)*

You will be given a copy of this form to keep.

CONSENT FORM

1. I, ... of ...., aged .... years, agree to participate in the experiment described in the patient information statement set out in the attached form.

2. I acknowledge that I have read the patient information statement, which explains why I have been selected, the aims of the experiment and the nature and the possible risks of the investigation, and the statement has been explained to me to my satisfaction.

3. Before signing this consent form, I have been given the opportunity of asking any questions relating to any possible physical and mental harm I might suffer as a result of my participation and I have received satisfactory answers.

4. I understand that I can withdraw from the experiment at any time without prejudice to my relationship with my doctor.

5. I agree that research data gathered from the results of the study may be published, provided that I cannot be identified.

6. I understand that if I have any questions relating to my participation in this research, I may contact (please insert name of contacts – investigator and/or NCHECR personnel as appropriate) who will be happy to answer them.

7. I agree to have my blood samples used for IVRN testing
   ______(Patient initials)

   I **do not** agree to have my blood samples used for IVRN testing
   ______(Patient initials)

8. I acknowledge receipt of a copy of this Consent Form and the Subject Information Statement.
   Complaints may be directed to (please insert name of ethics committee contact for complaints).

______________________________	______________________________	________
Signature of Participant	Please PRINT name	Date
______________________________	______________________________	________
Signature of Investigator(s)	Please PRINT name	Date
______________________________	______________________________	________
Signature of Witness	Please PRINT name	Date
______________________________		
Nature of Witness		

CONSENT FORM

REVOCATION OF CONSENT

I hereby wish to **WITHDRAW** my consent to participate in the research proposal described above and understand that such withdrawal **WILL NOT** jeopardise any treatment or my relationship with my doctor.

____________________________ __________________________
Signature Date

____
Please PRINT Name

____
Signature of Investigator(s)

____________________________ __________________________
Please PRINT name Date

____
Signature of Witness

____________________________ __________________________
Please PRINT name Date

____
Nature of Witness

CONSENT FORM

POST STUDY INFORMATION

To understand the long term outcomes of having participated in this trial it is important that data continues to be collected on everyone who participated in the trial, even if you withdraw before the end of the study. You are therefore asked to allow your doctor to inform the National Centre of HIV Research and Epidemiology of any serious health problems that occur to you and what medications you are taking at any time in the future. No blood samples or specific visits to your doctor will be necessary. Your confidentiality will be maintained as during the study.

As you may be aware, government health regulations require that many diseases, including AIDS, be reported to the Health Department. Individuals are only identified on the basis of a code consisting of the first two letters of their first and last names, their gender and date of birth. No names or addresses are recorded. As a result, a registry exists of all people diagnosed with AIDS in Australia. It is maintained under full protection of confidentiality by the National Centre for HIV Epidemiology and Clinical Research, Sydney. The registry holds information on the date of diagnosis of AIDS, the AIDS-defining condition(s), and the likely mode of exposure to HIV.

The Australian HIV Observational Database, is to monitor in Australia patterns of HIV disease and its treatment. This will enable us to learn more about the HIV epidemic in Australia, and better target treatment and preventive programs. This database is also maintained under full protection of confidentiality by the National Centre for HIV Epidemiology and Clinical Research, Sydney. Individuals are only identified on the basis of a code consisting of the first two letters of their first and last names, their gender and date of birth. No names or addresses are recorded.

As access is restricted, we seek your consent to use this registry or database, to find out your current health status, should we be unable to locate you through your doctor.

Refusal to sign this consent **WILL NOT** preclude your entry into the study.

I, ... of ....

...., aged .... years, agree and fully understand the additional information that is being requested to be collected about me. I understand that I may withdraw my consent for this information to be collected at any time without prejudicing my relationship with my doctor.

Signature of Participant	Please PRINT name	Date
Signature of Investigator(s)	Please PRINT name	Date
Signature of Witness	Please PRINT name	Date
Nature of Witness		

APPENDIX 4

STEAL EXTENSION - CHARACTERISATION OF PARTICIPANTS CLINICALLY DIAGNOSED WITH A HYPERSENSITIVITY REACTION TO ABACAVIR (ABC) IN THE STEAL STUDY

Table of Contents

1.0 Protocol synopsis 82
2.0 Background and rationale 83
3.0 Trial objectives 83
4.0 Patient population 84
5.0 Trial design 84
6.0 Treatment of subjects 84
7.0 Study procedures 84
8.0 Statistics 85
9.0 Data Collection, Source Documents and Record Retention 85
10.0 Ethics committee/regulatory approval and informed consent 86
11.0 Financing and insurance 86
12.0 Quality control and quality assurance 86
13.0 Publication policy 86
14.0 References 86
Attachments 87
Attachment 1 – consent form template 87
Attachment 2 – patch test instructions 95

1.0 ABC HSR Synopsis

Title	Characterisation of participants clinically diagnosed with a hypersensitivity reaction to abacavir (ABC) in the STEAL Study
Objectives	• Determine if any of the 4 participants who had a presumed abacavir hypersensitivity reaction, had immunological hypersensitivity as defined by a positive skin patch test.<br>• Describe the symptoms and clinical features of those participants with suspected hypersensitivity reaction, and correlate them against standardised diagnostic criteria.<br>• Characterise immune cell responses to abacavir. This will involve measuring interferon gamma (IFN-ɣ levels in cell cultures following stimulation with abacavir.<br>• In participants with a positive result to the patch test, further investigation of this response by performing a skin biopsy for histology testing.<br>• In participants negative for the HLA-B*5701 allele but with clinically suspected abacavir allergy, characterise associations with the allergic reaction, including other ART, concomitant medications, symptoms of other concomitant infections or diseases.
Study design	• The four participants are from the STEAL study cohort.
Patient population	The four participants in this sub-study had to meet the following criteria for entry into the STEAL study as detailed above in section 3.0 of this protocol.
Treatment options and duration	Skin patch test and blood tests for cellular studies will be performed on all 4 sub-study participants using previously published methodology[4,5]. Skin punch biopsies will be done if a patient has a positive patch test.<br>• The following case definition for abacavir hypersensitivity will be used to review each case:<br>1. Must have **at least two intensifying symptoms** of the following list<br>• Rash<br>• Fever<br>• Gastrointestinal complaints – including nausea, vomiting, diarrhoea, or abdominal pain<br>• Constitutional complaints – including generalised malaise, fatigue, or achiness<br>• Respiratory complaints - including dyspnea, cough, or pharyngitis<br>2. Symptoms must occur with 6 weeks of commencement of Kivexa<br>3. The patient must have not experienced a previous hypersensitivity reaction to abacavir if taken for >6 weeks<br>4. Resolution of symptoms within 72 hours of cessation of abacavir
Data analysis	This is a descriptive study only. No formal analysis will be conducted.

2.0 Background and Rationale

Abacavir (ABC) is a nucleoside analogue reverse transcriptase inhibitor used to treat HIV. Abacavir hypersensitivity (ABC HSR) reactions occur in 5 to 8% of participants during the first 6 weeks of treatment, and the presence of the HLA-B*5701 allele is a necessary (but not sufficient) genetic factor for the occurrence of the reaction.[1] In a recent Western Australian cohort, this allele was present in 5-6% of 1956 participants.[1] This hypersensitivity reaction is potentially severe, and has led to the death of some participants. Therefore, clinicians have heightened sensitivity to diagnosing this reaction and advising termination of abacavir therapy when participants develop symptoms consistent with the reaction within 6 weeks of initiation of abacavir therapy. However, the symptoms of the reaction are non-specific, leading to false positive clinical diagnosis. In blinded trials of regimens including abacavir, hypersensitivity reaction was reported in up to 7% of participants not receiving abacavir.[2] After initiation of screening for the HLA-B*5701 allele in Western Australia, the incidence of clinical hypersensitivity reaction fell from approximately 9% to 0%.[2] Similarly, in a French cohort of 128 participants, only one participant negative for the HLA-B*5701 allele had a clinical reaction (retrospectively not considered to be a hypersensitivity reaction).[3]

Therefore, including screening for the HLA-B*5701 allele and excluding those positive for this allele from receiving abacavir would likely lead to very low clinical diagnosis of this reaction, particularly among experienced HIV physicians.

In the STEAL study, 360 participants were randomised 1:1 to receive either abacavir-lamivudine or tenofovir-emtricitabine fixed dose combinations. All participants were screened for the HLA-B*5701 allele and excluded if positive (unless evidence of prior ABC tolerance). Of the participants who were randomised to abacavir-lamivudine FDC, four participants reported a presumed clinical ABC HSR reaction. All 4 participants were screened and reported to be HLA-B*5701 negative at trial entry.

Phillips et al. (2002)[4] have shown that an epicutaneous patch test procedure is useful to use in addition to the clinical diagnosis to define immunological ABC HSR syndrome. In addition, the procedure for conducting the patch tests are common due to the use of patch tests to diagnose T cell-mediated processes such as allergic contact dermatitis, making it an attractive and simple option for clinicians. Another suggestion by Philips (2002)[4] was that if a patient is HLA negative and has a positive patch test, this finding might help to identify a new and rare allele that might also be involved in HSR. In addition, it has been shown that IFN-γ levels were higher in ABC HSR patients compared to ABC tolerant[5]. Therefore, we propose performing the skin patch and blood tests on the four STEAL study participants with presumed ABC HSR reactions to determine if their clinical reactions were likely to be immunological reactions to ABC.

3.0 Trial Objectives

3.1 Primary objectives

Determine if any of the four participants who had a presumed abacavir hypersensitivity reaction, had immunological hypersensitivity as defined by a positive skin patch test.

3.2 Secondary objectives

- Characterise immune cell response to abacavir by measuring IFN-γ levels in cell cultures following stimulation with abacavir.

- In participants with a positive result to the patch test, further investigation of this response via histology and immunohistochemistry analysis of punch biopsy samples.
- Describe the symptoms and clinical features of those participants with suspected hypersensitivity reaction, and correlate them against standardised diagnostic criteria.
- In participants negative for the HLA-B*5701 allele but with clinically suspected abacavir allergy, characterise associations with the allergy reaction, including other ART, concomitant medications, symptoms of other concomitant infections or diseases.

4.0 Patient Population

Eligibility Criteria
The participants in the sub-study met the eligibility criteria for the main study as detailed in section 3.0 and 4 participants subsequently experienced a presumed ABC HSR reaction.

5.0 Trial Design

Summary of Study Design

This is a cross-sectional, cohort sub-study of 4 participants on the STEAL study. The main aim is to determine if any of the 4 participants who had a presumed abacavir hypersensitivity reaction, had immunological hypersensitivity as defined by a positive skin patch test and cellular characterisation of blood samples. Four STEAL participants will be included in the sub-study from different STEAL study sites.

The skin patch test involves the application of dilute, non-irritating concentrations of the drug in a vehicle to the surface of the skin. Reactions to the reagent are read after 24 and 48h of application. To confirm a positive result, typical skin changes consisting of erythema and vesicular rash limited to the patch area, and no allergic response to the control vehicle are scored (please refer to Attachment 2).

The blood tests would involve collection of 17mL blood into two vacutainers for processing as serum, plasma and PBMC. These would then undergo further cellular characterisation studies, mainly measuring IFN-γ levels.

In the event that there is a positive patch test, the subjects will be asked to consent to have two small skin punch biopsies collected. The 2 biopsies would be for histology and immunohistochemistry studies.

6.0 Treatment of Subjects

All participants in the sub-study were on at least two NRTIs and either a PI or NNRTI for 3 months when randomised, they were all randomised to cease their NNRTI component of the ART regimen and commence ABC-3TC fixed dose combination, whilst continuing on their PI or NNRTI.

7.0 Study Procedures

Please refer to attachment 2 to this appendix for the detailed procedure of the patch testing.

Patients will be seen 3 times in 3 consecutive days:

Day 0: Patch preparation
Mid upper back between shoulder blades is cleaned with warm water and hypoallergenic soaps. If skin is excessively hairy the left upper arm can also be used. Patient should avoid vigorous exercise, sweating, sun exposure or showering until after patches are removed. Two different concentrations of abacavir (1% and 10%) are loaded into the four well piece of tape along with a petrolatum control. The tape is then applied to the skin.

Day 1: Initial patch reading (24 hours after patch application)
- Corner of the patch is peeled back to make an assessment of whether a reaction has occurred or not.
- The reaction will be scored according to the table below.
- A digital photograph will be taken to document any positive or suspected positive reaction.

?	Doubtful (faint erythema only)
+	Weak positive (erythema, infiltration, possibly papules)
++	Strong positive (erythema, infiltration, papules, vesicles)
+++	Extreme positive (bullous, ulcerative)
-	Negative

Day 2: Patch reading and removal (48 hours after patch application)
- The patch is removed.
- The area of application is washed with warm water and hypoallergenic soap.
- The reaction is again scored as per the table above.
- A digital photograph will be taken to document any reaction (positive or negative)
- A blood sample will be collected consisting of one ACD (yellow top) 9mL vacutainer for plasma and PBMC storage and one gel (gold top) 8.5mL vacutainer for serum storage. These samples will be collected for further analysis of immune cell response to abacavir.

- In the event that there is a positive patch test (+, ++ or +++) for any of the patients, two small skin punch biopsies, in the size of approx. 2mm, would be taken. Due to its small size, this would not require a stitch to close the site.

8.0 Statistics

Response to patch testing and blood tests will be reported on the four included participants. Description of ABC hypersensitivity as reported during the STEAL trial and in terms of the ABC hypersensitivity case definition will be reported on the four relevant participants. No formal statistical analyses will be undertaken of this sub-study as: i) there are no appropriate comparator populations; ii) the sample size is too small to calculate a meaningful estimate of variance in response.

9.0 Data Collection, Source Documents and Record Retention

Data will be collected on a paper based CRF. The investigator is responsible for ensuring that the data collected is complete and accurate. Once a patient has completed their third visit, the investigator or designated site staff member will submit the CRF to NCHECR.

10.0 Ethics Committee/Regulatory Approval and Informed Consent

The 4 participants in this sub-study will sign an additional consent form (attachment 1 to this appendix).

11.0 Financing and Insurance

Please see the STEAL protocol version 5.0 for full details on the financial and insurance arrangements for the study. All costs associated with the sub-study including costs of reagents will be covered by NCHECR. Site investigators will be paid $150 for performing the procedure. Participants' travel and work costs will be reimbursed at $100.

12.0 Quality Control and Quality Assurance

For full details please refer to the STEAL protocol Version 5.0

13.0 Publication Policy

The manuscript for the sub-study will include those investigators that constitute the ABC HSR sub-study analysis group and will be completed with the phrase 'on behalf of the STEAL Study Group'. An appendix will contain the names of the protocol steering committee and all STEAL investigators.

14.0 References

1. Mallal S, Phillips E, ,Carosi G, et al. HLA-B*5701 screening for hypersensitivity to abacavir. N Engl J Med 2008; 358: 568-579.
2. Gulick RM, Heather JR, Shikuma CM, et al. Three- vs four-drug antiretroviral regimens for the initial treatment of HIV-1 infection: A randomized controlled trial. JAMA 2006 Aug 16; 296:769-81.
3. Zucman D, de Truchis P, ,Majerholc C, Stegman S, Caillat-Zucman S. Prospective screening for human leukocyte antigen-B*5701 avoids abacavir hypersensitivity reaction in the ethnically mixed French HIV population. J Acquir Immune Defic Syndr 2007; 45: 1-3.
4. Philips E, Sullivan J, Knowles S, Shear N. Utility of patch testing in patients with hypersensitivity syndromes associated with abacavir. AIDS 2002, Vol 16 No 16: 2223.
5. Martin AM, Almeida CA; Cameron P, Purcell AW, Nolan D, James I, *et al*.. Immune responses to abacavir in antigen-presenting cells from hypersensitive patients. AIDS 2007. 21(10):1233-1244.

Attachments

Appendix 1: Patient information and consent form template

[Use institute letterhead]

PATIENT INFORMATION STATEMENT AND CONSENT FORM

Characterisation of participants clinically diagnosed with a hypersensitivity reaction to abacavir (ABC) in the STEAL Study

[*Insert institute name*]

ABC HSR Sub-study of STEAL

Principal Investigator: [*insert name of PI and contact details*]

Patient Information and Consent Form

1. Background to Research Sub-study

You have recently participated in the STEAL study. Early in this study you were diagnosed with a presumed abacavir hypersensitivity reaction and ceased abacavir (ABC). Because you experienced this reaction, you are now being invited to participate in a skin patch test and blood test to determine if you are definitely allergic to abacavir. Please note that this consent form comes as an addition to the form you have signed before entering the STEAL study.

Before you decide whether or not to take part in this research sub-study, it is important that you understand why the sub-study is being done, the procedures and assessments you will undergo, possible risks and benefits of the sub-study and other important study information. Please read this information carefully and, if you have further questions, please ask the study doctor or research nurse. You may also wish to discuss the sub-study with relatives or friends and may have someone with you while this sub-study is explained to you.

ABC is an anti-HIV drug. This drug is used in combination with other antiretroviral drugs and has proven to be highly effective. The main side effect associated with ABC is a hypersensitivity reaction (HSR) which occurs in 5-8% of people. It means that the body is oversensitive to the drug, like an allergic reaction. Symptoms of hypersensitivity include fever, skin rash, fatigue, gastrointestinal symptoms such as nausea, vomiting, diarrhoea or abdominal pain and respiratory symptoms such as sore throat, breathlessness, or cough. The symptoms usually occur during the first six weeks of therapy and can be life threatening if left untreated.

As you know, in the STEAL study, we are comparing two HIV combination tablets to assess their safety:

1. Abacavir 600mg and Lamivudine 300mg (in the combination tablet known as Kivexa®)
2. Emtricitabine 200mg and Tenofovir 300mg (in the combination tablet known as Truvada®)

Since you experienced several of the symptoms that can occur with the ABC HSR syndrome after you started taking Kivexa®, you were advised by your physician to discontinue ABC and to switch to another drug.

At the beginning of the study you had a blood test to see if you had a specific gene (HLA-B*5701) that is known to be associated with ABC HSR; you did not have this gene. Therefore it was not expected that you would experience the ABC HSR reaction. This is why we are interested in determining if it was a true allergic reaction.

A skin patch test for ABC has been developed to diagnose true ABC HSR. The patch test is performed by applying a dilute, non-irritating concentration of ABC to the skin surface on the back, similar to other allergy tests. Your doctor will then evaluate the skin for any evidence of a rash 24 and 48 hours after the patch is applied. The patch test is a research tool to confirm the clinical diagnosis; a negative result in the patch test does not imply that it is safe to take ABC again if you have experienced a ABC HSR reaction.

You will also be asked to provide an additional blood sample (approximately 17.5mL or 1 tablespoon) for cellular investigations to further determine if you are truly allergic to ABC by measuring the levels of a specific protein (Interferon-gamma - IFN-γ) in response to ABC.

If you do have a positive reaction in the patch test (a rash appears in response to the ABC) you will be invited to provide a skin sample. Your doctor will remove a very small sample of the skin (approximately 2mm) from two sites where the reaction has occurred. This sample will be sent to the lab for further investigation. You do not have to consent to this if you do not wish, however it may provide invaluable information if you have a positive patch test result. As previously mentioned, it is unexpected that any person without the specific HLA gene for ABC HSR have a positive skin reaction. Therefore if a positive patch test is found further research may expose a rare genetic factor that has not been exposed before. If you do not have a reaction to the skin patch test you will not be asked to provide any skin biopsy samples.

The National Centre in HIV Epidemiology and Clinical Research, University of New South Wales in Australia is the sponsor of this sub-study. No drug company is involved in this sub-study.

2. Purpose of the Study

The main objective of the sub-study is to determine if any of the participants who had a presumed ABC HSR reaction, had true hypersensitivity as defined by a positive skin patch test and cellular blood studies.

3. The Study Design

An ethics committee has examined and approved this study before any participant is allowed to enroll in the study. Up to 4 subjects will participate from various approved sites in Australia.

All participants in this sub-study will undergo a skin patch and blood test to confirm true ABC HSR syndrome. You will not receive any ABC tablet again.

4. Clinic Visits and Procedures

If, after reading this information sheet and discussing the trial with your doctor, you decide to take part you will be asked to sign a form confirming that you agree to participate.

You will be required to attend the clinic on 3 separate occasions over 48 hours, as detailed below.

Initial Visit (patch preparation)

- The patch will be placed on your upper back. In the patch there will be three types of gels in separated spots: low concentration of Abacavir (1%), high concentration of Abacavir (10%), and gel with no Abacavir at all.
- Please note that you should avoid vigorous exercise, sweating, sun exposure or showering until after the patch is removed.

Day 1 Visit (24 hours after patch application)

- The doctor will peel back a part of the patch to asses whether there is a skin reaction or not, and will score it accordingly.
- The doctor will take a digital photograph of the skin under the patch to document any reaction.

Day 2 Visit (48 hours after patch application)

- The doctor will remove the patch and assess any skin reactions.
- The doctor will take a digital photograph of the skin under the patch to document any reaction.
- We will collect 17.5mL blood (approximately one tablespoon). This sample will be analysed to measure levels of immunological proteins that are related

to hypersensitivity response. These samples may also be examined for rare genetic factors that may have caused the abacavir hypersensitivity reaction if you have a positive skin patch test.

If, and only if, you have a positive reaction to the skin patch test you will also be asked to undergo the following procedure:

Skin punch biopsy:

- the site where the patch test was performed will be cleaned.
- a local anaesthetic will be injected to numb the area.
- the doctor will take a small 'punch' (in the size of 2mm) with an object that looks like a pen with a small cylindrical blade at the end from two different sites on your back where the patch test was applied
- the doctor will then place the skin sample in a solution to be transported to the lab.
- pressure will be applied to the area to stop any bleeding and then a dressing will be applied to the area. Stitches are generally not required for punch biopsies as the sample taken in this case is very small.

To analyse the results from these studies, a limited amount of information about your illness will be made available to this research group. This information will only be provided in a coded (de-identified) format and may include items such as: your age, sex, the mode of transmission of HIV, the duration of your infection, the viral genotype and load, T cell counts, and anti-HIV treatments you have received.

5. Risks of Participating in the Study

There are minimal risks associated with the sub-study, the possible adverse effects are listed below. It is presumed that you will not have a reaction to the skin patch test but if you do the reaction will be mild and localised to the area of the test.

If you have questions concerning any additional side effects, please ask the study doctor or nurse at your site.

6. Risks of Study Procedures

a. Skin patch test

**Frequency**	**Adverse events**
Common<br>More than 10 in 100	Mild itchiness, redness, hardening of the skin
Extremely uncommon<br>Less than 1 in 100	Severe itchiness, redness and hardening of the skin

b. Blood Drawing
This procedure may be uncomfortable but rarely results in any significant problems. Side effects that have been noted with drawing of blood include feeling light-headed or faint, fainting, formation of a blood clot, bruising and/or infection at the site of the needlestick

c. Risks of Digital Photography
A digital photograph will be taken of the patch test area 24 and 48 hours after the patch is applied. The photograph will be of the patch test area only, which will most probably be on the back, unless there is excessive hair in which case it will on the arm. The head and face will not be included in the photograph. The digital image

will be saved onto the NCHECR internal directory and then later stored on CD with all the other project related information for long-term archiving. The digital image will only be saved with the participant's initials and unique study ID.

d. Punch Biopsy
If you have a positive skin patch reaction and agree to further investigations, you will have a punch biopsy at the final visit. This procedure may be uncomfortable but rarely results in any significant problems. Side effects that have been noted with punch biopsies include feeling light-headed or faint, fainting, formation of a blood clot, bruising and/or infection at the site of the biopsy.

7. Benefits of Participating in the Study

The information gained from this sub-study may be of benefit in the treatment of HIV infected patients in the future. However, we cannot guarantee that you as an individual will receive any benefits from this sub-study.

Only you can decide whether to join. This involves giving written consent. Please ask your clinic doctor or nurse questions about the trial. If you do not join, it will not affect your future medical care.

You will be told if important new information relevant to the trial becomes available which could affect your willingness to continue taking part. At the end of the sub-study, you will be told when study results may be available and how to learn about them.

If at any time your trial doctor considers that it is in your best interest to be withdrawn from the trial he or she will explain the reasons and arrange for your care to continue.

8. Compensation

Should any health problem occur as a result of participating in this research sub-study, the NCHECR has undertaken to abide by the Medicines Australia Clinical Trial Compensation Guidelines. These guidelines set out the circumstances under which compensation is to be provided. A copy of the guidelines covering compensation for a possible injury/disablement that occurs as a result of your participation in this study can be obtained from your doctor or the hospital ethics committee. Other forms of compensation may also be available, however you may need to seek legal assistance in order to obtain compensation. If you think that some form of compensation is required we encourage you to discuss your case with the study doctor or the hospital ethics committee.

9. Sub-study expenses

NCHECR will pay for the cost of the skin patch test kits used in the sub-study. Due to the requirement that you attend the clinic 3 days in a row we will reimburse you for your time off work and travel costs to the value of $100. This can either be paid directly to you or paid to the clinic and then paid onwards to you, which will maintain your confidentiality.

10. Financial Support for the Sub-study

Financial support to cover the costs of carrying out this sub-study are being provided by the NCHECR as part of the University of New South Wales. These funds are placed in a nominated account of the Hospital and the expenditure of the funds is in accordance with the approval given by the Human Research Ethics Committee. The account is subject to audit by the Auditors to St Vincent's Hospital.

11. Questions about the sub-study

You may ask the doctor at the study site if you have questions about the sub-study. He/she will do all that is possible to answer your questions and concerns as they arise.

12. Statement of Subject Rights

Your participation in this sub-study is entirely voluntary. If you decide not to take part or to withdraw from the sub-study at any time, you will not be penalised nor lose any benefits to which you are otherwise entitled.

Your doctor will have explained the details of this trial to you and answered any questions you may have. You should be satisfied with the information you have been given and had adequate time to consider whether you want to participate. If you decide you would like to take part in this sub-study, you will be asked to sign a consent form. If you do not want to take part, or if you choose to withdraw from the trial at any time, you will continue to receive the best medical care offered by your doctor.

Your doctor may decide to withdraw you from the sub-study and continue to care for you if, for example, your condition worsens or if you experience adverse effects during participation in the sub-study. You are encouraged to contact your doctor should you decide not to continue your participation in the sub-study. Your doctor will explain the best way for you to discontinue your participation in the research sub-study.

All information obtained in connection with this sub-study will remain confidential. You will be identified by your initials and a subject number (assigned to you as part of the trial) on data collection forms and laboratory results. You will not be identified in any publication or public presentation of data from this sub-study. Only authorised personnel from the NCHECR (the sponsor's monitors and auditors), the ethics committee and government regulatory authorities will have access to your medical records. By signing this form, you give permission that these authorised persons may access your medical records. However, your records will always be treated as strictly confidential.

If you would like more information about the sub-study, do not hesitate to ask your doctor: ……………. *or* study coordinator: *…………………*
Contact number

If you have a problem during the sub-study and would like to talk to an independent person you may contact:
The Executive Officer
Ethics Committee
Contact Number:

You will be given a copy of this form to keep.

CONSENT FORM

1. I, ... of .... *.., aged .... years, agree to participate in the sub-study described in the patient information statement set out in the attached form.*

2. I acknowledge that I have read the patient information statement, which explains why I have been selected, the aims of the experiment and the nature and the possible risks of the investigation, and the statement has been explained to me to my satisfaction.

3. Before signing this consent form, I have been given the opportunity of asking any questions relating to any possible physical and mental harm I might suffer as a result of my participation and I have received satisfactory answers.

4. I understand that I can withdraw from the sub-study at any time without prejudice to my relationship with my doctor.

5. I agree that research data gathered from the results of the sub-study may be published, provided that I cannot be identified.

6. I understand that if I have any questions relating to my participation in this research, I may contact *Doctor or Nurse* who will be happy to answer them.

7. I agree to have a blood sample collected and used for immunological testing related to abacavir and HIV ______(Patient initials)

   I **do not** agree to have a blood sample collected and used for immunological testing related to abacavir and HIV ______(Patient initials)

8. If I have a positive skin patch test:
   I agree to have a skin punch biopsy done and used for further testing ______(Patient initials)

   I **do not** agree to have a skin punch biopsy done and used for further testing ______(Patient initials)

9. I acknowledge receipt of a copy of this Consent Form and the Subject Information Statement.
   Complaints may be directed to Executive Officer, E*thics Committee.*

______________________________ ____________________________ ________
Signature of Participant Please PRINT name Date

______________________________ ____________________________ ________
Signature of Investigator(s) Please PRINT name Date

______________________________ ____________________________ ________
Signature of Witness Please PRINT name Date

____
Nature of Witness

CONSENT FORM

REVOCATION OF CONSENT

I hereby wish to **WITHDRAW** my consent to participate in the research proposal described above and understand that such withdrawal **WILL NOT** jeopardise any treatment or my relationship with my doctor.

____________________________ ________________________
Signature Date

____
Please PRINT Name

____
Signature of Investigator(s)

___________________________ _______________________
Please PRINT name Date

____
Signature of Witness

___________________________ _______________________
Please PRINT name Date

____
Nature of Witness

Appendix 2: Patch Test Instructions

INSTRUCTIONS FOR ABACAVIR PATCH TESTING

Patch Test Kit contents:
1 glass jar containing petrolatum ointment
1 glass jar containing 1% abacavir ointment
1 glass jar containing 10% abacavir ointment
Micropore tape
Black round medium pen
Kendall Versalon All-purpose sterile sponges
Applicator with cotton tip
Finn chambers for epicutaneous testing

Day 0 (first visit)<br>**PATCH PREPARATION**	Day 1 (reading 24 hours after patch application)<br>**INITIAL PATCH READING**	Day 2 (48 hours after patch application)<br>**PATCH READING AND REMOVAL**
• Verify contents of patch kit<br>• Kit including abacavir reagents should be stored at room temperature<br>• Patches should be<br>• prepared immediately before use<br>• Patches are applied to a clear and clean area in the mid upper back (between shoulder blades) unless the skin is excessively hairy (left upper arm is used when there is no other choice)<br>• Prior to patch application the back should be cleaned with warm water and/or hypoallergenic soaps with the sponges provided in the kit Patient should avoid vigorous exercise, sweating ,sun exposure or showering until after patches are removed<br>• In preparation of the patch peel off the backing paper of a four well piece of tape to expose the wells and the adhesive tape:	• Carefully peel back the left hand corner of the patch to make an assessment of whether there is a reaction or not<br>• A true reaction includes both erythema and induration.<br>• Reactions should be scored as doubtful, weak positive, strong positive, extreme positive or clear negative (see table below)<br>• A digital photograph should be taken to document any positive or suspected positive reaction.	• Carefully peel back the left hand corner of the patch to remove it.<br>• Wash and dry the patient's back with warm water and hypoallergenic soap<br>• Score the reaction as per the 24 hour reading<br>• A digital photograph should be taken of the back to document any reaction (positive or negative)<br>• Example of positive patch test (1% and 10% abacavir):<br>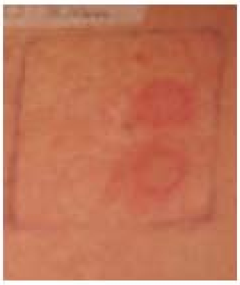<br>• Example of negative patch test (1% and 10% abacavir)

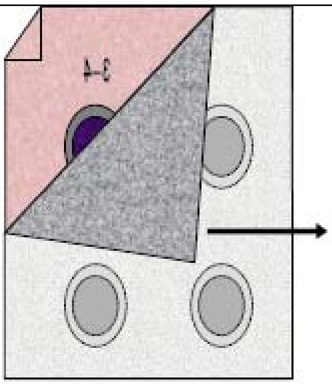<br>• Fold the exposed corner of the tape back on itself and attach the patch to the working surface to make it easier to fill the wells<br>• Load three of the four wells with the three samples (petrolatum, 1% abacavir and 10% abacavir)using the applicator with cotton tip, taking care to document on the patient record which reagent is in which well. Since turning over the tape will reverse the orientation of the two columns of wells this should be taken into account when filling the wells with abacavir reagent to document which reagent is filled where<br>• When filling the wells take care to load samples into the centre of the well allowing 2-3 mm of space around the edge of the sample to the lip of the well to allow to spread. Using this method should avoid under or overfilling the individual well<br>• After cleaning the patient's back prepare an identification label using a short length of hypoallergenic paper tape supplied to write the current date and time and affix to the nape of patient's neck<br>• Turn patch over and apply patch filled with petrolatum,		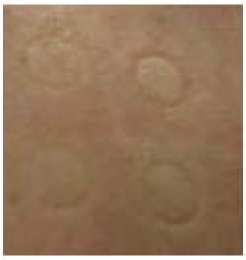

1% and 10% abacavir to patient's back under the label. Smooth the patch down to make sure the edges and patch wells are uniform and show good adhesion to the skin<br>• Use a pen to carefully draw around the edges of the patch (this will allow you to observe at followup visits whether a patch has moved for any reason).<br>• Once applied to the patient back and outlined with pen the patch should appear as follows:<br>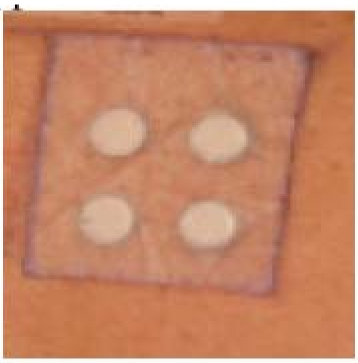		

Standard Patch Test Scores*

?	Doubtful (faint erythema only)
+	Weak positive (erythema, infiltration, possibly papules)
++	Strong positive (erythema, infiltration, papules, vesicles)
+++	Extreme positive (bullous, ulcerative)
-	Negative

*A digital photograph should be taken to verify patch test results.
