# Supplementary material for: Changes in Bone Turnover and Bone Loss in HIV-Infected Patients Changing Treatment to Tenofovir-Emtricitabine or Abacavir-Lamivudine
Source: PLoS One. 2012 Jun 15;7(6):e38377. doi: 10.1371/journal.pone.0038377 (PMC3376146; doi:10.1371/journal.pone.0038377)
Supplement: Protocol S2 — STEAL BMD Sub-Study - Concept Sheet. (PDF) [file pone.0038377.s002.pdf]

STEAL BMD Sub-Study - Concept Sheet

Proposing Investigators:

Professor Andrew Carr
St Vincent's Hospital and University of New South Wales, Sydney

Hila Haskelberg
National Centre in HIV Epidemiology and Clinical Research, University of New South Wales

Professor Jennifer Hoy
Alfred Hospital and Monash University, Melbourne

Professor Peter Ebeling
University of Melbourne, Melbourne

Professor Sean Emery
National Centre in HIV Epidemiology and Clinical Research, University of New South Wales

Background

Loss of bone mineral density (BMD) results in increased morbidity, mortality and health care-related costs, and lower quality of life. The National Health and Nutrition Examination Survey revealed 56% of women and 18% of men have osteopenia or osteoporosis.[1]

In numerous cross-sectional studies, HIV-infected adults have a higher prevalence of low BMD (40% to 83%) than the general population;[2-18] a 6.4-fold increased odds ratio of low BMD (T-score <-1.0) and a 3.7-fold increased odds ratio of osteoporosis (T-score <-2.5).[9] Factors associated with low BMD in these studies included traditional risk factors (increasing age, female sex, menopause, corticosteroid therapy, low body mass index, low serum testosterone level) as well as HIV-related factors (duration of HIV, elevated plasma HIV viral load, use of any antiretroviral therapy [ART], of thymidine nucleoside analogue therapy, and/or of protease inhibitor therapy [PI]).

Prospective studies, most of which were small and/or non-randomized, have generally found that ART reduces BMD. In the SMART study, continuous ART (VS group) was associated with significantly greater declines in spine and hip BMD relative to intermittent ART (DC group).[19] Estimated DC versus VS group differences in mean BMD change through follow-up were 1.4% (hip; 95%CI 0.5 to 2.3; p=0.002), 1.2% (spine by DXA; 95% CI 0.02 to 2.3, p=0.05), and 2.9% (spine by qCT; 95%CI 0.7 to 5.1, p=0.01).

Spine BMD (DXA)

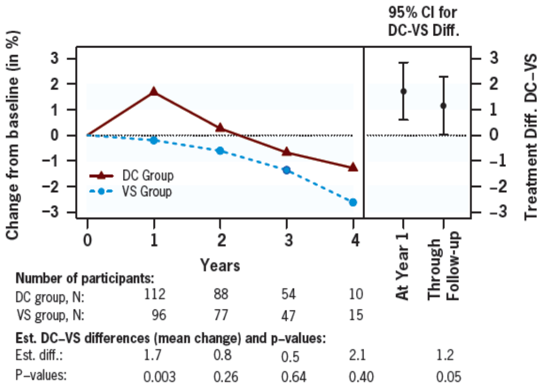

Hip BMD

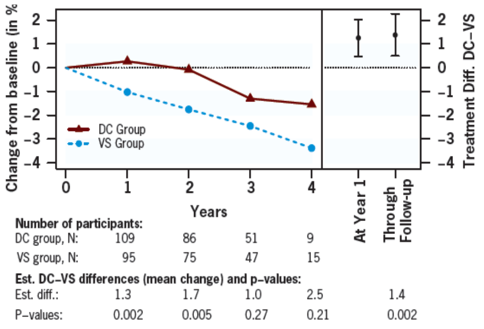

With continuous ART, the respective BMD declines per year in SMART were 0.9% (total hip), 0.4% (spine

DXA) and 2.9% (spine by qCT). These rates of decline appear to be greater than those observed in healthy men enrolled in the NHANES study, and more similar to those of (older) post-menopausal women.[1]

	BMD decline per year		
	**SMART VS (mean 45 yrs)**	**NHANES white men (35-55 yrs)**	**NHANES white women (55-75 yrs)**
**Hip BMD (DXA)**	0.9%	0.19%	0.78%
**Spine BMD (DXA)**	0.4%	0.24%	0.79%

The STEAL study compared TDF-FTC and ABC-3TC fixed dose combination-based therapy over 96 weeks when either fixed dose combination (FDC) was substituted for current NRTIs in 357 HLA-B*5701-negative adults with plasma HIV viral load <50 copies/mL.[20] TDF-FTC was associated with more bone loss than ABC-3TC, whereas cessation of current NRTI therapy (TDF 30%, AZT/d4T/ddI 50%) was associated with a significant improvement in BMD.

Change in right hip T-score

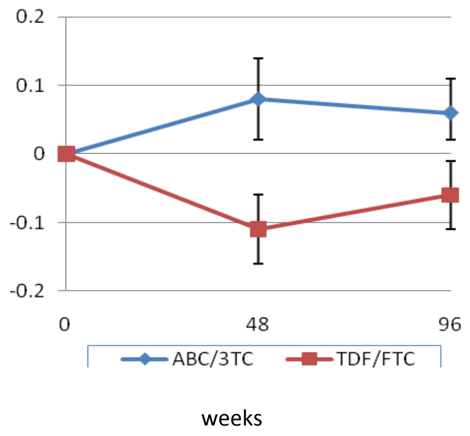

Change in spine T-score

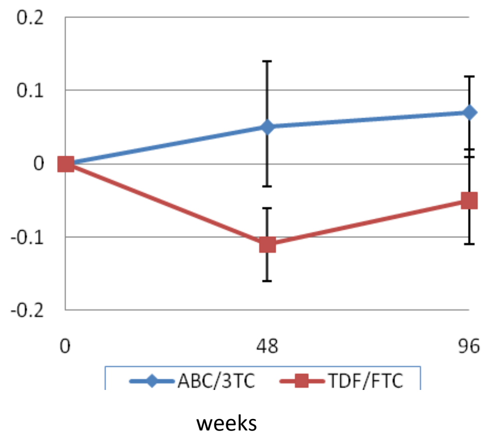

ABC-3TC	N=176	165	160	N=175	164	161
TDF-FTC	N=176	167	166	N=176	167	167

The only factor associated with greater BMD loss in prospective studies of HIV-infected patients is the use of tenofovir (TDF), although very few HIV studies have prospectively evaluated risk factors for BMD loss.[21-23] BMD declined in animals receiving doses of TDF that yielded a 6-fold higher plasma concentration than in humans receiving TDF 300mg daily. Plasma TDF levels increase by about 30% in patients receiving a PI.[24] No study has determined which subset of patients receiving TDF is most at risk of BMD loss.

There is no evidence that low BMD in an HIV-infected population will have a different biological significance to that in the general population. In a large population-based survey of 8,525 HIV-infected and 2,208,792 non HIV-infected individuals, fractures were more common in HIV-infected patients, regardless of sex and race.[25] Participants in SMART receiving continuous ART had a significantly higher incidence of serious fractures (rate of 0.13 fractures per 100 person years) as compared to those in the

intermittent ART arm (rate of 0.03 fractures per 100 person years) (hazard ratio 4.9 [95% CI 1.1 to 22.5]; P=0.04).[19]

Fracture risk is not only dependent on BMD, but also on multiple other risk factors including age, sex, personal and family history of fracture, corticosteroid use, alcohol use, and smoking. The World Health Organization (WHO) recently issued the FRAX equation (available at: http://www.shef.ac.uk/FRAX/) that can be used to estimate the 10-year risk of fracture (hip fracture and major osteoporotic fracture) based on all these key risk factors. An additional Fracture Risk Calculator has been developed using data collected in the Dubbo Osteoporosis Epidemiology Study, however this calculator only applies to men aged 60 and above, and therefore will not be used in this sub-study. Both these tools require additional clinical data that were not collected in STEAL: prior personal history of fracture, prior family history of hip fracture, and degree of alcohol use.

Use of the FRAX equation may identify a greater or different proportion of patients at risk of fracture than are identified by use of BMD only. In an analysis of 153 outpatients attending the HIV clinic at St Vincent's Hospital, Sydney, 4.3% had osteoporosis whereas 16% had a FRAX score greater than 7.5% ($p<0.001$; Alexandra Calmy, unpublished data). In addition, this may provide better insight into the clinical significance of low BMD seen in the context of chronic ART. For example, antiresorptive therapy has been estimated to be cost-effective in those with a 10-year risk >3%.[26]

The maintenance of bone homeostasis occurs by a finely co-ordinated balance between osteoclast-mediated bone resorption and osteoblast-mediated bone formation. The number, maturation and activity of osteoblasts and osteoclasts are regulated by a complex arrangement of intercellular signaling. Bone remodeling is mediated by changes in levels of RANKL (receptor of nuclear factor-κB ligand, produced by activated T cells and osteoblasts) and osteoprotegerin (a TNF receptor, prevents the RANKL-RANK interaction and inhibits osteoclast differentiation). Reports on the effect of combination ART on markers of bone metabolism in adults are contradictory[18,27-30],but most studies report increased bone turnover (increased levels of bone formation markers and increased bone resorption). High bone turnover is an independent predictor of fragility fractures, another important consideration in HIV [31].

There are no prospective studies of markers of bone turnover or of bone remodeling regulation in the setting of HIV and ART. The contributions of HIV-induced T-cell expression of inflammatory cytokines such as interleukin-1 (IL-1), interleukin-6 (IL-6) and TNF-α on osteoclast differentiation and bone resorption have not been adequately evaluated. Higher circulating levels of IL-6 are independently associated with greater BMD loss in the general population [32]. It is possible if not probable that both HIV and ART affect the paracrine regulation of bone metabolism, in addition to effects of T-cell activation and inflammation.

In STEAL, plasma from 20ml blood and serum from 20ml blood was stored at baseline, and at weeks 12, 24, 48, 72, and 96 on 323 of the 357 patients randomised. All stored samples except at week 12 were collected after a minimum 10-hour overnight fast, and the time of collection has been documented. All 323 patients randomised had at least another sample stored at one of the study visits, in addition to the ones collected at baseline. As some of the markers for measurement of bone turnover (particularly βCTX) undergo diurnal variation,[31] sampling time window will be considered at the statistical analysis and adjusted if sample was taken out of the regular time window per patient (approx. 4 hours). 38 patients of the 357 randomised changed their randomly assigned therapy during the period of follow up. For these patients, samples collected after their ART change will not be included in the analysis.

Table 1 The biochemical markers to be evaluated in this proposal (assays CVs are detailed in Appendix 1):

Category	Marker	Principle	Instrument	Manufacturer	Sample type	volume	Cost
Bone Resorption	C-terminal cross-linking telopeptide of type 1 collagen (βCTX)	ECLIA*	E170 immunoassay analyzer	Roche, Mannheim, Germany	Serum	300 µL	$15
Bone Formation	Procollagen type 1 N-terminal propeptide (P1NP)						$15
	Bone Specific ALP	IEMA***	Manual with Plate Reader	IDS (immunodiagnostic systems) Boldon, United Kingdom	Serum	200 µL	$30
Bone Turnover Regulators	Osteoprotegerin (OPG)	IEMA	Manual with Plate Reader		Serum	200 µL	$30
	Receptor activator of nuclear factor kappa (RANK) Ligand	IEMA	Manual with Plate Reader		Serum	300 µL	$30
	Total testosterone	ECLIA*	E170 immunoassay analyzer	Roche, Mannheim, Germany	Serum	1 mL	$10.5
	SHBG						$10.5
	Oestradiol						$10.5
	Free testosterone	Is calculated using the assay results for total testosterone and SHBG according to the Vermueulen formula					$10.5
	25(OH) D	Competitive CLIA**	Liasion	DiaSorin, Inc., Stillwater, MN	Serum	300 uL	$32
Systemic Inflammation	immune activation markers (IL6, TNF-α)^						
					TOTAL PER PATIENT PER VISIT	2.3mL	$194
					TOTAL COST PER PATIENT PER STUDY (x6 visits)	$794	
					COST FOR approx.1894 SAMPLES	**$ 251,182**	

*ECLIA = **e**lectro**c**hemi**l**uminescence **i**mmuno**a**ssay, **CLIA = chemiluminescence immunoassay
***IEMA = Immunoenzymetric Assay
^ *Results for these measurements will be obtained from the STEAL cardiovascular markers sub-study*

Primary Hypothesis

That early changes in markers of BMD turnover (as listed in Table 1) will predict the extent of the hip BMD loss over 96 weeks in those receiving TDF-based ART vs. ABC-based ART

Primary Analysis

To determine if early (weeks 12 and 24) changes from baseline in markers of BMD turnover (Table 1), predict changes in hip BMD (percentage change, as measured by DEXA) over 96 weeks in those receiving TDF-based ART vs. ABC-based ART.

Secondary Analyses

A number of secondary endpoints will be examined and compared by randomised treatment arm. These will include but will not be limited to the following:

Change in biomarkers:

1. To determine mean change by randomised arm in markers of bone turnover (Table 1) over 96 weeks.

Predictors of change in BMD:

2. To determine if early (weeks 12 and 24) changes in markers of BMD turnover, bone regulation and systemic inflammation predict changes in spine BMD over 96 weeks in those receiving TDF-based ART vs. ABC-based ART.
3. To determine the baseline patient and biochemical characteristics associated with greater loss of hip BMD (percentage change, as measured by DEXA) from baseline to 96 weeks.

   Factors to be considered in univariate analysis prior to multivariable analysis are:

   - Randomised therapy (TDF-FTC-based ART vs. ABC-3TC-based ART)
   - Demographics (age, sex, race)
   - Body composition (weight, body mass index, anthropometry, body fat)
   - HIV disease (duration, CD4 cell count, AIDS/non-AIDS)
   - ART prior to baseline (duration prior ART, PI at baseline [yes/no]; NRTI type at baseline [ABC, TDF, other])
   - Other risk factors for low BMD
     - Smoking
   - Baseline biochemical parameters (e.g. calcium, total and bone-specific alkaline phosphatase, phosphate, creatinine, GFR)
   - Baseline Testosterone (free), serum 1,25-dihydroxyvitamin D_3, Estradiol
   - Early changes in serum markers of bone turnover:
     - P1NP
     - βCTX (carboxy-terminal cross-linking telopeptide of type I collagen)
   - Baseline paracrine regulators of bone turnover (osteoprotegerin, RANK-L, RANK-L/osteoprotegerin ratio)
   - Baseline and on-study immune activation markers (IL6, TNF-α)
4. To determine the baseline patient and biochemical characteristics (listed above 3.) associated with greater loss of spine BMD (percentage change, as measured by DXA) in patients receiving TDF-FTC and ABC-3TC-based ART from baseline to 96 weeks.

5. To determine risk factors (predictors) for the development of low BMD (hip or lumbar spine t-score <-1).

Clinical Outcomes:

6. To determine the proportion of patients by randomised arm with prevalent osteopenia (hip or lumbar spine t-score <-1 but neither <-2.5) and osteoporosis (hip or lumbar spine t-score <-2.5) at baseline.
7. To determine the incidence of osteopenia and osteoporosis over 96 weeks by randomised arm
8. To estimate and compare changes in 10-year fracture risk (using the FRAX equation) with TDF-FTC and ABC-3TC-based ART from baseline to 96 weeks.
9. To determine, by randomised arm, the proportion of participants with FRAX score above the threshold recommended for intervention with antiresorptive therapy by the US National Osteoporosis Foundation guidelines
   9.1. 10-year hip fracture risk >3%
   9.2. 10-year major fracture risk >20%

Exploratory Analyses

10. Correlation analyses
    10.1. Correlate TWAUC, in the serum markers of bone turnover (Table 1) and mean percentage change in BMD over 96 weeks.
    10.2. Correlate TWAUC, in the serum markers of bone turnover (Table 1) and mean percentage change in BMD over 96 weeks stratified by randomised.
11. To determine and compare the mean percentage change in hip and lumbar spine BMD and bone markers from baseline over 96 weeks stratified by prior exposure to TDF and ABC at baseline.

Statistical analysis

Comparison of mean change in biomarkers by study arm will be assessed by t-test. For any stratified analysis, a test for interaction will be conducted. Predictors of continuous and binary outcomes will be determined using linear and logistic regression techniques respectively. Multivariable analysis will be conducted using forward stepwise method. Parameters that achieve a p value <0.1 in univariable analysis will be assessed in a multivariable analysis.

Comparison of binary outcomes (osteopenia and osteoporosis) will be analysed using chi-square tests.

Correlation analysis between changes in serum markers of bone turnover and mean percentage change in BMD will be undertaken using Pearson's correlation or non-parameteric equivalent as appropriate.

All analyses will be by per protocol to evaluate the biological effects of the intervention.

References

1. Looker AC, Wahner HW, Dunn WL, et al. Updated data on proximal femur bone mineral levels of US adults. Osteoporos Int 1998; 8: 468-89.

2. Tebas P, Powderly WG, Claxton S, et al. Accelerated bone mineral loss in HIV-infected patients receiving potent antiretroviral therapy. AIDS 2000; 14: F63-67.

3. Moore AL, Vashisht A, Sabin CA, et al. Reduced bone mineral density in HIV-positive individuals. AIDS 2001; 15: 1731-3.

4. Bruera D, Luna N, David DO, Bergoglio LM, Zamudio J. Decreased bone mineral density in HIV-infected patients is independent of antiretroviral therapy. AIDS 2003; 17: 1917-23.

5. Amiel C, Ostertag A, Slama L, et al. BMD is reduced in HIV-infected men irrespective of treatment. J Bone Mineral Res 2004; 19: 402-9.

6. Brown TT, Ruppe MD, Kassner R, et al. Reduced bone mineral density in human immunodeficiency virus-infected patients and its association with increased central adiposity and postload hyperglycemia. J Clin Endocrinol Metab 2004; 89: 1200-6.

7. Dolan SE, Huang JS, Killilea KM, Sullivan MP, Aliabadi N, Grinspoon S. Reduced bone density in HIV-infected women. AIDS 2004; 18: 475-83.

8. Yin M, Dobkin J, Brudney K, et al. Bone mass and mineral metabolism in HIV+ postmenopausal women. Osteoporos Int 2005; 16: 1345-52.

9. Brown TT, Qaqish RB. Antiretroviral therapy and the prevalence of osteopenia and osteoporosis: a meta-analytic review. AIDS 2006; 20: 2165-74.

10. Fausto A, Bongiovanni M, Cicconi P, Menicagli L, Ligabò EV, Melzi S, et al. Potential predictive factors of osteoporosis in HIV-positive subjects. Bone 2006; 38: 893-97.

11. Jones S, Restrepo D, Kasowitz A, Korenstein D, Wallenstein S, Schneider A, et al. Risk factors for decreased bone density and effects of HIV on bone in the elderly. Osteoporos Int 2008; 19: 913-18.

12. Carr A, Miller J, Eisman J, Cooper DA. Osteopenia in HIV-infected men: association with asymptomatic lactic acidemia and lower weight pre-antiretroviral therapy. AIDS 2001; 15: 703-09.

13. Bolland MJ, Grey AB, Horne AM, et al. Bone mineral density is not reduced in HIV-infected Caucasian men treated with highly active antiretroviral therapy. Clinical Endocrinology 2006; 65: 191-7.

14. Dolan SE, Carpenter S, Grinspoon S. Effects of weight, body composition, and testosterone on bone mineral density in HIV-infected women. J Acquir Immun Defic Syndr 2007; 45: 161-67.

15. Arnsten JH, Freeman R, Howard AA, Floris-Moore M, Lo Y, Klein RS. Decreased bone mineral density and increased fracture risk in aging men with or at risk for HIV infection. AIDS 2007; 21: 617-23.

16. Fernandez-Rivera J, Garcia R, Lozano F, et al. Relationship between low bone mineral density and highly active antiretroviral therapy including protease inhibitors in HIV-infected patients. HIV Clin Trial 2003; 4: 337-46.

17. Cazanave C, Dupon M, Lavignolle-Aurillac V, et al. Reduced bone mineral density in HIV-infected patients: prevalence and associated factors. AIDS 2008; 22: 395-402.

18. Mondy K, Yarasheski K, Powderly WG, et al. Longitudinal evolution of bone mineral density and bone markers in human immunodeficiency virus-infected individuals. Clin Infect Dis 2003; 36: 482-90.

19. Grund B, Isaakson R, Peng G, Ellis K, Gibert C, Shlay J, Drummond F, Martinez E, Hoy J, Reiss P, El-Sadr WM, Visnegarwala F, Carr A, for the SMART/INSIGHT group. Continuous antiretroviral therapy decreases bone mineral density: a SMART substudy. In: Abstracts of the 10th Workshop on Adverse events and Lipodystrophy in HIV, London, United Kingdom, 6-8 November, 2008.

20. STEAL study group (CROI, Montreal 8-11 February, 2009, abstract 756).

21. Gallant JE, Staszewski S, Pozniak AL, et al. Efficacy and safety of tenofovir DF vs stavudine in combination therapy in antiretroviral-naive patients: a 3-year randomized trial. JAMA 2004; 292: 191-201.

22. Duvivier C, Kolta S, Assoumou L, et al. First line PI-containing regimens cause a greater decrease in bone mineral density compared NNRTI-containing regimens in HIV-1 infected patients: a substudy of HIPPOCAMPE–ANRS

121 trial. In: Abstracts of the 15th Conference on Retroviruses and Opportunistic Infections, Boston, USA, February, 2008 (abstract 967).

23. Rivas P, Gorgolas M, Garcıa-Delgado R, et al. Evolution of bone mineral density in AIDS patients on treatment with zidovudine/lamivudine plus abacavir or lopinavir/ritonavir. HIV Med 2008; 9: 89–95.

24. Van Rompay KK, Brignolo LL, Meyer DJ, et al. Biological effects of short-term or prolonged administration of 9-[2-(phosphonomethoxy)propyl]adenine (tenofovir) to newborn and infant rhesus macaques. Antimicrob Agent Chemother 2004; 48: 1469-87.

25. Triant VA, Brown TT, Lee H, Grinspoon SK. Fracture prevalence among human immunodeficiency virus (HIV)-infected versus non-HIV-infected patients in a large U.S. healthcare system. J Clin Endocrinol Metab 2008; 93: 3499-504.

26. Dawson-Hughes B, Tosteson ANA, Melton III LJ, Baim S, Favus MJ, Khosla S, Lindsay RL. Implications of absolute fracture risk assessment for osteoporosis practice guidelines in the USA. Osteoporosis Int 2008; 19: 449-458..

27. Aukrust P, Haug CJ, Ueland T, Lien E, Muller F, Espevik T*, et al.* Decreased bone formative and enhanced resorptive markers in human immunodeficiency virus infection: indication of normalization of the bone-remodeling process during highly active antiretroviral therapy. *Journal of Clinical Endocrinology & Metabolism* 1999,84:145-150.

28. Teichmann J, Stephan E, Discher T, Lange U, Federlin K, Stracke H*, et al.* Changes in calciotropic hormones and biochemical markers of bone metabolism in patients with human immunodeficiency virus infection. *Metabolism: Clinical & Experimental* 2000,49:1134-1139.

29. Konishi M, Takahashi K, Yoshimoto E, Uno K, Kasahara K, Mikasa K. Association between osteopenia/osteoporosis and the serum RANKL in HIV-infected patients. *AIDS* 2005,19:1240-1241.

30. Seminari E, Castagna A, Soldarini A, Galli L, Fusetti G, Dorigatti F*, et al.* Osteoprotegerin and bone turnover markers in heavily pretreated HIV-infected patients. *HIV Medicine* 2005,6:145-150.

31. Vasikaran SD. Utility of biochemical markersof bone turnover and bone mineral density in management of osteoporosis. *Crit Rev Clin Lab Sci* 2008; 45: 221-258

32. Ding C, Parameswaran V, Udayan R, Burgess J, Jones G. Circulating levels of inflammatory markers predict change in bone mineral density and resorption in older adults: a longitudinal study. *Journal of Clinical Endocrinology & Metabolism* 2008,93:1952-1958.

Appendix 1 Assay CV details

Inter-assay CV (real time):

	**Conc**	**CV (%)**	**N**
Total testosterone (nmol/L)	5.1	5.3%	578
	19.7	3.5%	580
	42.5	2.9%	580
SHBG (nmol/L)	19.5	3.3%	586
	41.1	3.1%	592
Oestradiol (pmol/L)	147	8.8%	618
	1205	3.8%	626
	2278	4.1%	644
25OHD (nmol/L)	65	8.9%	97
	119	6.0%	97
	189	5.8%	97

Inter-assay CV (Manufacturer's Data)

	**Conc**	**CV (%)**
CTX (µg/L)	0.10	7.6%
	0.41	4.2%
	1.53	2.7%
P1NP (µg/L)	57.2	3.8%
	226	4.0%
	1093	4.2%
Bone Specific ALP (µg/L)	8.4	5.8%
	29.2	6.4%
	55.6	3.7%
	81.1	6.1%
OPG (pmol/L)	5.53	7%
	10.1	8%
RANKL (pmol/l)	1.60	6%
	1.42	3%

The cvs in yellow represent the "working range" for the assays.
